# Supplementary material for: Building an explanatory model for snakebite envenoming care in the Brazilian Amazon from the indigenous caregivers’ perspective
Source: PLoS Negl Trop Dis. 2023 Mar 10;17(3):e0011172. doi: 10.1371/journal.pntd.0011172 (PMC10047533; doi:10.1371/journal.pntd.0011172)
Supplement: S1 File — (DOCX) [file pntd.0011172.s001.docx]

**Consolidated criteria for reporting qualitative studies (COREQ): 32-item checklist**

Developed from:

Tong A, Sainsbury P, Craig J. Consolidated criteria for reporting qualitative research (COREQ): a 32-item checklist for interviews and focus groups. *International Journal for Quality in Health Care*. 2007. Volume 19, Number 6: pp. 349 – 357

| **No. Item** | **Guide questions/description** | **Reported on Page #** |
| --- | --- | --- |
| **Domain 1: Research team and reﬂexivity** |  |  |
| *Personal Characteristics* |  |  |
| 1. Inter viewer/facilitator | *Which author/s conducted the interview or focus group?* | Line 172 |
| 2. Credentials | *What were the researcher’s credentials?* | Line 173 |
| 3. Occupation | *What was their occupation at the time of the study?* | Title page and line 174 |
| 4. Gender | *Was the researcher male or female?* | Line 172 |
| 5. Experience and training | *What experience or training did the researcher have?* | Lines 172-174 |
| *Relationship with participants* |  |  |
| 6. Relationship established | *Was a relationship established prior to study commencement?* | Lines 195-196 |
| 7. Participant knowledge of the interviewer | *What did the participants know about the researcher? (e.g. personal goals, reasons for doing the research).* | N/A |
| 8. Interviewer characteristics | *What characteristics were reported about the inter viewer/facilitator? e.g. Bias, assumptions, reasons and interests in the research topic.* | Lines 207-208 |
| **Domain 2: study design** |  |  |
| *Theoretical framework* |  |  |
| 9. Methodological orientation and Theory | *What methodological orientation was stated to underpin the study? e.g. grounded theory, discourse analysis, ethnography, phenomenology, content analysis* | Lines 144-151 |
| *Participant selection* |  |  |
| 10. Sampling | *How were participants selected? e.g. purposive, convenience, consecutive, snowball* | Lines 187-189 |
| 11. Method of approach | *How were participants approached? e.g. face-to-face, telephone, mail, email* | Lines 185-195 |
| 12. Sample size | *How many participants were in the study?* | Line 190 |
| 13. Non-participation | *How many people refused to participate or dropped out? Reasons?* | Line 195 |
| *Setting* |  |  |
| 14. Setting of data collection | *Where was the data collected? e.g. home, clinic, workplace* | Lines 201-202 |
| 15. Presence of non-participants | *Was anyone else present besides the participants and researchers?* | Line 202 |
| 16. Description of sample | *What are the important characteristics of the sample? e.g. demographic data, date* | Lines 237-242 |
| *Data collection* |  |  |
| 17. Interview guide | *Were questions, prompts, guides provided by the authors? Was it pilot tested?* | Lines 200 -201 |
| 18. Repeat interviews | *Were repeat inter views carried out? If yes, how many?* | N/A |
| 19. Audio/visual recording | *Did the research use audio or visual recording to collect the data?* | Lines 205-206 |
| 20. Field notes | *Were ﬁeld notes made during and/or after the interview or focus group?* | Lines 206-207 |
| 21. Duration | *What was the duration of the interviews or focus group?* | Lines 205-206 |
| 22. Data saturation | *Was data saturation discussed?* | Lines 1066-1073 |
| 23. Transcripts returned | *Were transcripts returned to participants for comment and/or correction?* | N/A |
| **Domain 3: analysis and ﬁndings** |  |  |
| *Data analysis* |  |  |
| 24. Number of data coders | *How many data coders coded the data?* | Line 221 |
| 25. Description of the coding tree | *Did authors provide a description of the coding tree?* | Line 221 |
| 26. Derivation of themes | *Were themes identiﬁed in advance or* *derived from the data?* | Lines 224-228 |
| 27. Software | *What software, if applicable, was used to manage the data?* | Line 208 |
| 28. Participant checking | *Did participants provide feedback on the ﬁndings?* | N/A |
| *Reporting* |  |  |
| 29. Quotations presented | *Were participant quotations presented to illustrate the themes/ﬁndings? Was each quotation identiﬁed? e.g. participant number* | Results section |
| 30. Data and ﬁndings consistent | *Was there consistency between the data presented and the ﬁndings?* | Discussion section |
| 31. Clarity of major themes | *Were major themes clearly presented in the ﬁndings?* | Results section |
| 32. Clarity of minor themes | *Is there a description of diverse cases or discussion of minor themes?* | N/A |
