# Supplementary material for: Building an explanatory model for snakebite envenoming care in the Brazilian Amazon from the indigenous caregivers’ perspective
Source: PLoS Negl Trop Dis. 2023 Mar 10;17(3):e0011172. doi: 10.1371/journal.pntd.0011172 (PMC10047533; doi:10.1371/journal.pntd.0011172)
Supplement: S2 File — (PDF) [file pntd.0011172.s002.pdf]

Entrevista Cuidador 1

Idade: 50 anos
Religião: Ordem da Santa Cruz (Cruzada)

Informações:

a. Gosta de ser chamado de curador. Explicou que o rezador não sabe feitiçaria, então ele não é forte o suficiente para combater o mal, mas ajuda as pessoas, principalmente crianças. O pajé sabe usar o poder, porque ele tira o mal e se protege. O curador sabe remédio para todas as doenças e dores, pratica o bem da cura o tempo todo para ajudar as pessoas.

b. Dependendo da doença a dieta alimentar pode durar dias. Em relação à medicina tradicional, deu ênfase no uso de cascas amargas, como da árvore de manga, casca de laranja, copaíba. Além disso, utiliza tabaco, breu branco e casa de abelha para defumações de ambientes e pessoas doentes.

[00:00:02]

A: Vamos lá::... na sua Aldeia acontece acidentes por picada de cobra? (...)

OF1: (...) É::...

A: Conta aí para mim como é que, quando acontece o quê que vocês fazem? Como é que eles... como é que vocês atendem?

OF1: Esse::... é que jeito do picada de cobra que quando nós, nós andemo lá né, lá no centro se acontecer esse daí (...)

A: (...) uhum.

OF1: mas antes ninguém sabe né? Daí ninguém sabe o que que tem, o que que é pra curar picada de cobra, né? (...)

A: (...) mas... mas acontece acidente por picada de cobra? Onde acontece?

OF1: Lá no centro (inint) [00:0048]. Por aí, por aí... quatro mil quilômetros assim nós tamos (alagando) [00:00:51] de Buritizal. Lá no chavascal [00:0055], por aí que acontece com pessoa.

A: E quando acontece lá nesse lugar, o que que vocês vão fazer lá?

OF1: Aí nós procuremo o cipó, cipó né... aí::... outra pessoa que tá procurando açaí (...)

A: (...) ah.

OF1: E burití... esse daí.

A: Ah, quando você... acontece os acidentes, as picadas de cobra quando vocês tão trabalhando buscando fruta?

OF1: É, no::... tem outra pessoa também acontece com:::: como que chama a:: capinando (...)

A: (...) na roça.

OF1: Na roça... aí que acontece daí (...)

A: (...) é

OF1: na mão, na perna... aí que acontece morde, morde cobra. Aí depois isso daí correndo, corre... até... para casa, né?

A: Quando acontece um acidente nesse lugar distante, aí vocês voltam para casa?

OF1: Volta para casa.

A: É? O senhor já viu algum acidente desse?

OF1: já. Já.

A: Já? Como é que foi esse acidente? O quê que a pessoa tava fazendo? Conta aí pra mim ouvir.

OF1: Esse daí que que tá acontecendo nada, apenas... ninguém sabe o que que é:::: esse (dor) [00:02:14] de cobra. Esse daí já aconteceu com ele, né? Esse daqui me contô::: não sabe, não sabe dor aquele... aquele:::... lugar do dente, não sabe dor, só aqui na cabeça que tem dor.

A: Ah. No local da picada não doía, doía só na cabeça? (...)

OF1: (...) Só na cabeça “aqui”. Assim oh.

A: E que cobra que picou ele?

OF1: Cobra::: aquele venenoso... aquele::: aquele cascavel e outra aquele:: cobra::: cobra mermo aquele outro, né? (...)

A: (...) Venenoso (...)

OF1: (...) Venenoso. Toda cobra que::: cobra tem dor. Esse daqui me falou: ai::: comigo:: comigo não nunca aconteceu isso daí. Meu sobrinho me falou assim: muito dor:: só na cabeça, mas não... na lugar do dente não é dor não.

A: não dói no den... não dói na::... no local da picada, só dá dor de cabeça?

OF1: Só... só dor de cabeça que ninguém aguentô, vomitando com sangue e... isso daí que é o problema.

A: E aí... esses casos assim o pajé atendeu ele? O curador atendeu?

OF1: Esse daqui quando::... quando chegou... primeiro no hospital, né?

A: uhum.

OF1: E... aí que::... (...)

A: (...) Trouxeram lá. Aconteceu lá no Buritizal e aí trouxeram pro hospital? (...)

OF1: (...) Trouxeram pro hospital (...).

A: E antes disso de chegar no Hospital o:: pajé fez alguma intervenção, alguma::... (...)

OF1: (...) Mais ninguém, ninguém, ninguém só depois só depois quando já... já passei dor né aí tomar um remédio e... o médico que dá o::... o remédio para passar dor né. Aí... aí depois de lugar::... lugar donde está o picada de cobra, aí que uma coisa assim::.. mexendo na coisa assim::... assim como assim... como outra coisa que se entra no seu coisa, né? se cuida, né? Ficou assim, assim tudo tempo. Esse daí tá coçando [00:04:30]. Num... num dá pra:: comer remoso aí esse daí tá::... dá o veneno, sai veneno, sai ferida... sai tudo. Outra coisa...teu carne ficou... [00:04:48] ficou podre assim.

A: Apodreceu o pé?

OF1: Apodreceu o pé. Assim:: por causa de comida né por aí.

A: quando ele comeu comida remosa. Que comida remosa que ele comeu? (...)

OF1: (revisar) 00:05:01 (...) pirapitinga, pirarucu, aruanã e::... pacú, o piau aquele... aquele... piau mermo aquele grandão, esse daí não presta para comer isso daí é remoso.

A: então quando (...)

OF1: (...) o frango, o frango isso daí não presta pra comer não. Esse daí ta coçando e da dor. E::... só bom que é traíra:: traíra (...)

A: (...) traíra pode comer?

OF1: pode comer traíra e... e curimatã.

A: curimatã também pode.

OF1: E sardinha (...)

A: (...) Sardinha também pode.

OF1: esse peixe que é bom. Esse daí não.... não... não dá problema né.

A: E se comer esses peixes remosos o quê que acontece com ele?

OF1: E... que acontece né, esse daí inchar:: inchado e... ficar doendo assim como... (inint) [00:05:58] nesse se... se mordeu assim que aconteceu.

A: é, quando come fica como se tivesse sendo picado de novo.

OF1: De novo... assim memo.

A: E por que que as cobras picam os::... as pessoas? Como que cê entende isso?

OF1: É:: aí que esse... esse cobra, esse cobra se você não mexe (...)

A: (...) aham.

OF1: se você não mexe pode bem aqui tem (terçado aqui) [00:06:25] assim, mas esse daí:: num... num morde gente no. Quando assim topar com...com um pouquinho assim aí cobra te mordeu.

A: cobra morde.

OF1: morde.

A: se a gente deixar ela quieto no lugar dela (...)

OF1: (...) se deixa. Antes, tu viu antes né mata logo esse daí, mata logo.

A: entendi.

OF1: Que esse (inint) [00:06:52] esse contando... contando... assim né contando com a outra se tem muito muita coisa aí esperando a coisa assim outro lugar assim. Assim que acontece.

A: entendi::.. e tem época... quando que acontece? Com que frequência acontece esses acidentes? Tem mais época do ano quando tá verão, quando tá chu::... quando tá:: chuva, como é que é? Qual que é tempo?

OF1: Tem, tem. Qualquer hora se tem tempo mês... porém mês de agosto quando... quando... assim como agora né tá... tem tempo de verão assim ma.. esse daí. O tempo assim como agora o tempo de...o tempo de::...

A: (...) de chuva.

OF1: de chuva. Aí ninguém... ninguém vê né, daí para onde que tá o... cuidado né de lá. (sabe) [00:08:01] por aí na roça ou na rua, caminho assim... aí ninguém vê... ou de tarde assim, de tarde porém de 6 horas já tá... cobra já tá andando. (...)

A: (...) já tão andando.

OF1: (...) tão andando.

A: (...) 6 horas da tarde ou 6 horas a manhã (...)

OF1: (...)6 horas da tarde... 6 horas da tarde até 3 horas da manhã.

A: da manhã.

OF1: (...) da manhã. Esse andando, aonde tá o lugar e já tá saindo. Ele... ele procura também comida né? Que você pisou em cima dele aí eu morde.

A: (...) ele morde (...)

OF1: (...) ele morde.

A: E quando uma cobra::... (...)

OF1: mas assim oh (...)

A: (...) pode falar

OF1: mas esse cobra::... não morde qualquer hora:: se tem cobra ovado assim, tem ovo assim aí que ele bravo (...)

A: a::... a cobra quando cobra quando tá ovada ela é mais (...)

OF1: (...) braba, braba (...)

A: (...) brava.

OF1: aí que quando onde tem e tu tá trabalhando né

A: uhum.

OF1: trabalhando aqui, por aí ele tá bem::.. bem ali (inint) [00:09:18] morde.

A: ele morde:: tá. E... e quando o::.. uma pessoa é picado por uma cobra o que deve ser feito?

OF1: esse daí::.. acho que a pessoa tem... tem dor né. Aí mesmo ela::... desmaiou de repente dormindo porém assim né parece que tá morrendo.

A: e o quê que deve ser feito pra socorrer essas... essas pessoas?

OF1: é::.. que::... meu pai já faleceu esse daí me contou né? Tirar pouquinho, se não corre esse daí mata...mata esse cobra. Aí tira pouquinho carne dele, carne dele pouquinho tira, aí tu:: coloca onde tá o dente né...coloca (...)

A: (...) onde foi picado coloca (...)

OF1: onde foi picado cobra (...)

A: (...) mas é a carne da cobra ou é a pele da cobra? (...)

OF1: (....) carne, carne de cobra. Não a carne (...)

A: (...) a carne da cobra.

OF1: ai que esse carne chupou a... a.. o veneno dele. Chupou, chupou, chupou, chupou assim. Coloca pouquinho. Aí que já pouquinho parou né o dor.

A: uhum.

OF1: aí tu corre (...)

A: (...) corre pra casa.

OF1: corre pra casa. Esse ate pra chegar tua casa pra não sentir muito dor não. Esse daí (...)

A: (...) e o que mais pode ser feito além... além de colocar esse pedaço da cobra? Que outros::.. outras coisas podem ser feitas? (...)

OF1: e coisas podem assim. Tira aquele casca né amarra onde::.. onde:: tá o dor assim a::.. a casca dela. Tira e marra pra não subir o teu corpo assim marra com força né. amarra (...)

A: (...) amarra com o que?

OF1: com::... com::... com casca dela... com casca dela de cobra né. tira o casca marra rapidinho (...)

A: pra não subir (...)

OF1: (...) pra não subir o teu corpo veneno assim.

A: (...) veneno. Isso aí também resolve.

OF1: resolve.

A: resolve

OF1: resolve. E se também::.. se tu aguentar... pa aguentar né, pa aguentar tira pouquinho mais come a carne (...)

A: (...) o pedaço de carne da cobra.

OF1: (...) carne da cobra. Pouquinho pa aguentar.

A: e o que que faz?

OF1: esse daí pra parar de dor.

A: tudo é pra parar de doer?

OF1: parar de dor. Aí:: aí se que tu não para né. Agora nós já temo como que::.. como que chama aquele::.. todo animal tem fígado né.

A: sim

OF1: Aí tem uma coisa como é que chama aquele... aquele (...)

A: (...) Feu (...)

OF1: aquele... amargo aquele...

A: (..) o féu.

OF1: isso daí tira aquele (...)

A: (...) féu da cobra.

OF1: féu não é cobra não. Aquele outro... outro surucucu::.. surucucu grande:: cobra grande.

A: ah de uma outra cobra (...)

OF1: (...) outra cobra aquele cobra grande.

A: (...) esse faz o quê?

OF1: esse daí mata. Esse daí tira o... feu dele. Coloca como assim como esse daí umas 3 gota... 3 gota esse feuzinho aí. Aí toma. Parou rapidinho.

A: aí o::.. féu as três gotas do feu de uma outra cobra serve pra essa cobra daí.

OF1: sabe esse::... esse animal do paca. Você sabe paca?

A: sim.

OF1: Paca... paca esse daí tem.. tem esse ferrozinho aí. Esse daí. (...)

A: (...) Féu da Paca?

OF1: Feu da paca.

A: aí faz o que? A mesma coisa? (...)

OF1: (...) mesma coisa de quando picou a cobra né. rápido. Tu tem::.. chegou no teu casa aí:: da gota do água pouquinho assim toma. Nem dor, nem passa nada (...)

A: (...) nem precisa vir pro hospital?

OF1: nem precisa ir pra hospital. esse daqui é bom... Santo remédio dai.

A: e o que::... que outras situações que pode agravar piorar o::... a pessoa que é picado por cobra? O senhor já falou das comidas remosas, mas tem alguma outra coisa na casa ou na comunidade de vocês que também pode piorar que a gente não deve fazer quando é picado por cobra?

OF1: esse daqui::.. esse daqui sou eu e já sei... já sei, mas outra coisa não... não sei também.

A: é.

OF1: não sei também. Outra coisa não sei. Já... já contei esse daí já sei. (...)

A: (...) tá mais relacionado com as comidas.

OF1: se não::... complicado isso daí. E outra coisa se::.. já.. já falei já contei remoso não pode comer pode vir chegou no hospital, já chegou em casa tem que... ter que comer só boa comida (...)

A: (...) só boa comida.

OF1: boa comida. Até por ai três meses assim (...)

A: por três meses.

OF1: três mês. Três mês

A: e na casa dele ele pode tá normal (...)

OF1: (...) normal (...)

A: (...) com a família dele (...)

OF1: (...) nem... nem... a pessoa se tem irmão né, se tem irmã, tu tem pai não pode fazer assim. Disse tu não pode transar sua mulher aí esse daí que é problema também.

A: ah, não pode ter relação sexual (...)

OF1: (...) sexual.

A: o quê que acontece se a pessoa (...)

OF1: a pessoa que tão morde cobra esse daí que tão podrecendo. Até pa terminar se não (...)

A: (...) invadir por toda perna (...)

OF1: (...) toda a perna, toda a perna.

A: e por quanto tempo tem que ficar de:: (...)

OF1: tem que, tem que, tem::... aí, aí que chama pessoa pa... coisa (inint) [00:15:37].

A: o pajé faz o::.. (...)

OF1: (...) pra curar isso daí.

A: o quê que o pajé usa pra fazer esse...esse ritual?

OF1: aí que tem que curar com tabaco, com...com cachaça também se tem outro usa né pessoa...(...)

A: (...) também usa (...)

OF1: (...) pra chupar isso daí. Pra chupar aí parou. Parou de coisa daí, parou de podrecer assim.

A: Quais que são os tratamentos além dos que o senhor já relatou até aqui mais utilizados na comunidade assim pelos pajés, pelos curandeiros? e usando o que? O senhor já falou que pode usar a::.. própria cobra pele da cobra para fazer amarração (...)

OF1: (...) amarra, amarração.

A: pode comer um pedaço da cobra, pode colocar um pedaço da carne em cima. Além disso que outros tratamentos pode ser feito?

OF1: Tratamento depois pra chegar no hospital para poder chegar no Hospital. Aí da...da... o coisa remédio, aí pa passa, aí depois daí dá::.. se não... se não passa né? Esse daí todo tempo... todo tempo mexendo né. Daí se alguma coisa se comer remoso aí começa de novo. aí incha de novo... incha de novo assim. Ferida sai ferida por isso que tá proibido pa dar sexual do pessoa [00:17:25]

A: entendi. E quando o... quando o indígena é picado ele é trazido pro hospital, ele toma o remédio e ele volta pra comunidade.

OF1: aham.

A: o tratamento continua lá na comunidade com as... com as...com as::.. com os tratamentos caseiros? (...)

OF1: (...) tratamento caseiro.

A: que tratamento caseiro vocês fazem?

OF1: tratamento caseiro que...que::.. tomou remédio aquele:... o::.. casca do::... coisa. Esqueci aquele amargo assim, amargo (...)

A: (...) uma casca amarga.

OF1: Amargo aquele casca do manga:: casca do manga é:: casa:: fruta do laranja né. tira esse casca esse daí, junto com esse daí coloca aí toma esse daí.

A: faz o quê?

OF1: faz... faz... faz chá, faz chá toda de manhã, só de manhã sim até pa passar até pra (termina o coisa esse daí)[00:18:32]. Esse daí que é bom pra matar aquele::.. aquele coisa né de... de veneno se não... não faz assim tudo tempo já parei assim com esse daí já passou mais aí... aí que::que sai ferida.

A: ele volta.

OF1: volta. Ele volta de novo. Mas tem que:: tem que respeitar. Não usa:: usa a pessoa né até 3 meses.

A: (...) até três meses.

OF1: (...) até três meses quando você tão bom né.

A: que outras plantas o senhor acha que pode ser utilizado? vocês utilizam pra fazer esse tratamento.

OF1: tratamento::... Esse daí que tá onde... bem... bem ali tem aquele... aquele que como se chama::.. aquele que tá coçando ali... como que chama::.. esse daí que é bom pa curar:: para curar isso daí. Esquenta todo tempo de manhã (...)

A: (...) esquenta a folha.

OF1: (...) esquenta a folha , aí dobra assim, coisa assim. Três vezes por dia até sete dia.

A: por sete dias.

OF1: sete dia. Parou de dor, parou de inchar.

A: é:: quando acontecem os acidentes ofídicos no... as picada de cobra na aldeia, na comunidade, ele... deve ir pro hospital ou eles devem fazer o tratamento em casa ou deve fazer os dois? Como é que vocês veem isso?

OF1: Se não:: no hospital também já parou:: pa hospital, mas quando chegou na..na casa né. se mas não tem o dor só uma vez:: só no hospital e se não.. se não passa aí chegando a casa aí que faz remédio pa tomar não é muito não pouquinho assim só de amargo.

A: (...) o remédio caseiro.

OF1: caseiro amargo [00:20:29].

A: mas o senhor acha que quando for picado tem que levar para o hospital?

OF1: leva.

A: Aí quando ele volta para casa que faz tratamento caseiro?

OF1: tratamento... é só de (amargo) [00:20:40] assim ou também o casca de:: taperebá:: esse daí também é bom pa... pa... pa curar esse daí. Fazer chá de manhã e toma... tu não passa muito ferida não.

A: o senhor lembra de além do caso que o senhor já me contou de outros casos de picada de cobra que aconteceram assim e como que aconteceu na comunidade?::
De homem, de mulher, de criança (...)

OF1: tudo... tudo... tudo assim acontece (...)

A: (...) é?

OF1: mulher, o:: rapaz, com adulto (...)

A: o senhor lembra de um caso que o senhor poderia me contar? do que aconteceu que ele tava fazendo como aconteceu.

OF1: mas antes né

A: antes casos.

OF1: antes::.. eu não sabia como que.. que faz né. Daí como que faz eu não sei não, mas agora tô... tô nesse idade.

A: uhum.

OF1:Eu sei todo que acontece ~~encontrei~~ né na minha frente né. Eu sabia daí. Eu já contei
esse daí só isso, mas outra antes que acontece ninguém sabe como que daí que que faz.

A: o quê... que... o que a pessoa tem que fazer pra não ser picada pela cobra? Pra não ser:: que:: como que ela deve se proteger?

OF1: ah pra proteger né

A: uhum.

OF1: pra proteger pa::.. pa não chegar no hospital. E da::.. remédio de caseiro aquele:: tem pau né lá no centro, tem o coisa... casca amargo né. Esse daí que toma (...)

A: (...) mas esse (...)

OF1: (...) assim como dipirona esse daí.

A: ah, tem mesmo efeito da Dipirona pra não doer.

OF1: mesmo... mesmo do Dipirona mesmo. Toma pouquinho assim, esse daí é pra parar de dor... antes esse daí pra parar de dor, pa não chegar até:: porém assim:: por aí, por aí três meses por aí 4 ou 6 mês assim. Se... se... se mãe... mãe... mãe dele, ou pai dele senão coisa bem esse daí que tá uma carne podrecendo e o perna dele ficar feio né assim (...)

A: (...) entendi.

OF1: ficar torto assim.

A: o quê que acontece se não cuidar direito? O que que acontece?

OF1: não cuida::.. não cuida direito (...)

A: (...) aí fica como?

OF1: Esse daí que fica não é bom não... fica aleijado.

A: fica aleijado.

OF1: fica aleijado.

A: tá. E me fala uma coisa então assim. Isso::.. isso... que você me falou agora é quando a pessoa já foi picado pela cobra e tá tratando. Eu quero saber do senhor é::.. que coisas que pode ser feito pra que ele não chegue a ser picado, como que deve se proteger? andar no mato, no chavascal, ir pra roça, onde tem cobra e tudo mas pra não ser picado. Como que ele tem que se proteger pra não ser picado?

OF1: não ser picado né daí. Esse daí. Que procurando por aí né no chavascal né.

A: hum.

OF1: Tá andando, andando, andando por esses:: esse daí não acontecer nada. Que...Porque não vê nada né daí, num taí... coisa aí nem quis ou nem olhar nem mexer esse daí, mas todo::.. mas todo dia que tão tá::..tá de coisa né daí. Aonde vocês estão procurando olhando assim não...não é todo tempo (...)

A: (...) nem é todo lugar que tem a cobra.

OF1: (...) não é todo tempo. Se você tá por aí assim, coisa assim... vê aí agora. A pessoa só mata logo se vê aonde mata, não corre por isso que tá hoje (aconteceu) [00:25:12]. Ante::.. ele não sabe cuidar quando ele anda no::.. no cerrado né por aí assim andando no cerrado, mas nem... nem vê aí por aí no::.. na frente né. Aí que... que pisou tem que acontecer.

A: então só é picado quando você pisa na cobra?

OF1: só na pisa.

A: mas existe algum... algum trabalho que os pais, os pajés podem fazer pra proteger a... pra pessoa não ser picada porque tem pessoas que estão à comunidade trabalho na roça, eles pescam, caçam, andam por onde as cobras têm mas não são picado pela cobra (...)

OF1: (...) não, não.

A: que tipo de proteção essas pessoas têm?

OF1: esse::... esse tem... tem coisa né. Tem pessoa né. Tem pessoa que outra coisa de:: uma coisa né é::.. como que chama aquele:::... tá atrapalhando assim né da pessoa né. esse daí que tá cobra mordeu assim, mas não é muito não aquele pessoa que tem coisa que não é bom (...)

A: ah, quando uma pessoa não é boa. Tá atrapalhando o trabalho das outras pessoas aí a cobra pica (...)

OF1: (...) é, o trabalho. Esse daí, esse daí. Aquele coisa assim por ai assim. Pessoa que tá::.. feiticeiro que enfeitiçando com isso daí (...)

A: (...) hum.

OF1: esse daí que tá mordendo.

A: esse::.. o feiticeiro que tá mandando? Como que... como que o feiticeiro manda fazer isso?

OF1: num sei::.. pa... pra matar gente.

A: é? Por que que manda matar?

OF1: É::.. por esse é::.. alguma coisa tem né. se ver alguma coisa ou tem muito dinheiro. É ele é muito invejoso [00:27:21] esse... esse feitiço todo tempo.

A: entendi ele faz um fei...(...)

OF1: (...) Aí que (inint) [00:27:25] (...)

A: (...) tem inveja da... (...)

OF1: (...) tem inveja da dele que tão já por ai assim... acontece com cobra e cobra:: e cobra nunca.. nunca que mordeu a pessoa não (inint) [00:27:41] tem como mandar (oração) [00:27:43] (...)

A: (...) a cobra em si ela não morde as pessoas (...)

OF1: (...) não, não (...)

A: o feiticeiro que manda a cobra morder?

OF1: o feiticeiro que manda (...)

A: e quem que manda o feiticeiro mandar cobra morder?

OF1: ah, ninguém sabe só eles que (...)

A: (...) só os espíritos que sabe.

OF1: é, só os espíritos que sabe.

A: E como que... como que você imagina que o outro feiticeiro manda fazer?

OF1: aí que outra pessoa que feitiço né feiticeiro esse daí sabe, aí que sabe que espírito chega aí que ele... ele contou, ele domina sim e falou e::.. outra pessoa agora já sei. Ele aguentou, eu te mandei uma coisa né assim falou né daí. Cadê, cadê agora, agora ele não aguentou, ele provou né assim. Se ele provou daí nem vale pra mim outro dia já morreu (...)

A: (...) morreu

OF1: morreu, morreu. (...)

A: então feiticeiro (...)

OF1: feiticeiro mata esse daí (...)

A: (...) o feiticei... (...)

OF1: o feiticeiro manda esse daí... não morre, não morre que não::.. não é rapidinho entra né o coisa... veneno se aquele:: se cobra... cobra mesmo assim, se não:: não (manda) [00:29:17 o feiticeiro esse daí pode chegar na hospital e tomou remédio para aconteceu nada chega em casa e nem me fazer nenhum nada, nem dor, nem outra coisa, nem sente, nem um nada por causa:: por esse ...feiticeiro manda... rapidinho.

A: morre rápido (...)

OF1: (...) morre rápido.

A: mesmo que levar pro hospital?

OF1: (...) mesmo onde tá esse (inint) [00:29:43] aí que. Nem levantar, nem corre (inint) [00:29:50]. Tu chega... tu chega longe porém assim lá no centro. Por aí 2 quilômetro. Aí que morde cobra, num... num... num... dá pra correr não.

A: não dá tempo de chegar na comunidade?

OF1: não dá tempo não na comunidade. Esse daí. Se aquele::.. se cobra né? Tu já entendeu, né? Se... se cobra:: cobra mesmo não mandar [00:30:12] no feiticeiro. Esse daí até chegar para o hospital. Parou:: quando tomou o remédio para esse daí do feiticeiro (...)

A: (...) o feiticeiro tomando remédio não para? (...)

OF1: Se... se... se.. se tomou aquele remédio que mandou feiticeiro esse daí (inint) [00:30:37]. Dor... dor... aonde da morde aí mesmo dor, aí mesmo dor esse daí.
A: entendi.

OF1: aí mesmo dor. Aí tu não aguenta só uma vez incha teu perna, mas não pode andar esse daí sim dor do coisa do feiticeiro.

A: então quando o feiticeiro manda a cobra morder a::.. o veneno é mais forte?

OF1: veneno é forte porque junto com o tabaco.

A: entendi. O::.. o... Rui então o::.. feiticeiro ele conversa com a cobra e a cobra obedece? O quê que ele tem que fazer? Me conta como é. (...)

OF1: (...) o espírito dele espírito:: se tu pensa daí, pensa mau com a outra pessoa né. Se já... já ele anda porém assim o de dia de qualquer hora né desse::.. esse feiticeiro né. Esse daí que de dia ou de noite qualquer hora se...se na casa, seu porta se aconteceu::.. aí que morde. Um vez me aconteceu comigo (...)

A: (...) como? Me conta aí.

OF1: quando... quando... do::... do 30 ano né aí meu tio que já morreu esse daí que
é profissional do feiticeiro mesmo (...)

A: (...) teu tio era feiticeiro? (...)

OF1: (...) era feiticeiro mas esse daí já morreu.

A: já morreu.

OF1: aí que... ele tem uma... uma cerca bem ali...assim como do outro lado. Aí que entrei de tarde (inint) [00:32:21] é 3 horas assim... 3 hora. Passei lá com ele numa rua né daí caminho...passei bem ai do lado dele... aí que eu entrei mais nem sabe aí tem uma coisa assim uma...uma tabua né pa passar o coisa. Aí eu passei bem aqui ó. Eu entrei aqui... pisou quase que em cima dele. Deste tamanhozinho, mas esse daí o (feiticeiro) [00:32:56] (...)

A: (...) era uma cobra?

OF1: uma cobra pouquinho assim. Desse tamanhozinho, assim como daqui. Deste tamanhozinho. Aí quase que me mordeu meu pé.

A: e era casa do teu tio?

OF1: esse casa::.. porta do meu... meu tio. Aí que eu olhei pro assim ó (inint) [00:33:14] cobra... cobra aí mesmo mata. Terçado né, mata logo. Assim que se:: tem que, tem que, tem que:: gente boa (...)

A: (...) tem que ser gente boa?

OF1: gente boa, mas outra pessoa não... assim que a feiticeiro faz.

A: Na comunidade vocês utilizam quando vão caçar, pescar, alguma:: algum material para proteger a perna ou alguma coisa assim?

OF1: é (...)

A: (...) vocês usariam ou vocês usam? Como é que vocês vão caçar?

OF1: é... quando nós estamo andando, né. Agora nós já sabe, nós já sabemo né. Nós levamos aquele... aquele (inint) [00:34:12] do coisa. Meu (genro) [00:34:14] já mandei ele tá ele quer:; gosta de caçar né. Sozinho andando lá lá no (inint) [00:34:23] lá...lá no outro lado coisa onde tem...tem animal. Ele leva remédio aquele daqui tem né, que tem. Anador e dipirona e paracetamol. E leva também até aquele feu do paca coloca no:: no coisa:: aquele vazio né do...do remédio (...)

A: (...) no vidrinho.

OF1: no vidrinho. Assim que falou pra ele todo tempo, mas não aconteceu nada a ele porque eu que cuida ele o caminho dele aonde tá o:: trabalhando, aonde tá o caçar, ele tão (gostando) [00:35:13] de longe né daí lá no capacete (nome da comunidade).

A: aí o senhor como curador, o senhor protege aquelas pessoas da sua família? (...)

OF1: (...) protejo da pessoa. É. É.

A: E que tipo de proteção?

OF1: proteção pra não acontecer algum::.. algum mal, algum que:: pisou o cobra, algum que... que acontece por aí. Oh tem onça, tem o bicho.. ninguém vê... esse daí pra afastar.

A: entendi. E o que é que o senhor faz pra essas pessoas ficarem protegidas?

OF1: apenas fazer oração.

A: uma oração.

OF1: uma oração.

A: aí o senhor uma::.. conta um pouco melhor sobre isso.

OF1: até por aí assim proteger e ele passou pa cuidar assim. Fazer tirar o coisa, tirar fazer... fez um com tabaco né daí, o tabaco e tira o capa dele e coloca com o capa dele com fumaça do::.. tabaco daí tira. aí depois eu assopra com coisa né no...do tabaco. abre caminho não acontece nada só o vê só ele quer.
A: entendi. É uma proteção que ninguém vê fica só (...)

OF1: (...) proteção. Só.. só ele que anda.

A: E quem que ensinou o senhor fazer essas orações, essas proteções?

OF1: (...) esse daí::.. Deus que...que me ensinou. Esse daí ninguém sabe pra ensinou.. só na minha pessoa.

A: só o senhor mesmo que recebeu?

OF1: recebeu esse daí.

A: e com quanto... com que sinais o senhor recebeu para saber que tinha esse dom de curar e de proteger as pessoas?

OF1: Esse senhor esse daqui ninguém me... me doou ele ensinou que eu saber Pai nosso né sete vezes Pai Nosso e uma Ave Maria e um credo esse daqui também trabalhando todo tempo.

A: entendi. São varias orações que o senhor faz no ritual (...)

OF1: (...) muito muito oração (...)

A: (...) muitas orações .

OF1: muitas orações. Isso daí tudo pra curar.

A: aí (...)

OF1: (...) junto com esse daí (...)

A: (...) aham

OF1: Se alguma coisa de outra pessoa te invejou... aí que eu... eu sabe tudo tem que pra curar...pra tirar o mal eu faço.

A: aí usa as orações que o senhor sabe mais o tabaco (...)

OF1: (...) orações mais o tabaco. Sá isso daí.

A: aí o senhor já faz uma defumação. Eu preciso (...)

OF1: (...) aí depois (...)

A: (...) pode falar.

OF1: E depois eu tenho:: trouxe uma remédio pa curar.

A: pra curar.

OF1: Essa aqui. Esse daqui é santo remédio.

A: esses remédios são pra::..serve para quê? Pra que tratamento.

OF1: (...) serve pra::.. esse é (inint) [00:38:45] tabaco pra tirar mal. Esse que... que é casa do...do jandaíra.

A: a casa da abelha jandaíra.

OF1: jandaíra. Esse aqui é breu branco...cheirosa. (...)

A: breu branco pra defumar (...)

OF1: (...) defumar. junto com esse daí pra tirar mal você tem. Pra tirar aquele agora tem... tem doença nesse tem agora:: tem gripe tem outra coisa de mal.

A: uhum.

OF1: Tem alguma pessoa que tão... tão maldade pra o família tira pouquinho assim só esse agora tá... tá quebrado já tá bom. tira pouquinho assim, tira com...com...com fogo, coloca no fogo pouquinho assim, deixa na casa três vezes, casa assim, quarto... quarto (...)

A: (...) defumando?

OF1: defumando, defumando todo canto. A perna, tua:... lado de casa por aí 3 vezes. Parou. Parou de maldade.

A: E com quantas vezes é::.. eu devo fazer isso?

OF1: esse daí. Porém três vezes tu faz isso. Defumando assim pra cá já... já tá resolvido.

A: três vezes?

OF1: três vezes.

A: e durante um ano quantas vezes eu devo fazer? (...)

OF1: (...) três vezes por aí. Se você::.. se acontece alguma coisa, né. Aí tu faz. Se tu... se tu não acontecer nada:: não.

A: não precisa fazer?

OF1: não precisa fazer, não precisa fazer.

A: então só precisa renovar quando acontece alguma coisa? (...)

OF1: (...) só renova. É.

A: E... e essas defumações também protege das cobras também?(...)

OF1: (...) protege tudo... tudo.

A: tudo de mal?

OF1: você::.. maldade. nada porém sozinho, né. Esse daqui é bom.

A: Deixa eu lhe perguntar uma coisa se eu sou picado por uma cobra que alguém mandou picar pra mim picar eu posso ir com o feiticeiro e mandar ele picar a pessoa de volta?

OF1: não.

A: não pode

OF1: não. Que é esse daí já... já termina onde termina já tá no seu último já.

A: entendi.

OF1: preço de de:: esse daí te mata né, daí te mata. Por isso que tá feiticeiro mandado pra te matar esse daí pra ele não tem coragem pra ele mesmo mata né. Aí na vida dele oração que queima assim.

A: entendi.

OF1: assim que a pessoa que é aquele espírito que é mal dele, né? Que daí que faz

A: e na comunidade tem outros feiticeiros lá?

OF1: tem, tem

A: tem muitos feiticeiros?

OF1: uh:: tem muito, tem 72 feiticeiros.

A: 72 feiticeiros?

OF1: 72 feiticeiros.

A: E:: quem me procura essas pessoas feiticeiras?

OF1: ai, ai, ai, ninguém sabe.

A: ninguém sabe

OF1: ninguém sabe.

A: as pessoas procuram escondido o feiticeiro? (...)

OF1: (...) feiticeiro é escondido.

A: é escondido.

OF1: Mas tu vai lá:: pergunta ninguém sabe. Tu sabe como que é aí tu sabe toma um pouquinho de coisa ayahuasca? Já tomei (...)

A: (...) já tomei ayahuasca.

OF1: Ai que essa pessoa::: aí tu vê (...)

A: (...) ah se eu for lá na comunidade eu tomar ayahuasca eu vou saber qual é o feiticeiro? (...)

OF1: (...) vai saber qual o feiticeiro lá aí que eu (inint) [00:42:45] ah ante eu não sabia quanto tem o bicho lá tem. Agora já sei, mas não tem medo. Aí ele tem sempre ele merece pra me matar termina logo esse daí que eu tô mentindo assim me falou eu tô mentindo. Pra quê que esse rapaz:: ele não sabe nada. Pra mim ter muita gente que tem eu curei:: eu cura (...)

A: (...) o feiticeiro não gosta do trabalho que tu faz? (...)

OF1: (...) meu trabalho, meu trabalho que é bom. Quando:: quando a criança né, primeira criança, criança que assim deste tamanhozinho esse daí se ela:: chorando, gritando chora esse daí que é cura. Esse daqui tá faltando coisa o pai dele não tá bem, não... não cuida bem e ele anda por é assim a vida dele levar por aí assim:: deixado por aí assim:: a vida dele:: aquele menino. Esse daí tá chorando, gritando todo noite de dia nunca dorme esse menino. Esse daí muito...eu to curando.

A: só atende principalmente criança?

OF1: criança (...)

A: na comunidade.

OF1: na comunidade. Agora me chama todo tempo:: de noite, eu nunca dormi eu. (inint) [00:44:16] de noite até 3 horas por aí qualquer hora me chama pra curar. Agora aí tira, tira isso daí aquele mal pra ele. Tem outra pessoa tem:: entra a:: não é bom pra levar espírito (inint) [00:44:38 esse daí grita aí só nunca pegou (...)
A: (...) não consegue dormir?

OF1: não consegue dormir. até pra morrer (três dias assim) [00:44:46] esse já tá morrendo (...)

A: (...) se não procura

OF1: Se não procura comigo.

A: aham.

OF1: morre.

A: morre.

OF1: morre

A: então quando for assim tem que procurar logo o curador (...)

OF1: tem que procurar logo o curador. aí para:: para de chorar, conseguir dormir assim. Aí depois eu que faz as coisas, faz a capa dele, capa dela sim se tem (moleira) [00:45:21] tira com o tabaco né daí. (inint) [00:45:24] dobra bem assim

A: é uma capa de proteção (...)

OF1: (...) capa de proteção. Até pra, até pra durante quantos anos (...)

A: (...) por muito tempo

OF1: muito tempo assim.

A: entendi.

OF1: aí, eu faz pra quando a menina se... se... se... se nós também assim como adulto, né? Agora tô::.. mexendo com adulto agora trabalhando com adulto já. Aí muita mamãe que não cuida bem porém três vezes. o cai na água

[00:46:06]

Entrevista Cuidador 2

Idade: 47 anos

Religião: Ordem da Santa Cruz (Cruzada)

Informações:

a. Se define como curador/rezador, possui o dom da reza para curar a dor, é especialista no atendimento de crianças indígenas, principalmente nos casos de vômitos e diarréias.

b. Destacou que o tabaco é um elemento importante na prática de cura, porque faz uma espécie de proteção. Também utiliza remédios caseiros, principalmente os travosos e amargos.

c. Em relação ao tratamento de acidentes ofídicos, destacou que ocorrem mais quando o rio está cheio e que utiliza língua de pirarurucu, saratudo e casca de taperebá.

[00:00:02]

A: Vamos lá. Boa tarde seu Ivonei.

OF2: Boa tarde.

A: Nós vamos começar aqui, eu gostaria que seu relatasse para mim bem detalhadamente é... quando, o que:: se ocorre, o senhor já falou que ocorre acidente com cobras e:: os casos que o senhor já viu, queria que relatasse como que aconteceu.

OF2: Sim. Ah:: no início a gente é picado pelo uma cobra a... depois da picada dá cobra a primeira coisa que... às vezes a gente faz lá na comunidade é amarrar ou então com pano ou cipó. O que tiver na hora pra pôr o efeito do sangue da...da cobra do veneno da cobra não espalhar pelos corpo, pelos sangue aí a gente amarra com pano, com pedaços de cipó... que seja...na hora:: pra chegar até o local mais... mais próximo da comunidade.

A: Onde o que acontece essas picadas de cobra? As pessoas estão fazendo o quê?

OF2: Elas estão caçando, tão fazendo roça, aí acontece mais isso.

A: Eles não tão na comunidade (...)

OF2: (...) estão distante da comunidade. Aham. Estão distante da comunidade.

A: Em relação aos cipós que o senhor falou é um cipó especifico ou qualquer cipó?

OF2: Não. Qualquer cipó. Pega (...)

A: (...) pega pra amarra.

OF2: pega pra amarrar.

A: que é pro veneno não se espalhar.

OF2: que é pro veneno não se espalhar.

A: e aí vocês trazem pra comunidade?

OF2: pra comunidade.

A: e na comunidade vocês fazem o quê?

OF2: a gente chega entrega po agente de saúde (...)

A: (...) pro agente de saúde.

OF2: pro agente de saúde. Aí agente saúde se encarrega por ele.

A: entendi. E aí ele é transferido pra... pra tomar (...)

OF2: (...) é. Ele é transferido (...)

A: (...) pra cidade.

OF2: pra cidade.

A: entendi. E além desse::... além desse... durante esse... essa... essa distância que vocês estão caçando, pescando. É... o que mais que vocês fazem além disso? E por que que é feito isso? Tipo utiliza alguma planta, utiliza alguma coisa?

OF2: Não que na hora a gente não utiliza negócio de planta na hora a gente vai utilizar já depois da... quando o retorna do hospital que a gente toma outro remédio, né que a gente arruma pa fazer uma massagem, fazer uma compressa em cima.

A: e o quê que utiliza pra fazer a compressa?

OF2: folha de:: língua de pirarucu é uma planta comprida assim, deste tamanho assim a gente usa.

A: e que efeito que essa planta faz? Que benefício que ela faz?

OF2: É... a gente... a gente soca ela, tira só aquele (sumo) [00:02:39] verde a gente bota em cima. Aí ela sara rápido a...a ferida da cobra né. Onde ela picou.

A: Tá. Então vocês... vocês quando são acidentados, vocês (tão) [00:02:51] os primeiros socorros é amarrar, trazer pra comunidade, pedir ajuda do... dos agente de saúde, traz para tomar um soro na cidade, na volta que vocês vão fazer o tratamento caseiro (...)

OF2: (...) é. O tratamento caseiro já.

A: E aí o tratamento caseiro é com::... com a (...)

OF2: (...) planta. Língua de pirarucu (...)

A: (...) língua de pirarucu. E o que mais? Que... que outras e... medicinas caseiras vocês fazem?

OF2: Que... são várias né... são várias.

A: Pode falar tudo detalhadamente.

OF2: (inint) [00:03:28] Por que tem a língua de pirarucu:: a língua de pirarucu. Tem o:: o:: sara tudo que chama sara tudo lá... sara tudo. Tem a:: a:: casca de taperebá... casca de taperebá e::...

A: Essas... essas três que você falou, a língua de pirarucu vocês maceram (...)
OF2: (...) é.

A: e colocam espreme o sumo pra colocar em cima (...)

OF2: (...) o sumo pra colocar em cima.

A: o sara tudo e a casca do taperebá. Como que são preparados? Como que são utilizados?

OF2: A gente só tira o sumo também, pinga o sumo em cima também.

A: Também. Tudo é (...)

OF2: (...) é que ela é (travosa) [00:04:14] aí ela, ela sara.

A: ela ajuda (...)

OF2: (...) ajuda a sarar o ferimento (...)

A: (...) a sarar o ferimento. Entendi. E... você me relatou que duas vezes é... o senhor lembra bem de dois casos de acidente por picada de cobra é isso? Foram homens, mulheres, crianças o quê que foi? Como que foram nesses acidentes? Lembra detalhe deles?

OF2: não. Eu::... fui saber de quando já chegaram na comunidade já né? Aí os detalhes não sei muito bem... os detalhes aí.

A: e o quê que eles contaram baseado no que foi falado?

OF2: Eles...eles...eles foram pra roça:: eles tão...tão capinando a roça.

A: e que cobra que foi? Normalmente que cobra que pica na comunidade?
OF2: Jararaca.

A: jararaca é a principal.

OF2: jararaca, jara... jararaca é a principal.

A: E quando a jararaca pica que sintoma que o paciente fica?

OF2: dá uma tontura.

A: dá uma tontura?

OF2: dá uma tontura, dá uma tontura. A... o paciente pode até desmaiar dentro de uma hora...

A: de uma hora o paciente pode desmaiar. Entendi.

OF2: Por isso que a gente tem...tem que antes quando ela pica a gente tem que amarrar logo pro veneno não se se espalha por dentro.

A: E corre para comunidade para pedir ajuda (...)

OF2: (...) Pra comunidade pedir ajuda.

A: entendi. É...Por que que acontece esses acidentes? Como que cê compreende isso? Por que acontece a picada de cobra?

OF2: porque a picada da cobra estão no (cerrado) [00:05:50] né. A gente vai pra roça, a gente vai roçar, aí a gente não enxerga se ela tá lá ou não. E quando a gente trisca nela ela ataca.

A: entendi.
OF2: E aí a gente não tá esperando se vai acontecer ou não.

A: Tem alguma época do ano que tem mais picada de cobra? como é que (inint) [00:06:09](...)

OF2: (...) mais quando tá cheio.

A: quando o rio tá cheio (...)

OF2 (...) tá cheio, quando o rio tá cheio.

A: Quem vai pescar também corre o risco de ser ? (...)

OF2: (...) Às vezes acontece, as vezes acontece.

A: É. A mesma jararaca?

OF2: A mesma jararaca. É o que mais tem na região a mesma jararaca.

A: a maioria jararaca.

OF2: a maioria jararaca.

A: e crianças também são picadas por cobra?

OF2: as vezes né.

A: é? Em que situações?

OF2: Às vezes tomando banho, né... as vezes tomando banho acontece na água.

A: na beira do rio?

OF2: na beira do rio.

A: do rio ou do igarapé ? o que vocês (inint) [00:06:42]

OF2: do igarapé.

A: Quando uma pessoa na comunidade encontra uma cobra o que deve ser feito?

OF2: na maioria encontra uma cobra lá a gente mata.

A: É?

OF2: a gente mata. A gente mata as cobras. Principalmente as venenosas né... as venenosas.

A: e vocês sabem diferenciar as cobras venenosas? o senhor sabe?

OF2: devido.... devido da cabeça, né pelo formato da cabeça.

A: reconhece pelo formato da cabeça?

OF2: é, pelo formato da cabeça.

A: entendi. E quando um acidente como o senhor já mencionou aí que alguns aconteceram na sua comunidade é::... quando acidente com cobra acontece o quê::... como é que acontece esse processo? O quê que o indígena vai sentindo? vocês vão observando.

OF2: é aquela dor, uma dor né.

A: e onde é essa dor?

OF2: aquela dor que vem desde a da picada a gente sente aquela dor que ela vai subir a dor vai subindo.

A: descreva melhor aí pra mim como é que o paciente fica depois que é picado?

OF2: O paciente sente aquela dor não consegue se::... locomover, (as vez fica aquele paralitico sabe) [00:07:57] não consegue andar, dá aquela tontura cai no chão,

A: em geral eles caem no chão.

OF2: em geral eles caem no chão. aí tem que levar mais rápido para socorrer.

A: entendi. E durante esse processo são feitos alguns tratamentos como o senhor já citou? Durante o::... Quando vocês é... são picados por cobra, quando nas aldeias é::... importante matar a cobra ou vocês se afastaram dela?

OF2: não, na hora as vez a gente mata, né. Na hora né. A gente procura quando a gente encontra a gente mata, quando a gente não encontra a gente deixa ela ir. Que é mais é pra... pra reconhecer qual foi o tipo da cobra, né.

A: pra identificar.

OF2: pra identificar

A: Para falar na cidade (...)

OF2: (...) pra falar na cidade qual foi o tipo de cobra que para tomar o soro certo (...)

A: (...) pra tomar o soro certo.

A: tá. E aí durante esse processo de vem para cá para cidade, né?

OF2: sim.

A: Aí ele depois que ele pega alta de volta para comunidade.

OF2: volta pra comunidade.

A: E aí lá são feitos...feito alguns tratamentos (...)

OF2: (...) tratamentos, é.

A: E aí os tratamentos pelo senhor descreveu aqui é... até agora só o tratamento com a base de planta (...)

OF2: (...) plantas.

A: tem outros tratamentos que envolve outras coisas que não sejam plantas também?

OF2: não, não só remédio de planta mesmo (...)

A: (...) remédio de planta.

OF2: de planta.

A: entendi. Pra vocês a comunidade é importante trazer o paciente imediatamente para o hospital?

OF2: é.

A: por quê?

OF2: porque que na hora a pessoa pode morrer também na hora. Pode morrer na hora.

A: E o que o que o indígena precisa saber ou fazer para ele não ser picado? Como que pode evitar esses...esses acidentes?

OF2: as vezes a gente usa bota, mas mesmo assim bota ela.. o dente da cobra ela transpassa a bota. Ela fura a bota.

A: é?

OF2: é.

A: o que mais que vocês usam (...)

OF2(...) é só isso mesmo (...)

A: (...) pra se proteger?

OF2: a bota.

A: a bota. Então é um::... um equipamento de proteção física.

OF2: proteção física.

A: e os acidentes quando acontece lá na comunidade mais em que parte do corpo?

OF2: é mais a parte da perna.

A: da perna.

OF2: mais a parte da perna.

A: então é por isso que é importante usar bota?

OF2: usar bota. Na hora de ir pra roça, na hora de caçar.

A: os ticuna usariam as botas sem problema nenhum?

OF2: Sem problema nenhum (...)

A: (...) ou eles teriam alguma resistência para ir para caçar?

OF2: (inint) [00:10:53] de bota. Mais protegido de bota.

A: de bota. Eles têm essa consciência de que é importante usar bota?

OF2: usar bota.

A: tá. Existe alguma outra forma de proteger o corpo de picada de cobras que não seja fisicamente que seja utilizando algum amuleto usando algum::... algum::... alguma recomendação do... do pajé alguma coisa assim importante?

OF2: não. aí a... a nossa recomendação que nós diz... é se proteger, né. Se cuidar da cobra. Olhar bem a prestar atenção na hora de sair, de andar na mata,

A: é mais a questão de atenção.

OF2: de atenção, de atenção mesmo.

A: entendi. Ah, uma outra coisa aqui. É::... o que situações que o acidente da picada de cobra pode agravar ou pode ficar mais grave, pode piorar o que situação isso?

OF2: às vezes quando ela fica inflamado.

A: mais o quê? Em que situações quando vai ficar inflamado, mas provocado por que essa inflamação?
OF2: as vezes quando... ela::... ela demora muito... demora muito a ser socorrido.

A: quando demora a ser socorrido.

OF2: a ser socorrido. aí aquele veneno apodrece a:... o sangue por dentro aí vai (...)

A: se demorar pra ser atendido ele poder ser (...)

OF2: (...)(inint) [00:12:36] ser atendido.

A: Existe alguma outra coisa que que pode agravar em relação a dieta por exemplo o paciente pode comer tudo? Ele pode (...)

OF2: (...) não. Tem que fazer a dieta.

A: então me explica aí um pouco como é que é uma dieta de um paciente? O quê que é remoso pra ele? (...)

OF2: (...) (inint) [00:12:57]. O que é remoso pra ele é piranha, bodó, pacú, mandií, surubim, pirabutão, todos os tipos de peixe são remoso. O que não é:: o que eles podem comer na hora curimatã, traíra, sardinha esses três peixes (...)

A: (...) esses três peixes devem (...)

OF2: (...) deve ser consumido na hora da dieta dele.

A: E por quanto tempo? Ele tem que ficar (...)

OF2: durante... durante uma semana... uma semana ou duas dependendo da (...)

A: (...) da evolução.

OF2: da evolução dele.

A: ele tem que ficar com um determinado resguardo?

OF2: determinado resguardo.

A: e cumprir essa dieta?

OF2: e cumprir essa dieta.

A: e::... e::... em relação aos remosos. Porque que ele é remoso? porque que ele é remoso para ele?

OF2: é que inflama. Um exemplo: a piranha o dente dela são afiados

A: uhum.

OF2: Aí com isso aí ela vai::... ela inflama a ferida e ela vai comendo na carne por dentro, isso aí ela vai inflamando a carne por dentro do ferimento. Aí inflama, aí incha a perna, aí daí pode acontecer outro tipo de (...)

A: (...) complicação.

OF2: complicação

A: e os outros peixes?

OF2: os outros peixes são do mesmo jeito (inint) [00:14:13] (...)

A: (...) peixe liso por exemplo.

OF2: devido o esporão. Ele tem uma espora a gente come isso aí... aí a gente sente aquela dor na... na... ferida parece que tá furando, aí vai inflamando mesmo jeito também.

A: então tá relacionado com característica (...)

OF2: (...) isso, característica de cada tipo de peixe.

A: cada tipo de peixe (...)

OF2: (...) cada tipo de peixe.

A: e outra:: e outros alimentos que pode comer?

OF2: pode. (inint)[00:14:38 pode comer.

A: Quais alimentos que pode comer além desses?

OF2: além::... além do peixe?

A: é.

OF2: além do peixe... pode comer arroz, arroz ele pode comer arroz normal... arroz.

A: e outros animais?

OF2: frango.

A: o que é remoso também? Comer porco, comer galinha, pato comer (...)

OF2: (agora) [00:14:57] porco é remoso também, comer porco é remoso também. Agora galinha não é remoso não galinha.

A: durante esse resguardo que o paciente com... picada de cobra deve cumprir que outras regras sociais, familiares ele também deve cumprir? tem alguma coisa eu pode agravar?

OF2: não é, só isso mesmo.

A: é?

OF2: é só isso mesmo.

A: então ele pode viver com a mulher dele normal?

OF2: é pode viver com a mulher dele normal.

A: não faz não agrava nem piora nem melhora?

OF2: nem piora, nem melhora.

A: Existe algum::... alguma orientação na comunidade que é feito pelos pajés, pelos agentes de saúde para se proteger e evitar esses... esses acidentes.

OF2: às vezes existe só que a gente tem que ir né a busca daquele alimento, vão caçar vão pescar, vão para roça, a gente tem que ir se a gente não ir para roça quem que vai sustentar a família da gente? aí a gente tem que ir::... aí tem que ir

se arriscar.

A: entendi. O senhor já viu alguma criança picada por cobra?

OF2: não. Não. Criança não, criança não.

A: É mais adulto (...)

OF2: mais adulto (...)

A: que são aquelas que se afastam mais da comunidade?

OF2: (...) mais adulto.

A: (inint) [00:16:51] vocês tiram madeira da comunidade?

OF2: não.

A: não tira. é mais na plantação.

OF2: mais na plantação.

A: de... (...)

OF2: (...) mandioca

A: mandioca.

OF2: banana, abacaxi.

A: as mulheres também nessas atividades agrícolas elas também são picadas por cobra?

OF2: as vezes são picadas, as vezes.

A: e o quê que as mulheres fazem para se proteger?

OF2: é... é... é... do mermo jeito que... que o homem faz também né? usa bota. Quando tem né? Bota. Quando não tem.

A: Existe alguma forma de proteção de fechar o corpo uma coisa mais espiritual, o senhor acha que funciona ou se tem alguma prática nesse sentido no... na comunidade indígena?

OF2: a gente não::... pra cobra não.

A: existe para outras coisas?

OF2: outras coisas.

A: pra que coisas por exemplo?

OF2: por exemplo: pra feitiço ~~pedir~~... para se proteger de:: de doença. Aí a gente fecha o corpo

A: como que é feito esse fechamento de corpo?

OF2: a gente reza, a gente assopra.

A: o quê que se utiliza nessas rezas esses assopros?

OF2: a gente... as vezes a gente usa a defumação.

A: é?

OF2: é, a gente (...)

A: (...) descreve pra mim uma...um o processo de defumação.

OF2: é com alho, com alho, com.. casa de caba, casa de abelha seca... seca, quando tão seco já e... alho aquelas folhas de alho que são grande já uma planta a gente pega numa panela e joga dentro da panela e queima ele ficar disfumando só, aí a gente começa... aí a gente reza.

A: E aí vocês.. e vocês fazem uma defumação pro ambiente ou pra aquela pessoa que tá naquele ambiente receber ? (...)

OF2: (...) pra aquela pessoa que tá naquele ambiente.

A:E aí o que que vai acontecer com o corpo daquela pessoa?

OF2: ele vai ser protegido.

A: é.

OF2: ele vai ser protegido.

A: de todas as doenças?

OF2: todas as doenças.

A: E esse processo ele é::... tem data pra.. pra tem uma validade? Por quanto tempo o indígena precisa fazer esse processo pra tá protegendo todo corpo?

OF2: A gente tem que fazer quase toda semana as vezes... quase toda semana.

A: tem que estar renovando

OF2: (...) tem que tá renovando é. Ela não tem aquele prazo longo não. A gente tem que sempre tá fazendo aquela proteção ali.

A: entendi. É... entre os indígenas da sua comunidade é... é perigoso uma mulher gestante ver é... uma... uma pessoa que foi picada de cobra ou não faz diferença?

OF2: não faz diferença não.
A: não?

OF2: não faz diferença não.

A: Tem alguma... mais alguma coisa em relação à picada de cobra que o senhor gostaria de contar que aconteceu na comunidade que o senhor já ouviu falar... algum caso... importante? não precisa ser exatamente na sua comunidade, mas que o senhor acha curioso e gostaria de contar.

OF2: eu...eu... eu já ouvi meu...meu avô contando.

A: (conta) [00:20:29]

OF2: a surucucu.

A: sobre a surucucu?

OF2: surucucu.

A: o quê que tem a surucucu?

OF2: ela...ela, ela picou minha tia.

A: o que... como é que foi feito esse atendimento?

OF2: aí esse aí eu não sei detalhadamente sei contar essa história não, mas o que meu avô falou que minha tia ficou com a perna paralítica, secou um lado da perna dela, demorou muito a ser socorrido.

A: Eu... onde ela foi picada?

OF2: foi picada na perna, na batata da perna. isso aqui secou tudinho e ela ficou dessa grossurinha.

A: e ela tava fazendo o quê?

OF2: ela tava na roça.

A: pois é, mas onde?

OF2: lá em Tonantins.

A: lá em Tonantins (...)

OF2: lá em Tonantins. Abaixo de Santo Antônio de içá.

A: e aí o quê... com quanto tempo depois levaram ela pra.. pro o hospital?

OF2: Depois de... depois de três dias já.

A: depois de três dias. Ela conseguiu escapar, mas sequelada da perna?

OF2: ficou sequelada da perna.

A: é. E quem que... o senhor reza nas nas crianças, nos adultos? Quem que ensinou o senhor fazer esse (procedimentos)?

OF2: meu avô.

A: como é que foi essa transferência de... de conhecimento? Fala um pouquinho aí da sua vida nesse sentido?

OF2: o... o meu avô me ensinou a...a rezar quando eu tinha o meus 12 anos::... 12 anos. Aí ele chegou comigo falou: meu filho, você quer aprender a rezar? Eu não sei você que sabe. Eu tenho um dom eu lhe vou lhe dar esse dom.
A: E aí como é que foi esse processo aí?

OF2: Aí ele me chamou e me rezou... ele me rezou. Rezou normal mesmo. Eu e ele sentado assim na frente e ele me rezou. Aí mais ou menos meia-hora: aí meu filho a partir de hoje você já tem o dom.

A: você o quê?

OF2: você já tem o dom já.

A: ah, já tem o dom para conduzir outros::...outras pessoas (...)

OF2: (...) pra conduzir outra pessoas, mas não agora daqui uns tempos. E esse dia vai chegar sem tu saber.

A: uhum. E aí? (...)

OF2: E aí ninguém esse dia chegou mermo (inint) [00:22:43]

A: (...) como é que foi, como é que foi?

OF2: Eu não tô muito lembra... que dia que foi que que aconteceu, mas teve.

A: mas com que idade que o senhor já estava

OF2: tá com... 18 anos, 18 anos mais ou menos já tá com 18, 19 anos. Aí aconteceu, eu tava em casa. Aí sem esperar acontece.

A: e como é que foi isso?

OF2: Aí eu tava na minha residência lá (...)

A: (...) pode contar

OF2: tava sentado na minha residência lá e tinha uma criança doente. Aí a criança tava doente lá, aí eu fui lá... aí cheguei com a mãe da criança e: a senhora acredita em mim? perguntei três vezes aí ela: a senhora acredita em mim? Se a senhora acreditar em mim eu deixo sua filha bonzinho. Aí ela: eu acredito, mas o senhor sabe rezar? Ela perguntou de mim. Aí eu disse: não, mas eu vou deixar ela bonzinho agora.

A: E aí que foi feito?

OF2: Eu rezei, rezei na hora mais ou menos meia-hora depois de um dia. Teve um resultado.

A: e por que que a criança tinha? E como que ela ficou depois?

OF2: a criança ela tava... ela tava espantada a criança, ela tava magrinho.

A: o que que eu a criança espantada?

OF2: ela... ela fica com diarreia... diarreia e vômito, aí ela emagrece e na hora de dormir, ela não consegue dormir só veve se assustando. Aí isso vai enfraquecendo a criança::... vai enfraquecendo a criança.

A: aí o senhor rezou quantas vezes?

OF2: rezei três vez. Rezei três vez.

A: Pra gente finalizar... retomando a questão do acidente ofídico, da picada de cobra. Cite para mim algumas coisas que... a gente nunca deve fazer com paciente que foi picado por cobra?

OF2: que não deve fazer?

A: é.

OF2: Na hora da picada?

A: é para não piorar.

OF2: pra não piorar né. é... é não comer as coisas remoso, né? Que é para não piorar. As coisa reimoso. É só isso mesmo.

A: Só cumprir a dieta?

OF2: é só cumprir a dieta... a dieta.

A: destaque as coisas que foram mais importantes para ele fazer, para melhorar e ficar sem sequela.

OF2: é...é::... passar os remédios que tem para passar. Os remédios que tem pra passar... remédio caseiro::... Aí ela não fica com sequela.

A: os remédios caseiros (...)

OF2: (...) é os remédios caseiros (...)

A: com as plantas que já indicou?

OF2: é.

A: o que que tem nessas plantas que é o tipo que melhora? Nesses casos.

OF2: é o::.. é o sumo dela, que ela é forte, ela são travoso, amarga, isso aí..com esse aí ela vai sarando, ela corta o efeito da picada... da picada de cobra, do veneno da cobra. Depois que toma o soro de cobra, aí ela (...)

A: (...) ela ajuda a manter junto com o soro (...)

OF2: ajuda junto com o soro.

A: é... o senhor destacou em relação das plantas, em plantas e amargas. Isso é uma característica importante para uma planta se utilizado como tratamento da travosa e amarga?

OF2: é. Ela... ela sara o local.

A: o que... que o travoso e amargo ajuda? Contém o que eu o senhor acredita que contém nessas plantas?

OF2: (inint) [00:27:12] com isso aí ela calma dor.

A: ela acama a dor.

OF2: acalma a dor. Aí por isso que a gente passa essas plantas aí em cima.

A: e elas também podem ser ingeridas as plantas?

OF2: não é só...só colocar no local mesmo.

A: essa pode ser... (...)

OF2: no local mesmo.

A: Existe algum procedimento que faça com próprio:: com a própria... com a própria... com a própria cobra algum procedimento que o senhor conheça ou já ouviu falar?

OF2: não, não. Conheço não.

A: Então:: então é isso, eu acho que a gente já... uma... uma cobra em si pra... pra comunidade ticuna, é:: ela tem alguma:: alguma representação específica é:: de adoração, de temor, de medo como que em geral os indígenas da comunidade observam?

OF2: não que a maioria da... já tão acostumada a vez com isso já né. já tão acostumado com isso. às vezes quase não tem medo de cobra. Quando acontece o acidente e por acidente mesmo da cobra né.

A: por que pisou em cima (...)

OF2: (...) pisou em cima ou então mexeu. Que a cobra ela só pica quando a gente mexe, a gente toca nela aí ela... (...)

A: (...) ela reage.

OF2: ela reage. Se a gente não toca, a gente passa perto, né?

A: Então se vocês veem uma cobra na comunidade, na roça. O que que vocês fazem?

OF2: a gente deixa lá mesmo, né? Se ela não ataca e a gente deixa ela mesmo.

A: entendi.

OF2: deixa ela lá mesmo. A gente não mexe.

A: ela segue lá...

OF2: ela segue

A: Em que ambientes que existem mais essas.. essas cobras? em que tipo de ambiente?

OF2: mais é na mata mesmo.

A: na mata?

OF2: na mata mesmo. A gente vai roçar, mais é na mata mesmo.

A: entendi. Então é isso, acho que...

[00:29:22]

Entrevista Cuidador 3

Idade: 78 anos
Religião: Ordem da Santa Cruz (Cruzada)

Informações:

a. Destacou que os acidentes ofídicos ocorrem geralmente durante a caça, pesca e nas roças ou no caminho de acesso pela manhã ou final da tarde.
b. Utiliza a folha de cubiu no tratamento de picada de cobra e que já tratou com sucesso não somente humanos, mas, um cachorro durante a caça.

[00:00:06]

A: Seu Felisberto, bom dia.

OF3: Bom dia.

A: Me conte aí um pouco é.. da... da sua história como que o senhor começou a fazer tratamentos, curas essas práticas quem que o senhor aprendeu isso?

OF3: é:: naturalmente eu aprendi fazer remédio assim pra... picada de cobra, né? E pra picada de arraia e... também pra:: a... picada pra... ferrada de tucandeira. (...)

A: (...) tucandeira.

OF3: isso. É...é... o remédio que ninguém conhece aqui. Como essa folha aqui.

A: como é o nome dessa folha?

OF3: é... cubiu.

A: cubiu.

OF3: folha de cubiu.

A: e o que... que... que a gente faz dessa folha?

OF3: a gente... por exemplo: a gente não conhece. A cobra que picou sujeito né e (nem) [00:01:21] identificado. Aí você pega isso::.. e tira três folhas. Aí você pisa ela bem pisado, tira o sumo... sumo , coa bem ne... dentro de um::... litro de água. Que dê uns três copos, num importa que ele já passou 4 dias, 5 dias já doente. Então ele vai tomar o suco disso aí, nós tempera com açúcar bem adocicado e depois você coloca óleo seja de comida ou seja óleo elétrico coloca

dentro, óleo de comida bem temperado com óleo. Aí tá preparado, aí você vai dar pro paciente que tá picado de cobra. Se ele tá pa morrer, mas se Deus permitir.. mas se toma isso aí, toma hora que chegar aí dentro de 6 em 6 horas. Aliás toma é.. 6 em 6 horas. Toma de manhã vamos dizer um copo, 12 horas toma outro, 6 horas toma outro, você fica observando se ele vai melhorar ou não. Então embora que já tomou soro antiofídico, antilaquetico, anti (inint) [00:03:12]. Tudo isso aí mas não importa. Nós retiramos com esse aqui...o rapaz foi buscar um no hospital de Benjamin tava bem inchado, não tinha como mais::.. o médico diz que tava suspenso...tava de alta porque não tinha como mais não aí.. aí eu fiz esse remédio. Uma hora dessa assim ele tava la em casa e quando foi 7 horas da noite, depois que ele tomou ele se aperreou pra lá. Puxando pra lá e pra cá (inint) [00:03:51] e o pai do rapaz foi buscar rancho dele lá dentro do igarapé Crajari ele não chegou cedo, chegou umas 8 horas da noite. Aí o rapaz calou, quietou aí eu disse para minha mulher: a gente vai ver como ele tá. Ele tá quietinho.

A: faziam quantos dias que ele tinha sido picado?

OF3: tava com cinco dias no hospital (...)

A: (...) no quinto dia no hospital aí ele saiu pra ir para casa?

OF3: lá pra casa onde eu estava.

A: aí ele tomou? (...)

OF3: (...) tomou... bem quando foi mais 8:00 o pai chegou aí vê ele tá lá, ele se aperreou muito, não sei se ele tá vivo ou tá morto. Aí ele foi ver lá, chamou aí ele respondeu o pai dele: senhor. Sou eu que tô chamando. Ah é o papai, ta bom. ~~meu pai Parou a dor Parou de tá botando mais nada de estar~~. Como é que cê tá se sentindo? bem? Ele disse: eu tô me sentindo bem. Parou a dor? Não tá doendo mais não. Parou de... de cuspir o sangue? Tô botando mais nada não. Então eu disse pra ele...eu não me lembro o nome do pai, aí eu disse daqui há 3 dias ele vai tá andando. Ele disse será irmão? Vai. E quando foi no outro dia de manhã, ele tava se desinchando, desinchando, desinchando, desinchou no terceiro dia ele já tá sentado. Ele disse assim: eu quero urinar mas eu vou urinar lá fora. Vai andar? (...)

A: (...) ele já tava andando?

OF3: andando. Desinchou tudo... tudo que tava ruim parou. O que tava a perna dele bem roxa que tava inchado ali (...)

A: (...) tinha sido a picada na perna?

OF3: é. Na perna.

A: e qual cobra tinha picado ele? O senhor sabe?

OF3: ninguém sabe que cobra era essa.

A: e onde ele tava? O quê que ele tava fazendo quando a cobra picou ele?

OF3: tava trabalhando lá na...na.. na roça dele.

A: na roça.

OF3: lá dentro... (...)

A: na terra firme?

OF3: na terra firme. Bem e aí quando:: três dia ele tava andando já devagar tá.. até o pai dele: olha meu filho tá andando graças a Deus e que tava toda roxa já, acho que já tava apodrecendo.

A: apodrecendo. Então depois do tratamento do sumo da folha do cubiu com óleo elétrico ou óleo de comida (...)

OF3: (...) elétrico ou óleo de comida (...)

A: mais (...)

OF3: (...) açúcar

A: o açúcar.

OF3: só essas três composição.

A: essas três composições melhorou. E o que que o senhor entende, o que que teria ~~teria~~ essa planta pra ajudar no tratamento desse?

OF3: olha, só... só através de Deus abençoe ele né e ele ficou bom e:: depois continuei fazendo pra alguém que tá querendo saber que fica bom e o senhor foi mordido de cobra jararaca no porto bem 5 horas da manhã.

A: outro caso?

OF3: outro caso.

A: Como foi esse caso aí? conta para mim?

OF3:Aí, o filho dele foi me avisar para morar assim distante (...)

A: (...) era um senhor adulto?

OF3: é adulto. Ele disse olha irmã a cobra mordeu meu pai. É? Que cobra é? uma jararaca. E cadê ele tá aí? Tá aí. Ele dizendo que tá doendo, tá doendo e tá adormecendo tudo a perna então:: aí eu disse assim: o senhor não sabe fazer algum remédio para picada de cobra será, ele não quer ir pro hospital, eu disse vamos ver, vou fazer como tinha muito cubiu ele tava morando aí... que vamos fazer eu mandei a mulher fazer. Pisa bem tira o sumo, um litro de água e dê três copos pra ele beber e vamos lá já socorrer homem. Chegou lá de tarde a gemendo: ai, ai, ai, ai não posso mais suspender ~~entender~~ a perna. E aí? Eu disse para ele: irmão trouxe um remédio pra ti e que Deus abençoe esse remédio cê vai ficar bom já já. Será irmão? É. Toma um copo desse agora, 6 horas da manhã, 11 horas. Se prepara para tomar outro, 12 horas toma outro. 5 horas (inint) [00:08:50] ele tava andando esfriou tudo, aconteceu nada então ele já foi trabalhar de novo.

A: o senhor poderia descrever para mim que sintomas o quê que a pessoa sente quando é picada por cobra?

OF3: olha eu:... essa dor, a gente se senti muito ruim né porque(...)

A: (...) sente dor, cospe o sangue como o senhor falou (...)

OF3: (...) é bota o sangue. E ela vai doendo (...)

A: (...) o local?

OF3: o veneno, parece que vai entrando pelos ossos. Os ossos vai adormecendo tudo. Então é isso aí, que só isso aí mesmo.

A: esse segundo caso que o senhor falou. Quanto tempo depois de ser picado ele tomou o sumo do cubiu?

OF3: só três vezes. Só.

A: ele tomou três vezes? (...)

OF3: (...) é, no dia só.

A: mas quantas horas depois de ser picado?

OF3: depois de ser picado nada mais.

A: entendi. O senhor poderia:: o senhor saberia me descrever em que situações é... a pessoa que foi picado por cobra agravaria, algumas coisas, que restrições o que que ela pode não pode fazer quando a pessoa picada por cobra? Tem algum resguardo, alguma coisa remosa?

OF3: não, não, não, não.

A: não tem? Pode (...)

OF3: (...) não porque tudo a o veneno (...)

A: quando faz o uso do sumo da folha do cubiu a gente (...)

OF3: (...) é. agora só que não pode comer alguma coisa assim (...)

A: que coisa?

OF3: ela é::.. (...)

A: (...) ela é forte.

OF3: (....) ela é forte.

A: e aí o que que não pode comer quando ela cobra e toma esse mês esse medicamento natural?

OF3: não pode comer uma fruta contra... uma fruta azeda.

A: (...) que frutas?

OF3: é:: seja abacaxi (...)

A: (...) abacaxi

OF3: é outras frutas assim (...)

A: (...) cupuaçu?

OF3: é, cupuaçu, mas três dias depois já pode. Tem nada que possa impedir.

A: é, como que::.. como que as pessoas podem se proteger pra não ser picado de cobra?

OF3: olha:: aí só se::... que as vezes a cobra é traiçoeira né. as vezes ninguém vê.

A: uhum.
OF3: ela é mais agitada. por exemplo a surucucu aqui é mais traiçoeira né? O que que tem a surucuru tem mais de 3 metros. Ela... você vai passando ali, quando ela tá braba não queria ninguém de lá ela solta o cumprimento que ela é. Pá na bunda, cara só pega mesmo nas nádegas. Não tem outro canto não. Alguma vez pega na perna, na coxa mas é na nádega, pá e o dente dela não é pequeno né. Desse tamanhão aqui. Cai e rasga quando ela puxar o peso e só peso joga o cara lá. Então aí não tem mais como ele se::.. alevantar, aguentar porque ela já:: sobe rapidinho e tem que correr no rumo...na segunda vez aí o cara não aguenta mais não. Assim já a primeira o cara não aguenta (...)

A: (...) a maioria das picadas de cobra elas são na perna teria algo que pudesse ser feito para se proteger?

OF3: olha, (inint) [00:12:43] excelente, posso dizer agora que pode se proteger seria ele chegar e matar a cobra.

A: matar a cobra seria melhor proteção antes que ela atacasse?

OF3: risos.

A: na dúvida mata cobra? Se:: se essas pessoas que são picadas de cobra geralmente elas estão fazendo o quê? trabalhando em que situações?

OF3: as vezes caçando né e às vez no caminho da roça, ela pega no caminho da roça e é assim quando ela pega por aí na... no mato né:: caçando é mais ruim (inint) [00:13:24] é por isso que sempre o caçador anda de dois por se que acontece alguma coisa com parceiro tem outro pra socorrer. (...)

A: (...) tem outro pra carregar?

OF3: é.

A: o seu Felisberto se você estivesse... as pessoas tão:: são picada de cobra à medida que elas estão expostas entrando na mata, caçando, pescando. Se vocês recebessem botas, perneira pra proteger vocês usariam?

OF3: Olha quando.. quando ele:: alguém desse pra ele:: porque pra ele pode comprar não tem muito dinheiro.

A: Mas vocês recebessem vocês usariam?

OF3: embora que ele use a perneira, seja a bota mermo, mas não prevalece, ela ofende sempre (...)

A: ela vai sempre ser um risco?

OF3: isso. porque é muito grande o dente da cobra. E é:: muito rápido, né? mais pior de que uma agulha de injeção se picar já era.

A: e quando uma cobra picado:: quando uma cobra pica a pessoa ou quando vocês avistam uma cobra o que que deve ser feito?

OF3: quando o cara vê assim que a cobra já tá... ela já tá pronta.

A: uhum.

OF3: Se tiver com um terçado, uma corda, ele corta logo.

A: tem que matar a cobra.

OF3: isso.

A: E se... e se for picado também mata que::... que as pessoas normalmente fazem?

OF3: olha, se ele tiver:: se ele não tomou muito susto e viu a cobra dá pra ele matar mas é bem difícil. Agora se anda com outro companheiro e... o:: companheiro resolve. Mata a cobra e aqui pra esse lado aqui como tinha muita... surucucu e jararaca. (...)

A: (...) pra que lado?

OF3: (inint) [00:15:25] cobra papagaio, quando ele tá braba lá desce de lá fica pendurado, você vai passando... essa daí só pica no rosto.

A: que é a cobra que fica no galho?

OF3: isso.

A: e o senhor já viu algum acidente dessa cobra?

OF3: já.

A: como é que foi? Conta aí pra mim.

OF3: a cobra... essa cobra papagaio pegou ele atrepando açaí (...)

A: (...) uhum.

OF3: lá em cima, pá (inint) [00:15:48] (...)

A: (...) ela tava no cacho de açaí? (...)

OF3: é. Desceu foi rapidinho de lá. Parece que não tava nem segura. Chegou no chão já era não podia mais enxergar.

A: e onde a cobra picou ele?

OF3: no rosto.

A: no rosto.

OF3: quase que pegava (inint) [00:16:01]

A: o que que ele sentiu? (...)

OF3: (...) ai foi colocando o sangue pela boca. ~~Parece que nem tava nem segura chegando no sangue, já não podia mais chegar e onde a cobra picou te pegar no colocando a boca.~~ É sangue pela boca, é rápido isso aí também.

A: e ele tava distante de casa?

OF3: tava distante, daquele outro lado.

A: e o que que foi feito? (...)

OF3: (...) de outro para cá para trás.

A: outro caso?

OF3: é

A: também foi com a cobra papagaio?

OF3: papagaio.

A: Como é que foi esse caso aí? O que tava fazendo (...)

OF3: (...) ele atirou o macaco lá em cima e foi atrepar (...)

A: (...) pra pegar o macaco? (...)

OF3: (...) até pra chegar lá perto e a cobra papagaio parece que tava lá esperando. Ele nem deu fé não, quando ele viu assim, que ele foi subindo pra olhar... pá no rosto. Caiu, mais primeiro de que o macaco. Morreu na hora.

A: esse caso morreu.

OF3: morreu.

A: era uma pessoa adulta, um senhor adulto?

OF3: uhum. outro caso aconteceu com meu irmão (...)

A: (...) pode contar?

OF3: andando, pescando para aquele lado, lado da:: da boca do Camatiã e lá nós já saindo no varador, furo do lago lá..lá chamado Chian. Varando Canoa, um canozinho que tava enchendo o rio aí:: arraia picou nele tirando o pé ficou grudado assim, mas como eu...não tinha como fazer eu queria pegar pra puxar ai ele suspendeu fiz assim para cima como tá chutando aí saiu a... aí arraia já prejudicou minha saúde e minha vida. Agora eu vou morrer com isso eu não aguento não (...)

A: (...) doía demais?

OF3: sentou logo lá na Canoa e começou a gritar. Botou muito sangue, acertou bem por aqui assim. E aí o que que ia fazer? Aí eu disse eu vou tirar. Eu vou tirar esse cubiu fruta de cubiu::.. na capoeira aí que era do meu avô. Eu disse não meu irmão. Olha aí tem:... bem aí tem um apuizeiro e tem:: cipó ambé (que

nós chamamo aqui né).
E você vai lá tira um pedaço. ~~Tempera~~ Nesse tempo era menino corri para lá, já cortei um pedaço...mais trás assim, não trás assim vai escorrer o leite. Corre que eu não tô mais aguentando e vamo embora, baixando. Gritava no meio do rio aí, eu cortei um pedaço e trouxe assim. Cheguei ai cadê? pode derramar em cima dela bem... esfrega por cima o leite um leite que gruda muito bem, pois é isso aí tu vai se lembrar um dia e vai servir para você, você já aprendeu fazer::... aí passou, talvez uns 10 minuto tá indo não chegou em 10 minutos.
ele disse assim: olha meu irmão graças a Deus que tu veio comigo para me socorrer. Eu tô bonzinho, tá mais doendo ~~doido~~ não? (...)

A: (...) já tinha esfriado?

OF3: e rápido também e tem outra também que é bom pra isso leite da caxinguba é que o pessoal conhece como lombrigueira mas se não tem dessa outra mermo serve.

A: uhum.

OF3: corta, tira o leite (...)

A: (...) o leite da caxinguba fruta ou da árvore?

OF3: da árvore... corta a arvore e tira numa vasilha o leite e depois passa lá na figura da picada da arraia.

A: serve pra cobra também?

OF3: ela nem:... ninguém nunca experimentou não

A: experimentou pra arraia?

OF3: pra arraia.

A: E aí faz o quê? Coloca em cima?

OF3: coloca em cima. Dentro de 10 minutos tá curado.

A: é. não precisa nem ir pro hospital?

OF3: nem pro hospital não. É... (...)

A: (...) e o senhor aprendeu a fazer esses tratamentos com quem?

OF3: com meu irmão (...)

A: seu irmão mais velho do que o senhor?

OF3: mais velho que eu.

A: Ele que repassou esse conhecimento?

OF3: isso, que ele passou. agora tem outros tipo de remédio da selva né?

A: quais?

OF3: tem serve pra...pra a gente conhece assim como:: com o nome de yanamoco. Na língua kokama a gente chama yanamoco.

A: yanamoco.

OF3: yanamoco. isso

A: e o que que é? Uma arvore, uma planta?

OF3: é... ela é da mata.

A: da mata virgem?

OF3: da mata virgem.

A: é o que que a gente faz com ela?

OF3: a gente tira o... olho quem conhece tira a folha e machuca bem pode colocar na boca dele e ele engolir o caldo e passa lá onde::...

A: (...)onde foi picado (...)

OF3: (...) onde foi picado. E ela também não vai deixar inchar a perna, ela vai tirar aquela dor da picada. Não vai ficar assim porque ela fica roxo né.

A: uhum.

OF3: também ela fica:: fica naturalmente. Então isso aí é muito bom, mas só que hoje é difícil a gente conhecer porque quem conhecia isso aí era os nossos avós.

A: os mais velhos da época de vocês.

OF3: é.

A: E hoje o senhor se preocupa em passar esse conhecimento para alguém ou o senhor tá passando pra neto, pra filho como é que é o corre isso?

OF3: até no momento não sempre falo aqui pros meninos e ela serve pra zelar o dente.

A: (...) é o que?

OF3: fica brilhoso o dente.

A: ah. Pra limpar (...)

OF3: pra limpar o dente. (...)

A: (...) clarear.

OF3: é. Nunca vai estragar logo rápido seu dente. Porque ela fica que nem assim um ouro banhado... fica brilhoso (...)

A: (...) coloca uma camada de proteção.

OF3: isso.

A: entendi. e a... essa planta que o senhor falou que ela::.. que é mais raro ela tem aqui em São Paulo de Olivença?

OF3: aqui não tem não. (...)

A: (...) onde que o senhor conheceu essa planta?

OF3: olha isso eu conheci quando meu avô era vivo. Eu vi ele trazer do mato que eu não conheço nem como é né ~~pro médico~~ para fazer isso limpar os dente. Também tem::... remédio bom pra::.. próstata, também tem remédio bom para fazer isso que acaba a próstata e:: eu não consigo andar pelo mato e já tinha tirado para mim. Que é que tem:: aqui nós conhecemos ~~pouco~~ por cumaté. (espécie Myrcia eximia DC)

A: cumaté?

OF3: cumaté tira casca dela e você pisa bem, tira o caldo::... (cuidado com a caba). E aí a gente tem outro:: tem outro:: outro remédio aí também caseiro que a gente mistura. Inclusive conhece e... aí eu tenho aí, plantado bem aí.

A: jambu.

OF3: jambu. E.. aí você mistura lá dentro, isso aí você ferve bem, põe no sol e aí você vai tomando ela::.. é muito bom.

A: é seu Felisberto existe algum tipo de proteção que pode ser feito pra fechar o corpo com uma reza alguma coisa assim pessoas que vão caçar, vão pescar, se proteger?

OF3: existe né, existe por quê (...)

A: (...) Como que é feito?

OF3: se proteger pra dor da cobra essas coisas no igapó assim tem muito. (inint) [00:25:57] E aí ela pica o cara né, mas...mas antes disso você... você reza a oração pra contra e a oração dela é assim: (canção)

OF3: Então essa oração é longa (...)

A: ela é::.. o senhor cantou um trecho dessa oração?

OF3: é.

A: e essa oração protege o meu corpo?

OF3: é. Tá protegido se vai::.. agora não é bom também cantar assim à toa como uma brincadeira porque aí prejudica você. (...)

A: (...) entendi.

OF3: Ela vem (...)

A: (...) vem de proteger (...)

OF3: (...) as cobra vem se reunir pra saber o quê que você tá querendo.

A: entendi.

OF3: é isso aí que protege a gente, com essa oração.

A: essa oração é cantada pela pessoa ou eu vou tipo no rezador no pajé para ele cantar pra mim?

OF3: não a pessoa que tem que aprender a cantar e ele nem todos sabem isso aí dificilmente.

A: é? Quem... quem que sabe essas orações?

OF3: só eu.

A: só o senhor e aí o senhor ensina pros seus filhos essa oração?

OF3: não. Que é difícil a gente aprender agora a gente faz ele::.. é alguma apresentação (...)

A: e ela é cantada em que língua?

OF3: kokama.

A: kokama.

OF3: uhum.

A: Teria alguma coisa a mais que o senhor quisesse acrescentar em relação a tratamentos a... de picada de cobra?

OF3: tem. É... ela... a pessoa picada de cobra ela tá doente, né? Tá doente:: porque na época ninguém sabia fazer esse remédio aqui. Os velhos não sabiam fazer esse remédio não, nem todos sabiam fazer. E aí quando o sujeito tá assim porque tá cuidando paciente assim. Toda manhã, de tarde:: e tem que rezar essa oração:: dei a introdução dela pro paciente lá onde tá picado, pega a::.. uma agulha, tem uma... uma coisa que chama que nós conhecemos como xingo xingo, é uma paradinha (inint) [00:28:36]. Cantando, cantando, vai::... ela:: tem que parar a dor aí pra poder tratar na época meu avô faziam assim e eu via eles fazendo também. Se ele já::.. e nessa época aqui eu já conhecia São Paulo de Olivença talvez tinha umas 5 casa (...)

A: (...) uhum.

OF3: e não existia médico, enfermeiro, não tinha posto nenhum. Bicho... se tratava assim quando picava e era muita cobra que tinha na época. Agora já tem:: tão matando muito corre mas pra longe. E assim mesmo, aqui mesmo eu

matei jararaca aqui dentro desse cerrado. Não é muito bom deixar os capins crescendo. Bem agora já tá bom, já pode andar, então agora vamos fazer outra coisa. O velho disse: vamos fazer aqui, tragam a tinta e::... e tem uma tinta aqui que chama de kurimã, na língua kukama: kurimã. Tira (inint) [00:30:01] o sumo do caldo do jenipapo. E aí ele vai amanhã você vai tá andando amanhã, toma banho, você tá de alta amanhã, mas agora nós vamos fazer o seguinte: eles tiravam assim dois pedacinhos de flecha (takana) e corta, faz uma S e aí vão pintar o doente todinho, no rosto, tudo, tudo, tudo que nem uma cobra. aí ele faz uma... uma cobra de pau, fazem 2,3 cobra de pau. aí ele sai cantando de manhã cedo cantando essa oração aí essa oração. Todo mundo tá pronto? Não é só ele que vai ser pintado, todo mundo que tá assistindo aí vão assisti, participa que ele vai tomar banho pra é:: deixar o veneno tudo nagua. Aí tá todo pintado e as cobras também tudo pintada. Ele disse assim: (língua kukama) que mui é cobra.

A: mui é cobra?

OF3: mui é cobra. (língua kukama). Pega um pau então e bate a cobra faça de conta que a cobra tem medo aí eu disse: agora canta (canção na língua kukama). Os menino que tão tudo lá pintando é papai, vão levando, cantando canção pela mata, na água. Ele cai pra:: toma banho. O veneno já foi submerso embora, lavou o corpo e sai de lá todo alegre, cumprimentando...(inint) [00:32:36] Deus olhou para você.

A: é um ritual para eliminar o veneno?

OF3: isso.

A: e aí ele é feita em conjunto com aquelas pessoas que estão aqui. Todo mundo se pinta, todo mundo parte a cobra (...)

OF3: (...) é. E parte a cobra, outros que vão arrastando no chão. Os meninos vão com pau batendo a cobra e outro vai arrastando né.

A: Existe algum tratamento que o senhor conheça ou que o senhor faça que (faz a partir da cobra) [00:33:07] sim ou não?

OF3: o tratamento que eles fazem aqui... é... eles dão o remédio também pra beber né... remédio. Agora o remédio é::... é que eu esqueci o nome do remédio. Ela é da mata também::.. pa ajudar ele a ficar bom né. é um remédio aí, mas quem sabe bem é::.. minha esposa mas ela não tá aqui.

A: ah, entendi. Seu Felisberto o:: muito obrigado. Gostei muito de conversar com o senhor é:: o senhor precisa passar... escolher seu neto, seu filho pra passar todo esse conhecimento pra que daqui um tempo ele continue disponível pras pessoas que vão nascendo, vão crescendo tá bom?

OF3: uhum.

A: Muito obrigado.

OF3: nada.

[00:34:09]

Entrevista Cuidador 4

Idade: 64 anos
Religião: Católica
Neto de praticante da medicina natural
Pai peruano e morou por 20 anos no Tauaru.

Informações:

a. Destacou o uso da folha do açaí novo, casa de barro da formiga no tratamento de acidentes ofídicos e a defumação com unha, bico e penas de gavião para proteção.
b. Costuma entoar cânticos de proteção e utiliza o chá da ayahuasca para diagnosticar a enfermidade/doença das pessoas que o procuram e a partir daí define o tratamento para o paciente.
c. Ao final da entrevista, mostrou o ambiente em que atende os pacientes, os utensílios usados e alguns produtos produzidos para o tratamento de doenças que necessitam de massagens e fricções.

[00:00:01]

A: Seu Bides eu gostaria de saber inicialmente como o senhor aprendeu a fazer o uso da medicina tradicional?
OF4: Sim, então isso já vem assim... de herança.. de ancestros velhos, meus avós eram grandes médicos da natureza na época quando não tinha.. os... a medicina de... dos médicos ne... então não se tratava minha família. A maior parte era tomando ayahuasca so que a ayahuasca quando se toma vamos a capitar ~~ver~~ que doença tem pra lançar outro remédio. No caso que se você tem uma gastrite

adquirida nós vamos preparar um tipo pra poder matar essa bactéria e sarar. Se esse problemas de dores, articulações, nós vamos fazer essa (tervio) preparo uma pomada para fisioterapia e passar o remédio preparado para tomar. Então se você tem uma quentura na cabeça já preparo o remédio pra tirar essa quentura e passar... então pode ser um fortificante, uma vitamina se é necessário se não so com remédio naturais vamos tirar essa quentura que esta na cabeça que nosso conhecimento (não entendi) 01:37.

A:então você... se utiliza ayahuasca pra fazer o diagnóstico?

OF4: para diagnosticar e na mesma hora para expulsar coisa negativa e jogar também de meditar e olhar como tá seu dia a dia, como tá minha vida e o que que eu devo fazer a partir de que eu participo para me educar, é como uma repreensão, um purgatório.

A: entendi. E durante a sua... a sua vida o senhor viu algum... alguma pessoa ser picada por cobra? Você já tratou alguém picado por cobra? Me conta um pouco alguns casos aí.

OF4: Sim, então quando não tava... isso era mais ou menos em anos década de 85, 80 nessa faixa etária.. então se foi picado por uma jararaca um senhor então ela achava que morria. Como pra lá, acho que você deve ter uma curiosidade que tem uma formiga que... desse tamanho que fazia um ninho de barro (...)

A: (...) uhum...sim

OF4: pode ser branco ou vermelho esse barro. tu arranca isso e imediatamente tu coloca agua limpa, tu vai despejar, misgalhar e vai tornar liquido. Ai você vai trazer a folha do açaí, cê vai machucar... de preferência as folhas mais verdes que tiver, as folhas mais verdes, mais novinhas, que tiver viçosas, machuca pra valer um monte e mistura nesse barro e pode dar de tomar. Assim que nós fizemos com ele pra poder no morrer.

A: o que que acontece quando toma? Qual efeito que dá?

OF4: bom. Ele tomando bastante ele vai vomitar (...)

A: (...) uhum (...)

OF4: (...) então ele vai ajudar a baixar já o... a baixar a força do veneno (...)

A: (...) uhum...entendi.

OF4: agora outra oportunidade quando nós tivemos um cachorro (...)

A: (...) picado por cobra?

OF4: aham, nós tavamos fazendo caçada com cachorro si pra meios de vida e quando uma cobra venenosa pegou na venta... foi uma surucucu. Então ter um animal muito profissional sobre caçada. Que que nos restou? De pegar ele, parou a caçada, e levamos ~~casa~~ imediatamente e por bem que sempre atrás no sítio tinha esse...essa formiga que tem aquele barro (...)

A: (...) uhum (...)

OF4: (...) só que aí usamos diferente. Pegamos a castanha, tiramos a castanha mesmo, machucamos bem mesmo, tiramos o leite. Então esse leite já foi na água diluído do barro, colocamo leite condensado, nós demos por cachorro, o cachorro não morreu.

A: (...) não morreu (...)

OF4: então essa duas coisas... e tem também a capeba. (***Piper umbellatum***)

A: sim.

OF4: a capeba que na mata. Não sei se você já olhou (...)

A: (...) a que tem espinho?

OF4: não. É uma...uma planta que pega já nos paus quando tão se apodrecendo. Uma folhinha desse tamanho bem direitinho.

A: e aí, o que que cê faz com ele?

OF4: então, isso. Pega as folhas mais novas, lava e machuca bem, o mesmo procedimento pega a palha do açaí e o barro. Bem coado, duas vez coado e pode dá (...)

A: (...) a palha do açaí, a capeba macerado, mais o barro da casa da formiga?

OF4: é. Esses três componentes. Agora quando a leson és grande, o golpe, se tiver assacu *Hura Crepitans)* por perto, tira um pouco no algodón e colocar onde foi mordido e passa uma pequena atadura aí (...)

A: (...) o leite do assacu?

OF4: aham, ele vai absorver o veneno.

A: entendi.

OF4: então, isso son com relacion (...)

A: (...) aos tratamentos... os principais tratamentos

OF4: aos meu conhecimento.

A: e geralmente essas pessoas que o senhor viu, tratou com essa medicina tradicional, medicina indígena, medicina... o que... o que que essas pessoas estavam fazendo quando foram picadas?

OF4: andando na mata.

A: em geral andando na mata?

OF4: a maior parte... é que... é quando você tá andando e... época de caçada, apanhada de açaí ou tirando cipó, qualquer coisa que oferece o/a floresta né, que você vai por um motivo. Desde que o cê entra na mata e aí os cuidados, porque a Amazônia tem lugares que tem muita cobra.

A: existe alguma época do ano que tem mais, que tem menos?

OF4: olha.. na maior parte.. sim, sim, inclui a época. Aqui por exemplo no ribeirinho, no inverno, na alagación, estan perto da beira do rio. É muito perigoso para ser picado porque devido a alagação ele vem procurando terra mais alta. E aonde é muito risco pra ser picado por ele.

A: o que que as pessoas precisam fazer pra se proteger das cobras?

OF4: oh. É... tudo isso que a natureza oferece pra gente (...)

A: (...) uhum (...)

OF4: primeiro quando a criança nasce (...)

A: (...) uhum (...)

OF4: você conhece a águia cobreiro que diz nos livros dos editores?

A: do cobreiro?

OF4: águia... cobreiro... tá no livro. Isso se chama acauã. *(Herpetotheres cachinnans)*
Um gavião que pega cobra.

A: ah sim. Águia. Sim.

OF4: acauã (canção)

A: ah acauã (...)

OF4: (...) por isso se chama águia cobreiro. Disse no livro.

A: sim. ok. Conheço.

OF4: pois é. Então quando criança tu já. Na lua nova, quando mata um desses gavião tira a pena, bico e a unha. Aí tu vai derrubar ai queima um pouco dá o chá e da (...)

A: (...) pra criança beber? (...)

OF4: (...) uma colherada. Deixa ela... que tu queima a unha, bico e a pena. Três lua nova dá aí as cobra invés de te morder vai sair logo. Você vai olhar logo.

A: afugenta a cobra?

OF4: é porque você fica já curado e tua já volta em cima de cobra... não aguenta tu olhar.

A: entendi. Então essa é uma forma de proteção importante?

OF4: de maior parte todos os gaviões protegem o homem do campo. Porque todo o gavião
pega a cobra. Por isso os gaviões colocam onde estão abacaxis bravos ai eles colocam os ovos, ali estão olhando é tudo. Tudo.

A: uhum.

OF4: nós temos o canto do gavião.
(canção)

A: é um... é um cântico de proteção? (...)

OF4: (...) é (...)

A: e normalmente se (...)

OF4: (...) se canta quando tá doente (...)

A: (...) ah. canta quando tá doente (...)

OF4: (...) canta quando tá doentinho assim. Qualquer doença... delicadeza criança.

A: entendi. E quando uma pessoa vê a cobra o que que a gente deve fazer? Quando vê uma cobra.

OF4: bom, normalmente. Que se nós vamos... usar por exemplo questão do homem (não entendi) biólogos, deixa...deixa... mas a natureza não diz isso não. Qualquer gavião que olhar uma cobra, é meu cardápio. Não vai errar não. E no objetivo, nós, é matar ela. Porque são inimigo nosso... porque tu não tá andando pra querer olhar como biólogo quando é uma cobra tu estas andando distraído... quando ela te pega a traição. Teu pensamento tá outro querer ver uma casa, querer ver um cacho de açaí, cipó. Você não vai olhar pra baixo, tu pisa, pow! Então esse é um desafio. Agora tem ilhas aqui.. como tu deve conhecer...Ilha de Arariá acima de Prosperidade. Muita, muita, muita, muita, muita cobra só num dia roçando nós matemos 56 jararacas só daquelas açú (se referindo a jararacussú). Entendeu? Então tem lugar nessa Amazônia que tá topetado de cobra.

A: o seu Bides. Quando uma pessoa é picada por cobra, faz o tratamento como o senhor já descreveu. O que...o que mais precisa. O que ela não deve fazer além do tratamento. O que ela pode fazer... que se ela fizer vai agravar? A (...)

OF4: (...) primeiramente que uma mulher gravida não tem que olhar, um mulher que dormiu, que fez carinho entre marido e mulher não pode olhar. Porque se olhar pode dá uma recaída ou levar a morte.

A: por quanto tempo tem que ter esse resguardo?

OF4: pelo menos minimamente ele tem que ter segundo o atingimento que ela teve pelo menos ele tem que... pelo menos três meses (...)

A: (...) três meses (...)

OF4: (...) para no da recaída.

A: então.. então ele não pode ficar próximo de uma mulher que fez relação sexual com o marido e não pode tá perto de uma pessoa (...)

OF4: (...) nem olhar (...)

A: (...) nem olhar (...)

OF4: (...) nem conversar, nem escutar a voz da mulher que tá assim.

A: O quê que acontece se (...)

OF4: (...) e muito (melhor) [00:11:17] se tá no período menstrual.
A: é?

OF4: também (inint) [00:11:19].

A: também no período menstrual. O que que acontece nesse três casos? o que que a pessoa picada de cobra vai sentir?

OF4: a dor vai voltar em dobro e vai começar a gritar.

A: o senhor já viu casos que deu complicações a partir disso? (...)

OF4: (...) já.

A: o senhor poderia relatar um pouco para eu ter uma ideia melhor disso?

OF4: então... um:: um:: um senhor foi mordido de cobra, ele já tava bem com 30 dias, mas de repente uma mulher...nem... nem olhou somente escutou a voz que passou perto::... não deu 5 minutos o homem começou a gritar. Isso preocupou muito::... como sempre às vezes na época a gente trabalhava muito forte e nós tinha todos preparos, nós tinha os preparos pena, tudinho do gavião, fomos arrumar e tomar esse cipó pra ritual. Cobra, cobra venenosa, cabeça de para raio [00:12:19] (canção).

OF4: então já vamos se obrigar a fazer a concentração pra poder levantar se não morre.

A: entendi.

OF4: Antigamente era muito pesado esse lado, hoje em dia não pois facilita melhor. Já tem um socorro vai tomar isso, já vai sair rapidinho também tá para chegar para tomar uma contra veneno, mas não vai morrer porque vai tomar aquilo que te falei.

A: Entendi. O senhor acha que o:: a pessoa que é picada de cobra deve ser levada pro hospital tomar soro?

OF4: é, por uma seguridade, que muitas vezes, ele pode ter outras delicadezas qualquer problemas orgânicos assim corporal. Então ele pode ter uma delicadezas e nessa delicadezas pode ter sua imunidade baixa pode ficar doente e ela pode se acumular em dobro e não resistir.

A: entendi. É... seu Bides é::... Existe algum outro tipo de proteção além da proteção espiritual do Gavião também do chá do gavião que possa ser feita do cântico que o senhor falou que possa ser feita pra proteger quem se expõe na mata pra::... (...)

OF4: (...) Sim (...)

A: (...) que tipo de proteção? (...)

OF4: quando vão entrar na mata, pois nós vamos a fazer a concentração todo esse canto da natureza das arvores, plantas, tudo isso aí, são muitas criaturas que toma (inint) [00:14:11] maestro: proteja o meu marido tá na mata. Aí nós vamos invocar o nome, aí nós vamos pedir permissão da natureza, nós vamos invocar pra poder proteger essa criatura que tá no campo então se a gente coloca já os gaviãs para cuidar dessa criatura. Todos são mistério da natureza.

A: entendi.

OF4: (inint) [00:14:31] que a natureza é... infinita seu mistério. A natureza é toda pra nós.

A: entendi. A:: o::.. o gavião o senhor entende, interpreta é::..reconhece o gavião com um::.. agente protetor do homem?

OF4: sim. Na mesma hora uma medicina também. Vamos dizer que o maestro tá se formando. Tá se formando. Tá querendo ser um maestro aprendiz também e eu sou natural, nada ninguém me ensinou. Eu já nasci com isso... só de morder uma folha eu já sei pra quê isso. Então quando a pessoa vai se proteger dessas coisas aí, tu mata o gavião, tu não tira a pena, aí tu vai colocar panela grande, cozinhar bem... bem... até ficar pouquinho, aí tu vai tomar esse caldo do

gavião né:: só o caldo. Você vai dietar 20 dias. Aí tu pode entrar em contato já com o gavião.

A: entendi.

OF4: tem um canto dele. O gavião.

A: como é o canto do gavião?

OF4: canto [00:15:42]

OF4: esse é o canto do gavião.

A: quem pode... quem pode utilizar esses cânticos, quem que pode utilizar essa medicina que o senhor conhece?

OF4: bom, não pelo egoísmo, mas desde que a pessoa por exemplo::.. vamos dizer que tu participa e se você gravar e se tivesse algum lugar tu tomasse o vegetal...tu alcança ter esse objetivo...desse mecanismo... a gravar na sua mente...tu pode cantar...tu pode fazer uma obra pra uma criança.

A: entendi. Utilizando vegetal como mediador.

OF4: Exatamente (...)

A: (...) desse conhecimento.

OF4: sim aí a natureza te autoriza.

A: entendi. É:: e pra:: e pra etnia kambeba indígenas que o senhor tem contato também é:: o quê que a cobra representa?

OF4: bom. A cobra (inint) [00:17:34] também, principalmente de hoje em dia:: é muitas por exemplo existe também um:: aprendizado estranho também do ser humano isso eu não tenho isso. (inint) [00:17:49] testemunhar esses:: essas feras, aprender a coisas... coisas vamos dizer a coisas estranhas, não vou falar negra... coisas estranhas. (...)

A: (...) (inint) [00:18:07]

OF4: vai começar a dominar uma cobra e se tem raiva vai te fazer morder isso que não concordo. Eu nunca aprendi coisa do mal.

A: mas isso pode acontecer de uma pessoa tem um domínio (...)

OF4: (...) acontece , acontece. Domínio e manda que morde.

A: como que acontece isso?

OF4: que morde. Você nem percebe quando morde.

A: mas quem? Que tipo de pessoa que:: domi::.. que tipo de conhecimento são essas pessoas que têm que domínio que ela tem? Como é que isso acontece?

OF4: são pessoas que já se especializam com poderes::... com outros poderes.

A: uhum.

OF4: outros poderes. Porque enviam uma serpente pra te morder ou te fazer engolir com a cobra maior.

A: uhum.

OF4: Atalaia tem. Eles fazem isso, mas eu já repreendi muita gente de lá. Não é pra fazer isso. Não é pra fazer isso.

A: então existem praticamente duas::.. duas razões para as pessoas serem picadas por cobra?

OF4: é...é.

A: uma delas é pela exposição que a gente já conversou bastante (...)

OF4: (...) sim.

A: e a outra é quando alguém pede ou pra um feiticeiro ou conhecedor de um determinado conhecimento (...)

OF4: e graças a Deus não sou desse lado e também hoje em dia porque hoje em dia ela vai servir para medicina também.

A: A cobra?

OF4: é. o veneno e também assim por exemplo: tem pessoas que preferem o dente da surucucu para pedir autorização da natureza colocar num colar e apresentar um (serviço), um inimigo para (inint) [00:19:42] de surucucu se

espantar (inint) [00:19:53] pra mim não tanto me::.. eu tenho essa autoridade (zelar) mas tô falando o quê que ela faz (...)

A: (...) sim. O que acontece. São os caminhos.

OF4: e mesma coisa a jiboia, por exemplo a jiboia tem uma:: pedrinha aqui na cabeça (...)

A: (...) hum.

OF4: nem toda jiboia também. Uma jiboia que tem umas pintas bem::.. então quando vai:: tu tá pesquisando dentro da mata, uma jiboia desse tamanho, o passarinho voa, voa, voa pra cá, pula pra cá e quando já tá todo ele abre a boca o passarinho sozinho ele cai. E tem muitas pessoa que utiliza isso:: realmente o meu modo de pensar não. Não porque todos somos capazes.

A: uhum.

OF4: agora o incapazes que não sabe descobrir sua mente ele vai fazer isso, fazer isso colocarem perfumes...colocar aqui, colocar aqui. Aumentar a força visual, encandear a pessoa e no teu pensamento diz "você eres meu", e a pessoa que não é preparado vai magnetizar mesmo como a jibóia...

A: então existem é:: formas::.. é:: algumas formas de quem usam como amuleto pra (...)

OF4: é porque isso vai depender de cada crença, de cada pessoa. Se tu dizer que não vai fazer isso tu não vai. Desculpa te dizer mas...se tu não tem fé... tu não tem fé... mas se tu disser que sim, eu vou conseguir, você vai.

A: entendi.

OF4: o que na... a realidade todos os seres humanos somos capazes sempre quando a gente quer.

A: é:: me fale como... a partir de que idade que o senhor começou a fazer esses tipo tratamento, esse tipo de uso da natureza em benefício dos (...)

OF4: (...) desde os 18 anos.

A: é. Como isso ocorreu? como foi (inint) [00:21:42].

OF4:bom. Isso começou quando eu trabalhava, eu morava no Incra em Tabatinga:: na colônia. Um dia... de nada eu fiquei perdendo a força respiratória, o ar. Porque uma época fiquei, fiquei uma hora e meia cego dos dois lados, não olhei nada. Depois que aconteceu isso aí eu teve que correr na estrada como se fosse a... morto e no outro dia chega uma mensageira disse: Bides, acompanha meu marido tá doente, apareceu umas coisas horríveis, tá dizendo que ele é leproso e eu quero que tu acompanha lá. E quando eu fui tomar com maestro lá em Tabatinga. Então na noite que eu tomei, eu fiz bater muito meu peito e começou a falar tanta da coisa assim, tipo um discurso eu concentrado com a evolução do remédio e teve naquela época um arigó ficou olhando deixando de se concentrar pra olhar. Começaram a falar sobre:: assim... últimos tempos (inint) [00:22:38] a convivência do ser humano o que que vai acontecer? muita coisa foi indo, foi indo. Aí nessa primeira noite Nazareno vem aqui. Vem aqui, você vai ser atendido pelo maestro. Eu nunca teve porque eu havia de herança meus avós eram ayahuasqueiros.

A: entendi.

OF4: então desde lá pra cá começou minha fama em Tabatinga. Comecei a atender, atender, atender tanta gente, eu acho que não conseguia mais dormir. Por atender tanta gente. Turista muito, muito, muito são meus fã. Por que eles gostam, eles não acreditam em outras idolatrias, como negócio de macumbaria outras mutretas não, mas esse vegetal por um cipó desse eles pagam 100 dólares, 80 doláres eles te dã para levar e pendurar na sua casa.

A: o vegetal também pode ser usado para o tratamento de picada de cobra?

OF4: Olha pode ser sim. Depende que ele já chegou um pouco baqueado. Ele vai ter que tomar e nos vamos ter que trabalhar, nós vamos ter que trabalhar.

A: ele ajuda a neutralizar o veneno? (...)

OF4: (...)ajuda, ele ajuda sim. Nos tratemos uma pessoa que foi mordido de onça que foi torado a clavícula, isso aqui. Tudo, tudo o pé. Ele ia morrer. Nós trabalhamos três meses pra levantar seu Pedro. Ele ia morrer, ele ia viver. Ele trabalhava no Takana Pedro Macedo Tananta.

A: entendi. E aí foi feito esse trabalho?

OF4: aí nós fomos a tomar vegetal, aí mulherada e homens cantando todos rituais da natureza e outra coisa onde, onde por exemplo eu me concentro nessa casinha de trás, você não vai escutar nem que tipo de coisas negativas porque a

natureza vai repreender o mal porque não lutamos contra o mal. Esse é nosso objetivo.

A: o senhor frequenta a mata, vai mata?

OF4: sim, tenho sítios, tenho plantios isso aqui tudinho. Isso aqui.

A: e quando o senhor vai pra mata, o senhor vai protegido? como que o senhor vai?

OF4: sim. Eu já, já tenho essa proteção por completamente.

A: o senhor usa a proteção mas (não usa a proteção física) [00:24:58]

OF4: já tenho já (...)

A: (...) o corpo fechado.

OF4:Então quando as pessoas vão pra lá, minha mulher, minhas filhas vão pra lá, ele sente medo porque ele sente uma presença assim os duendes que tá na mata e tal. Então quando eu vou lá... todos eles você (inint) [00:25:18] qualquer barulho que escuto vou lá, quero saber o que é.

A: entendi.

OF4: porque muita gente confunde a biologia por exemplo:: não alcança dele de acolher a ciência dessa natureza. Tu sabe que são vários tipos de duendes que tem, não sei se você entende por duende você estudou muito. Então o duende depois apresentou uma anta que confunde vai embora que tu vai te perdendo, mas se tu tá preparado não acontece nada.

A:não acontece.

OF4: não. Você vira a camisa ao contrário (inint) [00:26:05] três vezes e vai embora, mas se você não tiver preparação e mesmo tu já tem um pressentimento se é coisa do bem ou do mal.

A: entendi. Pra (definir) [00:26:14].

OF4: ah é. Não adianta muito. É um pressentimento.

A: o senhor nunca foi picado por cobra, por arraia, nunca?

OF4: não, não. Já tava assim que tinha uma percata que era assim::.. tinha tronco de lenha e a jararaca tava (inint) [00:26:35] em cima da... dá correia da chinela (inint) [00:26:43] porque porque (...)

A: (...) pra proteger.

OF4: é, pra proteger. Já andei muito pra criar meus filhos, caçava dia e noite:: muito difícil na época que eu morava em Tabatinga anos 80 quando tabatinga tinha pouquinhas casas, 75 sabe:: essa faixa etária. Tabatinga cresceu do nada.

A: Tem algum caso de acidente ofídico por exemplo de picada de cobra assim que o senhor gostaria de relatar antes da gente encerrar a entrevista? Que seja bem peculiar, o senhor lembra:: deve ter conhecer vários né. O senhor já descreveu uns 2/3 teria mais algum?

OF4: não. Pelo que eu saiba é isso.

A: são esses.

OF4: é.

A: É::.. e:: o senhor considera importante não o senhor que outras pessoas andam protegidas fisicamente como uma bota, uma perneira? Isso o senhor acha que ajuda a se proteger?

OF4: é, ajuda.

A: ajuda?

OF4: ajuda.

A: o senhor aconselharia uma pessoa que não tenha proteção espiritual (...)

OF4: (...) sim sim.

A: a utilizar equipamentos? (...)

OF4: (...) sim, inclusive quando eu ia para aldeia levava de Tabatinga presente de bota para elas que não nunca calçaram botas sim eu levava.

A: entendi. Então na sua concepção se o indígena estiver o equipamento ele vai usar (...)

OF4: (...) vou prevenir, vou prevenir.

A: para prevenir.

A: É::.. existe alguma dieta especifica pra quem é picada de cobra?

OF4: sim. Ele vai comer os alimentos adequados

A: e quais são esses alimentos adequados?

OF4: alimento que não tenha, que não contenha sal. Assim::.. a partir do mês pode comer um pouquinho de sal, coisas oleosas pra não... pra não irritar, pra não dar corrimento, e::.. coisas pesadas também não porque ele tá superando essa dieta.

A: e o quê que seria essas coisas pesadas? Peixe(...)

OF4: (...) um peixinho assado, caldo de peixe, uma Bananinha assada com toda casca.

A: ele vai comer qualquer peixe?

OF4: olha de preferência ele vai comer curimatã, cará, essas coisas aí.

A: peixe ele pode?

OF4: aham. Nem todo peixe ele poder comer (inint) [00:29:18] como assacu, urtiga, fruta de assacu ele não pode, ele tem que comer ou pode comer uma galinha caipira também assada sem encostar na brasa.

A: entendi. São mais (defumadas) [00:29:33] (...)

OF4: (...) ou uma ave do mato.

A: isso ele pode comer?

OF4: pode.

A: e o quê que ele não pode comer? Quais são os peixes?

OF4: principalmente assim alimentos remosos como: pirarucu, galinha de gelo, feijão (muito) [00:29:45] pesado é:: essas coisas que tem muito tempero.

A: aí tem que ter uma dieta mais leve?

OF4: é. Leve.

A: e o senhor durante sua vida já viu pessoas morrem por acidente de cobra?

OF4: já.

A: Em que situações?

OF4: é porque chegara e ele só quer existe :: assim quando chega no lugar por exemplo tu nunca vai dizer que eu sou tal eu sou o cara.

A: uhum.

OF4: ninguém vai dizer (como no passado) [00:30:22] como Roberto Carlos o cara sou eu. Não. Então a gente fica calado, só que quando eu cheguei lá nesse lugar é uma comunidade. O homem tava aos gritos dizendo que tava uma semana já, duas semana e:: então quando me permitiram pra ir olhar lá olhar lá a perna tava super grossa, já tava roxa.

A: fazia quanto tempo?

OF4: duas semana.

A: duas semanas.

OF4: exato.

A: qual foi a cobra?

OF4: surucuru.

A: esse que mordeu.

OF4: então com três dias morreu. Não tinha nada que fazer porque já tinha penetrado no corpo, tava totalmente já envenenado.

A: espalhou o veneno por todo corpo?

OF4: espalhou.

A: entendi. eu acho que é isso. Então o muito obrigado pela entrevista.

[00:31:29]

Entrevista Cuidador 5

Idade: 55 anos
Religião: Ordem da Santa Cruz (Cruzada)

Informações:

a. A entrevista foi realizada com o apoio de um intérprete para garantir a comunicação efetiva.

b. O pajé utiliza a medicina indígena para o tratamento de várias doenças e iniciou suas atividades xamânicas desde muito jovem. Relatou vários casos de acidentes ofídicos entre indígenas, de familiares seus inclusive, alguns destes morreram em decorrência do envenenamento.

c. Ao final, explicou que o pajé é visto na comunidade com temor, desconfiança e com preconceito. Nesse sentido, ele tem dificuldade para repassar o conhecimento tradicional para os mais jovens (filhos, netos) porque eles não querem ser associados com esse tipo de prática.

[00:00:04]

A: eu vou fazer perguntas aí você reforça pra ele em tikuna tá? Então vamos lá é:: o senhor já viu algum::.. alguma pessoa picada por cobra é:: e o quê que foi feito?

OF5: já, primeiro né assim... assim o::.. primeiro meu irmão a caçar né (...)

A: (...) tava caçando.

OF5: na restinga né... Aí quando coisa cobra né, andar a gente assim... andar, caçar né. A gente não usa bota né, aí feriu aí que morde.

A: qual cobra que era?

OF5: esse jararaca.

A: jararaca.

OF5: Daí tudo parece que tem né? Aí quando passar roçando, capinando aí quando peguei, morder também.

A: mordeu o seu irmão? E o quê que ele sentiu depois que a cobra mordeu?

OF5: Aí depois fazer coisa né. Tirar alguma coisa leite::... ele sente dor. (foi morrer né) [00:01:28] antigamente né pra curar a gente né...aí chama pajé né... aí curando, curando aí ficou melhor um pouquinho. Aí que meu pai me contou né. Vai buscar remédio para curar né (...)

A: (...) uhum

OF5: pra botar coisa né (...)

A: (...) Remédio do mato? (...)

OF5: (...) remédio do mato (...)

A: (...) e que remédio era esse?

OF5: leite de capimari (capinuri) Clarisia racemosa Ruiz & Pav., Moraceae.

A: (...) leite de capimari.

OF5: aham. Botar aí. Aí passou porque inchou né o pé do gente, ai calmou daí, parou... mas não comer alguma peixe remoso. Tem que comer peixe curimatã, sardinha. Se comer remoso aí continua de novo dor aí (...)

A: (...) dor volta?

OF5: volta.

A: Quais peixes que é remoso e que não pode comer?

OF5: Bodó, o::.. aquele:: pacú, Piratininga que é remoso. Mas bom curimatã, sardinha, (inint) [00:02:42] traíra, traíra é bom também.

A: e::.. e... bicho do mato pode comer?

OF5: não pode comer (...)

A: (...) o quê que não pode comer?

OF5: porque mordeu né. é... cobra né tem dente, depois da dor né de novo, porque no pode comer. Só peixe é::... comer tambaqui aí que melhor... bom comer.

A: esses aí são bons.

OF5: uhum.

A: E por quanto tempo a pessoa tem que ficar de resguardo?

OF5: Resguardo bem três mês.

A: (...) três meses para poder curar direito (...)

OF5: (...) pra curar direito. Pra ficar bom né. se não dá alguma coisa... coisa se chama (inint) [00:03:29]

A: (...) se não fica com tumor.

OF5: aí quando fica... cuida né. Aí sim (tora) (...)

A: (...) aí tem que torar. E o senhor conhece algumas pessoas indígena que tiveram que torar perna, torar braço por causa?

OF5: tem sim.

A: Em que situações? Conta aí algum relato que o senhor conhece?

OF5: aí que... ai que... quando sai (inint) [00:04;15] tem que agora levar no hospital. Aí lá que fica bom. (inint) [00:04:28]

A: o quê:: o quê que a::.. o quê que a pessoa tem que fazer para se proteger da... da cobra. Pra cobra não picar.

OF5: tem que usar bota pra trabalhar né, pra capinar, pra roçar, pra proteger né da cobra. Isso daí.

A: Pergunta para ele se tem algum tipo de proteção::... de...de algum de alguma coisa que a gente pode fazer pra aquela pessoa se proteger pra fechar o corpo?

OF5: se a gente... se a gente... o cara viu primeiro a cobra a gente tem que matar.

A: uhum.

OF5: essa daí que é a proteção pra gente. Se o cara não viu a cobra:: ai vai em cima do cara já. Morde mesmo. Só isso isso (inint) [00:05:24]

A: Não tem nenhum tipo de::... amuleto que eu posso usar pra me proteger além da bota ou algum tipo de planta que eu posso utilizar pra afugentar a cobra?

OF5: não.

A: normalmente quando a cobra pica a pessoa não tá perto de casa, tá longe trabalhando na roça, pescando, caçando, o que que deve fazer imediatamente que a cobra pica?

OF5: se o cara adoece quando::.. lá pelo mato quando o cara assim::.. no igapó assim...o cara tem que gritar os::.. pra outras pessoas pra buscar (...)

A: (...) pra pedir socorro

OF5: aí o cara foi pra lá, trazer o cara.

A: e aí quando chega na comunidade o que que é feito?

OF5: aí se não tem o pajé o cara levasse no posto. Às vezes o enfermeiro, enfermeiras faz encaminhamento e leva no hospital (...)

A: (...) no hospital. E quando ele vai no pajé o que que ele pode fazer pra melhorar essa pessoa até levar para o hospital? Pergunta pra ele.

OF5: o pajé tem que fazer um tratamento, faz um tabaco assim. O cara se faz soprar onde... no lugar (inint) [00:07:10] de cobra. Esse cara vai soprar, ai quando esse cara melhora, levado pro posto, aí o doutor avalia ele ou ela se não pudesse melhorar e levar no hospital. encaminhando pelo hospital

A: vai pro hospital pra tomar um soro?

OF5: uhum.

A: E aí quando ele volta pra comunidade tomou soro uns dias lá e voltou para comunidade. O pajé continua acompanhando ele?

OF5: Não, não precisa não (...)

A: (...) não precisa (...)

OF5: só uma vez.

A: só uma vez mesmo já resolve.

OF5: já resolve

A: em que situações, que outras situações pode é... agravar aquele acidente, aquela picada de cobra ? pra ele piorar que situações que coisas que podem::.. ele já relatou que é se comer comida remoso é uma das coisas. Tem mais outra coisa que não:: que ele deve evitar pra piorar? (...)

OF5: (...) piorar é?

A: isso para piorar.

OF5: Quando a mulher tá grávida não chega perto as pessoas que morrem (inint)[00:08:30] tem que longe. 5 minutos:::... 5 metros assim, o cara fica (...)

A: (...) se a mulher estiver grávida.

OF5: se a mulher perto dele já::.. o cara saiu sangue no dente dele, boca, as vezes nariz aqui o, aí ser pior::.. a mulher grávida.

A: que mais a mulher grávida o que mais?

OF5: se o homem tá, se o cara tá valente aí também não pode chegar perto do cara. Ficasse a mesma coisa assim se sai sangue no unha, nariz, boca.

A: aí agrava a situação?

OF5: uhum.

A: mulher menstruada também tem problema?

OF5: tem

A: tem problema. Entendi. E como que você como que vocês entendem? Por que que as pessoas são picadas por cobra? Por que somente porque elas estão expostas ou porque elas fizeram alguma coisa errada e foi picado também? como que é?

OF5: se o cara ficasse dentro da casa não pode picar porque aqui não tem... não tem cobra. Tem que ser pelo mato, pescar, fazer a roça, derrubar o pau assim, cara. Aí se encontra a cobra.

A: no caso quando se expõe no mato?

OF5: uhum.

A: existe um::.. uma outra explicação pra pessoa é ser picada de cobra além dessa tipo:: aquela pessoa não tá fazendo coisa boa na comunidade, ela pode merecer ser por cobra.

OF5: não

A: não. Além do tabaco e do leite da da::.. do leite da árvore que ele falou que pode fazer. Que outros tratamentos podem ser feitos tanto pro paciente tomar ou para colocar em cima da picada? Pergunta pra ele.

OF5: se não tem pajé nas comunidades, se a gente mata o cobra, raspa o coisa (...)

A: (...) raspa o quê?

OF5: o couro, amarra o carne aqui, o carne no lugar que o cara morde cobra (inint) [00:11:17]

A: eu amarro com o couro colocando ou a carne da cobra? (...)

OF5: (...) carne da cobra. Tem outra também é bom (inint) [00:11:42]

A: o próprio feu da cobra?

OF5:: uhum. Próprio daí

A: o quê que a::.. qual que é efeito de beneficio que dá::: o feu?

OF5: pra passar o dor (...)

A: (...) pra passar a dor.

OF5: uhum.

A: e quando eu coloco:: encosta a carne da cobra. O que é melhora (...)

OF5: (...) pra passar o dor também.

A: a carne vai fazer o quê?

OF5: Porque (é só uma carne das) [00:12:04] cobra mesmo.

A: Aí ela vai tirar o... veneno?

OF5: aham. Tirar o veneno. Isso.
A: vocês entendem que é importante é... quando ser picado ser atendido:: por mais que seja atendido pelo pajé ou fazer esses...esses...esses tratamentos com plantas, com leite de árvore, mas é importante também ir pro hospital?

OF5: importante (...)

A: (...) é importante.

OF5: uhum.

A: Por que que é importante?

OF5: porque... porque é:: as vez no hospital o próprio pra curar isso daí, o remédio (...)

A: (...) o antiveneno.

OF5: uhum.

A: Se... se vocês recebessem:: vocês continuam trabalhando né, pra roça, pescando, se você recebessem material pra se proteger melhor durante o trabalho como bota, peneira, roupa como aquela ali de manga cumprida vocês usariam sem problema?

A: Vocês gostariam de ter material pra... pra utilizar pra se proteger pra ir para roça?

OF5: pode ser, mas aqui (inint) [00:13:44] (...)

A: hoje em dia vocês vão pra roça? pode ir descalço, pode ir de bermuda. Se vocês recebessem orientação de como evitar andar com mais cuidado e também com calça comprida, com bota, com perneira, com alguma coisa assim que não te proteja totalmente, mas que vai reduzir o dano se você for picado vocês usariam ou vocês teriam dificuldade de usar?

OF5: usaria (...)

A: (...) usaria? hoje em dia vocês não usam porque não tem (...)

OF5: uhum

A: mas vocês usariam.

OF5: usaria.

A: Tem algum período que acontece mais picada de cobra? tempo do ano. Pede pra ele contar.

OF5: mês de abril.

A: mês de abril. Por que como que tá o clima no mês de abril?

OF5: Restingas né... por que o rio tá enchendo (...)

A: (...) o rio tá cheio aí tem as restingas::... e aí a cobra junta mais que reduz o tamanho da terra.

OF5: (...) junta mais... reduz isso.

A: O senhor já viu alguém ser picado de cobra e morrer?

OF5: Já.

A: já. Como foi? (...)

OF5: (...) família.

A: família. O que que era pro senhor?

OF5: meu tio. Ele morreu já.

A: dois tio morreu por picada de cobra.

OF5: tio né. doutor né. morreu já.

A: Porque não foi socorrido a tempo. Com quanto tempo depois que foi picado teu tio que morreu com quanto tempo depois de picado ele morreu? Quantos dias horas ou horas?

OF5: só um dia.

A: um dia. No outro dia morreu.

OF5: de manhã né. 5 horas (...)

A: uhum. No mesmo dia. (...)

OF5: (...) No mesmo dia.

A: e... e esses que morreram não tinha pajé pra atender?

OF5: (primeiro tem pajé né) [00:16:12] quando... antigamente não tinha pajé. (inint) [00:16:22]

A: (...) não tem mais jeito. A quanto tempo que o Senhor conhece que o senhor faz esse tipo de tratamento? Mais ou menos quanto tempo?

OF5: faz uma semana.

A: não, não. Quando ele começou. Explica pra ele. Hoje ele tem 55 anos com quantos anos de idade ele tinha quando ele começou a fazer pajelança, pra fazer tratamento pras pessoas?

OF5:Tem 8 anos, né. Eu primeiro... quando morreu um tio, né. Oito anos de idade, né. Morreu faz tempo.

A: e com quanto tempo ele começou a fazer tratamento pra pessoas ficar boa? Quanto anos tu tinha?

OF5: mais 14 ano.

A: 14 anos que começou. Quem te ensinou? Quem passou conhecimento pra ti?

OF5: minha família, outro tio né, outro tio velhinho né. (inint) [00:18:13] coisa (...)

A: (...) aprender

OF5: aprender o que vem de outro pajé né. pra ficar bom se não morrer frente né. Quando minha irmã, quando minha mulher doente família quando da

queda, cair no chão...nenenzinho...ai chama, vem para cá né, pessoal né...fazer tabaco, assopra... Depois do amanhã fica bom.

A: o senhor recebeu esse conhecimento do tio que antes de morrer ele passou para ti (...)

OF5: aham.

A: Como... como que o pajé ver o:: a cobra? é uma cobra é uma::... um bicho traiçoeiro, é um bicho inteligente, esperto. Como que:: qual que é o significado da cobra pros indígenas ticuna?

OF5: Dói no braço dele, né alguma coisa, né. bicho né. Que eu sabe. Tu sente assim.

A: tá, entendi, como que fica?

OF5: fica que (...)

A: Qual a importância da cobra pra::... tem alguma importância a cobra? como que vocês veem, vocês têm medo da cobra, você respeita a cobra? Como que... como que é visto entre os tikunas. Qual que é a percepção?

OF5: Entre nós tikuna, a cobra mesmo tem nem (...)

A: (...) significado importante.

OF5: significado porque cobra não é amigo da gente

A: sim.

OF5: Por isso que quando...quando a cobra a gente ver a mata logo.

A: matar a cobra.

OF5: uhum. Mata logo.

A: Quando as pessoas são picadas por cobra. Qual que é a::... a reação de vocês? é tipo correr da cobra ou matar cobra? é importante matar cobra se por picado?

OF5: aham. Matar.

A: Por que que é importante?

OF5: Porque que a::.. não porque a cobra quando a gente morde a cobra morde a gente, mata logo pra não ser porque a cobra não é amigo da gente. É inimiga. Tem que matar.

A: é algo que trás perigo?

OF5: uhum. Trás perigo.

A: entendi. Então eu acho que é isso, muito obrigado vou encerrar.

[00:00:02] áudio 5B

A: Pergunta para ele é::... onde que encontra essa:: essas plantas que faz o tratamento?

OF5: ele tem...ele tem planta aqui.

A: ele tem planta aqui perto da casa da mesmo?

OF5: aham.

A: então tem perto que é para proteger?

OF5: aham. Sim.

A: qual que é?

OF5: (inint) [00:00:47]

A: todas essas plantas que estão aqui em volta são plantas que servem pra fazer algum tipo de tratamento?

OF5: aham.

A: e essas plantas foi plantada aí ou já nasceram da selva mesmo aqui?

OF5: são daqui mesmo

A: daqui mesmo?

OF5: uhum.

A: Pergunta pra ele se:: ele já tá trabalhando alguma outra pessoa pra também receber os conhecimentos que ele tem?

OF5: já.

A: outra pessoa. Quem que é?

OF5: tinha um::.. tinha um cara que mora aqui em frente... o cara já ele que dá::..a mais::.. a mais inteligência pra ele porque já morreu já.

A: Ah tá não do que ele recebeu. Mas eu quero saber se ele tá passando os conhecimentos que ele tem pra uma outra pessoa mais nova, uma criança por exemplo pra ficar conhecimento por mais tempo.

OF5: nenhuma pessoa.

A: nenhuma pessoa, mas por que ele não quer passar ou porque ele ainda não escolheu ou alguém não tem interesse ainda?

OF5: ele não quer não.

A: ele não quer não (...)

OF5: (...) ele não quer não. já está aí o que é pessoa vem fazer alguma coisa né falando essa pessoa tá falando assim, porque outro sabe pra matar outro gente né. ele não gosta não, pessoa tem que trabalhar bem né como você né que trabalha bem pra curar gente e ficar bom. Se fazer alguma besteira por aí matar outra pessoa:: pessoa também mata, fazer alguma coisa né.

A: entendi.

OF5: (inint) [00:03:36] Fazer alguma coisa.

A: tem que usar para o bem?

OF5: isso.

A: e os filhos dele não querem pra comunidade não confundir eles podem ser chamado de Feiticeiro::.. (...)

OF5: (...) não querem. Isso (...)

A: então tem um preconceito ter esses conhecimentos::.. é confundido como alguém que pode fazer o mal?

OF5: aham. Pode.

A: entendi. Então é por isso (...)

OF5: (...) que o cara não pode passar pra outras pessoas.

A: Ah entendi, ah é::.. não pode passar pra qualquer pessoa porque a pessoa... ninguém não tem controle se ela usar para o bem ou para o mal.

OF5: uhum. Isso.

A: entendi. então aí (...)

OF5: por que assim minha família né::.. minha mulher, ela não quer mais, mas pessoa aqui né não pode receber (inint) [00:04:31] família alguma coisa doente né que vem para cá (inint) [00:04:38] né pa curativo fazer bem, rezar se pegar uma coisa, né doente, rezar e (prever) [00:04:50] rezador né. reza, reza e fica melhor.

A:Pergunta pra ele se ele é muito procurado todo dia, como é que é?

OF5: sim.

A: pergunta pra ele se::.. pra que mais doença que procura se chega até pra ele tratar.

OF5: porque antes era primeiro as crianças::... pra curar mais... outro dia eles mermo voltaram pra ficar bom de novo.

A: entendi. Ele tem um:: um nome indígena?

OF5: chamam na língua indígena de (inint) [00:06:59]

A: e::.. ele pertence a algum clã de animal por exemplo? Na família dele.

OF5: mutum.

A: ah mutum. Como fala mutum em tikuna?

[00:06:12]

Entrevista Cuidador 6

Idade: 82 anos
Religião: Ordem da Santa Cruz (Cruzada)

Informações:

a. A filha e a neta acompanharam integralmente a entrevista, auxiliando a relembrar alguns relatos de acidentes ofídicos.

b. O entrevistado se define como xamã, recebeu o dom de cura dado por Deus.

c. Trabalha com 3 espíritos e destacou o uso de ambé, cubiu e outras plantas medicinais disponíveis na natureza.

d. O entrevistado relata ainda que foi seringueiro por 36 anos e foi picado por jararaca várias vezes.

[00:00:01]

OF6: (gente) [00:00:01] naquele tempo. É... bruxeiro era o matador de gente, sacaqueiro era outro matava::... curava e matava gente, eles curava mas também matava os pobre.

A: E o pajé? Como que é?

OF6: O pajé... o pajé ele curava e matava também. Pajé, ele curava e matava:: na brincadeira é só ele olhar pro senhor. O senhor tava com uma roupa dessa podia se aprontar [00:00:24] que no outro dia você já estava morto. Naquele tempo era a gente, quase bem nu bem dizer né?

A: Sei.

OF6:Com aquela roupinha. Eu não trouxe minha roupa se não ia mostrar pro senhor.

A: Eu já vi sua foto com a roupa.

OF6: é Já?

A: Já. Lá na::.. do evento da CESAI. Seu Anísio, é::... para gente começar a conversa, como que o senhor começou a ter esse dom e fazer os trabalhos pra curar as pessoas?

OF6: (inint) [00:00:54] Irmão esse (dom) parece que foi dado por Deus, né? Eu nunca fui aprendista de nenhuma pessoa, de nenhum os curadores, pajé, nem ninguém né. Esse dom já foi Deus me deu né? Então eu comecei quando era pequeno, mãe, minha mãe conta né? Caía, dava aquele tremor, morria, querendo morrer, não morria, mas porque já veio com aquele dom que Deus deu né? Eu criei, fui criando e tô aqui né. Sofri muito mano, minha mãe e meu pai sofreu muito comigo. Tem hora que ele levava como o senhor tá dizendo, com o pajé.

A: uhum.

OF6: os pajé dizia assim para ele: ah meu pai se chamava Ciriaco: Seu Ciriaco nós não pode tirar né? porque esse já veio dom de nascença, Deus quer dizer (inint) [00:01:56] deuses na idioma português né.

A: uhum.

OF6: Dizia olha seu Ciriaco ~~aí quer~~ (idioma indígena). Deus deu ninguém pode tirar. Então fiquei criando, fui criando, pelejando a vida. Qualquer coisa que eu tinha era queda parece que ia morrer de uma vez, mas não morria né. Porque o espírito que Deus me deu né, se invocava como diz eu não... eu não tava pronto para isso né.

A: Mas protegia

OF6: protegia, protegia e eu era menino, não aguentava, eu fui começar a trabalhar com
Trin::... com 32 anos.

A: Para fazer os trabalho como xamã

OF6: como xamante. Então aí:: aí eu trabalhava com três espíritos ne:: do fundo que é o boto. Ele é bicho mas o espírito dele é como Cristão né.

A: uhum.

OF6: Mas ele é bicho:: bicho do mato trabalhava. espírito do ar que hoje eu trabalho:: Quando comecei trabalhar eu trabalhava com esses três, então depois que esse velhinho aí apareceu...irmão José, não sei se ouviu falar.

A: Sim, conheço, da história dele.

OF6: é. Taí ele aí oh. Depois que ele passou (...)

A: (...) O senhor é da Santa Cruz?

OF6:Eu era aí. Agora eu tô assim como:: um cinturão de soldado né.
A: O senhor já foi mais ativo na Santa Cruz hoje nem tanto

OF6: (inint) [00:03:42]

OF6: Eu passei 20 anos como diretor da comunidade.

A: Lá de São Joaquim.

OF6:Lá de São Joaquim. Eu só deixei o velhinho quando ele deixou nós também né.

A: entendi.

OF6: Eu trabalhei do tempo que ele chegou, eu trabalhei também.

A: O seu Anísio, o senhor durante a sua vida e seus 82 anos, todo tempo cê tem trabalhado
aí como xamã pra ajudar as pessoas.

OF6: é.

A: O Senhor... o senhor tem visto, poderia me relatar casos de pessoas que foram picadas por cobra e que senhor tratou essas pessoas?

OF6: Irmão até eu mesmo fui picado 7 vezes.

A: 7 vezes por cobra.

OF6: Jararaca.

A: Por jararaca. E aí que...que aconteceu me conte aí, como é que foi? O quê que o senhor tava fazendo?

OF6: Eu era Seringueiro. Eu trabalhei 36 anos... Cortei seringa, né, 36 anos. Olha eu trabalhava na estrada, eu com meu irmão mais velho. Eu comecei trabalhar

borracha, seringa com 6 anos. 6 anos eu tinha 8 anos quando comecei a trabalhar, meu irmão tinha 10 anos ele era mais velho dois anos.

A: E o senhor tinha quantos anos?

OF6: 8 anos.

A: 8 anos. Já cortava seringa na cidade?

OF6: Já cortava seringa.

A: Onde era?

OF6: (inint) [00:05:09] chamado capintuba.

A: Capintuba.

OF6: é. Capintuba eu trabalhei 9 anos, só nessa colocação. Trabalhei 9 anos eu com esse meu irmão. Quando nasci ele tinha 10 anos e eu tinha 8 anos. Papai deixou a estrada para nós cortar né, ele ia todo tempo cortando... não podia fazer roça. Como ele viu que nós já dava conta na estrada a gente nós deixou pra cortar e ele foi fazer roça.

A: Aí foi nessa época que o senhor foi picado por cobra?

OF6: Foi nessa época que eu fui

A: a primeira vez?

OF6:é.

A: o senhor tinha quantos anos?

OF6: Eu tinha mais ou menos uns 14 anos.

A: 14 anos. E como é que foi? O senhor estava cortando seringa?

OF6: cortando seringa na mata, né?

A: E aí?

OF6: E aí... eu com meu irmão... que nós não se deixava né! quando tava no centro é cortando aí, cheguei lá...sabe que lá chamava assim uma seringa... lá

por exemplo como ali lá na... na... na.. rua né. Daqui gente ia lá e voltava. As vezes tinha 2 seringueira, três seringueira... ia e voltava... nós chamava manga. Aí ele disse: maninho vai cortar essa manga... vou lá na frente. Tá bem! Aí eu fui embora era só água mano, baixo... baixo... baixo… que você não pisava em terra não.
Só o chavascal, buritizal (...)

A: (...) E foi lá que a cobra lhe picou?

OF6: Foi lá.

OF6A: Era terra firme ou era várzea?

OF6: Não! Várzea.

A: E o que que você sentiu quando a cobra picou?

OF6: Ah mano quando eu botei o pé assim aqui em cima de um montezinho que a gente pisava parece que ela tava enroscada. Quando ela... eu pisei assim...eu vi a porrada... Pah! Sai... Era desse tamanho assim... ia nadando em cima d'água. E lá jabuti meu irmão:: o senhor conhece o jabuti?

A:Sim.

OF6: Aqui e acolá era caminho chega ia machucado bonito. Nós só pegava aquele que estava no caminho mesmo, botava pau... deixava aí e nós ia embora.

A: E o que o você sentiu quando a cobra picou?
OF6: Pera aí. Que aí::.. aí eu gritei pro meu irmão né, ele tava já encostando assim no caminho direito, né? Eu fui com aquela manha. Ei maninho, ei maninho, mas eu não disse que cobra tinha me mordido. Porque quando nós olhava jabuti assim um chamava outro pra olhar né. Ele disse: - parece que achou outro jabuti e correu pra lá. Cadê seu mano? Aí eu disse pra ele: ah maninho tu nem sabe. O que foi? cobra me mordeu. Poxa vida. Cadê a cobra? Sacudi com meu pé que ele caiu, tava no pé... foi lá e foi pra cá nadando que só é água né. Só tem aquelas bola de pau, tronco de pau aquele só puro água. E ficou pra lá, ele pegou um pau: rum, sabe de uma coisa meu irmão cobra não se percura (procura) e nem onça. Vamo cuidar da nossa vida que é. Aí *fumo* embora. *cheguemo* lá aonde deixava balde. A estrada é assim: dá volta, bem aqui, aqui a gente deixa balde e segue cortando por aqui. Até chegar aqui. Quando é aqui te deixa o balde pra colher
e pega o balde e colhendo de novo. Aí eu disse: olha eu na minha mente como Deus é bom meu irmão. Eu tenho um::.. um bem dizer experiência do que Deus

ia dar...lá no monte longe era 3 hora andando na... no vara... na... estrada. O que é maninho? rapaz, vai cortar isso aí. Tu conhece aquele que chama ambé?

Vide cipó ambé
https://www.youtube.com/watch?v=i6J9pM4Sc8s

A: uhum.

OF6: daquele espinhento né que a gente faz paneiro ~~(panelinha)~~. Eu fazia um monte desses e ainda faço as *vez*. Eu disse pra ele: maninho vai tirar aquele ambé ali que sabe se não presta né. Como Deus é pai né. Como? nesse daí. Corta desse tamanho assim, uma braça em cima e corta aqui embaixo. Ele cortou, descasca ele, que aquela casca é espinhenta né.

A: uhum.

OF6: descascou, agora a tigelinha era desse tamanho, chamavam cabeça de macaco, aquelas tigela grande né. Raspou... raspou todinho aquela carne depois de cascar
aquela espinha dele. Aí ele pegou botou:: bota água aí dentro, água era bem ai logo, a água do baixo. Espremeu, espremeu...aí toma, me dá aqui que vou tomar. Já tava latejando. Puxa vida! Tinha me mordido bem aqui oh.

A: No pé.

OF6: No pé. Aí eu tomei, aí ele disse: olha, vamo amarrar essa borra aqui (se referindo ao cipó macerado)[00:10:10]. Amarra essa borra que foi machucada né.

A: uhum.

OF6: ele amarrou.

A: em cima.

OF6: em cima. Ele disse: ai seu mano parou que foi parece uma coisa que::.. que não
tinha acontecido...tava adormecido. Eu disse: irmão vamo embora cortar, bora cortar. eu disse: não. Bora. Eu disse bora.

A: O que cortar o que? o local?

OF6:Seringa

A: Ah a seringa

OF6:A seringa até chegar na boca da volta a estrada bem aonde deixa balde bem dizer. Aí não, *vumbora,* ai po, ficou adormecido, antes que pode ser que dói e era longe. Era umas 3 horas para chegar lá no tapiri. Aí nós viemo embora, cheguemo era umas 10 horas do dia lá no Tapiri. E meu pai tava lá né, minha mãe tudo morava pra lá na beira do lago. Que foi? cobra me mordeu. tá doendo? Eu digo: não. E no meio de viagem nós já tinha deixado três jabuti, jabota daqueles enormes. Eu o vi os velho conversando assim como estamos conversando né, jabuti é muito bom fígado da carne pra mordida de cobra também né.

A: Fígado de jabuti?

OF6: é, fígado de jabuti. Aí eu acho que eu escutava os velho antigo conversando assim conversando fora a gente sabe que quando aquele tempo ninguém ia escutar a criança. O Curumim queria ser lá onde tava ele conversando porque ia logo pega peia, mas hoje né nós só de longe assim escutamo (inint) [00:11:53] o::.. sou filho de Jabuti, o fígado. Oh a carne era boa.
A: Hum.

OF6: Eu disse: - papai quebra um jabuti desse. (Como eu já sabia né!?) E vamo comer.
-Ah meu filho não vai... Eu... quebra...eu que to mandando pai. Cuidado meu filho. Não, eu quero comer, eu quero morrer de barriga cheia e com o jabuti.

OF6A: tava desejando já.

OF6: tava desejando mesmo, mas eu já sabia que (...)

A: (...) Que era remédio.

OF6: que era remédio né. Eu duvidava mais porque já sabia que aquilo servia pra
remédio. Aí o velho não duvidou. Meu irmão era::.. uma jabota tinha bem oito ovos. Cascão né? eu disse pra mamãe: mãe bota isso na panela pra mim comer já enquanto a carne tá amolecendo eu vou comer ovo e fígado assado. Ah meu filho tu vai morrer. Que se morrer, eu morre de barriga cheia. A velha não duvidou. Eu tinha três outra irmã filha. Não é essa daqui que é minha filha. aí a minha irmã era duas solteira que tinha aí mocinha botaram lá na panela, a velha botou o fogo pra pra esquentar, pra pelar o jabuti eu só no ovo... eu com meu irmão, meu pai, digo égua aí aquele ficou adormecido só.

A: o pé continuava adormecido ou doía?

OF6: Só adormecido. Não doeu nada. Pronto quando foi de manhã tava bonzinho. Nem inchou, nem botou sangue, até hoje sete cobra que seis cabra que me mordeu não tem um saiu de dente nem dos olhos nem de nada.

A: Dentre... dentre todas essas vezes que as cobras lhe picaram teve alguma delas que foi mais grave que o senhor teve sintomas mais fortes? Não?

OF6:Depois disso só teve um. Foi o primeiro né. Quando eu estava com oito com::.. com
três ano. Eu era menino de que me alembro, né? Esse aí quase me mata.

A: Hum.

OF6: mas também eu era... era pequeno e não sabia como é o que era. Sabia que era dor mas não sabia que remédio era, né. Esse quase... inchou até aqui. Mas aquele foi o outro curador que me rezou, né?

A: O que que ele::.. o que que ele fez?

OF6: Ele rezava e.. e passava e:: não sei que folha que ele passava que eu tava nas ultimas né? (...)

OF6A (...) era criança.

A: Era criancinha.

OF6: Aí ele rezava e passava aquela folha. Pronto! Com uma semana fiquei bonzinho. Único que foi. Daí resto mais nada que toda que me mordia eu mesmo me tratava.

OF6A: É a mesma formiga ferra.

A: Esse é o Anísio e depois que o senhor já começou a trabalhar pra curar as pessoas
como chama? Como que o senhor tratava das pessoas que eram picada de cobra? Como que o senhor cuidava dela esse tra::..?

OF6: Olha.. eu, com esse que eu dizendo com ambé.
A: Uhum.

OF6:Me cura... me curei com isso. Eu também me curei porque eu... eu tiro, faço assim porque eu já sei que serve (...)

A: (...) o senhor já experimentou.

OF6: Já experimentei que que serve pra isso.

A: Uhum.
OF6: Eu não vou contar é que foi eu fiz pro fulano não. Pra mim servia, que servia
pra todos também.

A: e o senhor era procurado pra:: pra essa situação? (...)

OF6: (...)Pra fazer esse remédio, né? E outra irmão, tu conhece esse, como é? Cubiu?

A: Cubiu, conheço.

OF6: Pois é. Folha de cubiu também é bater e ver.

A: (...) o que que a gente faz?

Voz feminina (inint) [00:15:38]

A: a sua filha já foi picada de cobra?

OF6:Foi. Aí pega três folha que a folha é grande né? Pega três folha e soca.
Bem socada né. Aí espreme num pano aquele:: aquela folha bem socada, bota um pouquinho de água e bota uma colher de... de açúcar, bate, toma e aquela borra amarra.

A: Amarra em cima de onde foi picado?(...)

OF6: (...) amarra na tesoura aí ele puxa tudinho e o veneno.

A: Ele suga?

OF6: Ele suga o veneno que ele está ali suga tudo.

A: Seu Anísio o senhor poderia contar pra mim como que foi a história da picada de
cobra da sua filha?

VozFem: vamos ver se ele se lembra.

OF6: tô me alembrando, né?

A: Quantos anos cê tinha? A senhora?

Vozfem: Eu tinha.:: (...)

OF6: (...) Esse era.

Vozfem: Eu tinha dezesseis anos. Eu tava grávida do... do meu primeiro filho quando eu casei com meu marido. A gente fazia farinha lá no centro, né? E eu vinha voltando, né. Era seis hora. Tudo:: meus filho eu não tinha, era o meu primeiro filho. Aí eu vinha voltando ela me picou bem aqui.

A: No pé direito.

VozFem: E eu vim gritando (...)

A: (...) Que cobra é era?

VozFem: Eu estava grávida, uma jararaca. Seis horas.

A: Você estava grávida era seis da tarde?

VozFem: Seis da tarde cheguei a voltar.

A: E o que que a senhora sentiu?

VozFem: Aí eu vinha voltando, gritando com tanta dor. E eu estava com oito meses do meu filho, primeiro filho que eu tinha. Aí eu cheguei em casa a gente morava lá no na.. na casa da tia dele, quando eu cheguei eu não aguentava mais e eu falei pra ele: vai chamar o papai. E eu acho que eu vou morrer. Tava sangrando pelo nariz, pela boca, pelo ouvido:: aí eu falei pra ele: vai chamar o papai que agora eu vou morrer. Não aguentava mais de dor. Aí ele foi embora chamar ele. Quando ele chegou eu:: eu não me lembro quando que ele chegou (...)

A: (...) E você lembra o que que ele fez de tratamento? (...)

VozFem: (...) Aí quando eu me acordei eu tava toda amarrada com folha de cubiu. Ele me deu o sumo, eu o meu marido falou que ele me deu sumo do:.. da

folha do cubiu. Aí ele emplastou no meu pé. Ele disse: minha filha, não vai inchar não e não vai doer mais, parou o sangue e parou tudo. Saía sangue até do meu nariz, do meu ouvido. Aí eu pensei eu disse: ai meu Deus papai eu vou perder minha::.. meu bebê. Ele disse: tenha fé em Deus que tu não vai perder minha filha. Pronto aí fui calmando tudo, não doeu. Não inchou minha perna (...)

OF6: (...) Não incha nada.

VozFem: Ele rezou, rezou, rezou, aí ele disse: só minha filha que amanhã tu não vai mais pra roça. Passa um dia na tua casa. Aí eu disse: e amanhã de manhã eu venho, aí de
manhã ele foi lá e levou um monte de folha de novo de cubiu. Ele bateu bem batido, aí tirou o sumo com a mão dele, espremeu, aí ele me deu. Pronto não senti mais
nada, não perdi meu bebê graças a Deus. Com a folha do cubiu ele deixa emplastado no
meu pé e era uma jararaca grandona. Meu marido ainda matou ainda.

A: Entendi.

VozFem: Não senti nada. Graças a Deus. Eu hoje né fui contando a história.

A: Tá bom. Obrigado. E seu... seu Anísio o que que as pessoas que são picadas de
cobra devem evitar pra não fazer pra que não piore aquela picada de cobra?

OF6: olha:: na.. na minha mente que eu meio tiro por mim né?

A: Uhum.

OF6: Com a primeira logo [00:19:50] se você quer um irmão, colega, o amigo picado de cobra. Faz favor de nenhuma mulher que tá gestante olhar. (inint) [00:20:02]pau na cabeça. Quase que eu morri uma vez.

A: O que que acontece?

OF6: Acontece que aí vai latejar, vai botar sangue por todo lado. Pé, da venta, dos olho do ouvido. Por aonde tem o lugarzinho ferido sai sangue (...)

VozFem: (...) pode até matar, né?

OF6: capaz de até matar. Esse é perigoso. Onde é que Deus olhei se eu souber que uma... uma outra picada de cobra, pois essa casa está aqui. Se quer uma um:: um paciente mordido de cobra lá na frente tem um, não vai pra lá que tá picada de cobra e às vezes tem... tem gente e não né precisa nem tá gestante. Só na conversa, porque eu:: a gente sente, porque já aconteceu comigo.

A: Por que que umas pessoas são prejudiciais pra? (...)

OF6: Porque muitos quer dizer tem um coração mal.

A: Uhum.

OF6: Né? Tem um pensamento mal. Então isso quando eu tenho ela aí ele volta com uma
que tá uma picada de cobra é que ela dá choque no... na... no paciente.

A: Aí a gente tem que evitar essas pessoas por perto. E por quanto tempo que a gente tem que evitar?

OF6:Ah até fica bom.

A: E normalmente uma pessoa de picada de cobra fica bom com quanto tempo?

OF6:Eu:: tinha vez que era só dois dias três dia. Eu:: Agora os outros eu não posso dar testemunho que eu não sei.

A: E os outros que o senhor tratava quanto que o senhor recomendava pra ele ficar?

OF6: Eu fazia assim pra ele, né? Por exemplo, não tenho mais doído, então amanhã já
pode tá dentro de casa, né? Mas como eu falava: cuidado que num conversando com os outros e nem olhar mulher buchuda, prenha por aí. Aí ficava guardado pronto. Passava dias ficava bom! [00:21:53] e agora? agora de muito tempo pra cá eu não rezo mais, nem faça remédio porque quando cobra morde aí já la do mato já leva pro hospital e não se alembra de rezador, de remédio caseiro, não quer, vão logo pro hospital eu não posso fazer nada, né?

A: Entendi.

OF6:Então mas é que eu sei, sei remédio, eu sei rezar pra isso também né? Graças a

Deus. Deus deu esse dom, até pra isso. Quando não tem remédio eu rezo também, é mesmo que está botando o remédio no paciente.

A: Então o seu tratamento pra quem é picado de cobra é tanto com as plantas, como a
folha do cubiu como o senhor já falou e também com as ora... com a reza. (...)

OF6: (...) reza. E com a reza.

A: Com a reza. Entendi.

OF6: tinha um senhor que era lá passou oito dia no hospital. Inchou a perna tava da cor dessa calça a perna dele. Estava desse tamanho. Já tava (tomo)[00:23:02] quase tumorizando. Aí foram lá comigo: - irmão que tal: era o Fifi, tu conheceu o Fifi né? Que era o tio do teu marido né?

VozFem: Aham.

OF6: Chamava Manel mas o apelido dele era Fifi e rapaz aquele homem gritava gritava, gritava a perna dele tava dessa cor ai, preto. Tava inchada desse tamanho assim. Aí ele era meu compadre já estava com oito dia no hospital e não aguentava mais aí:: aí ele pediu
pra doutor que saísse de lá. Aí o doutor deu alta pra ele. Também depois que ele
saiu, na hora ele saiu, as filhas dele foi lá comigo. Chamava só tio. Titio, papai tá passando mal lá na casa, saiu hoje do hospital ele mandou nós vim aqui com o senhor. Bora, embarquemo e fomos lá. Mano, o homem tava: ai, ai, ai. não podia rolar que a perna dele tava desse tamanho. Não foi fácil não. Eu rezei umas sete horas. Quando foi dez horas eu rezei de novo. Meio dia eu rezei, pronto! Calmou. Outro dia já que ele chegava tava murchando. Graças a Deus. Me dá o remédio. Aí eu dava esse remédio que tem. E
esse capim da beira do rio esse capim espinhento (...)

A: (...) uhum.

OF6: também é muito bom.

A: aquele que corta?

OF6:É esse que tem (...)

A: (...) Não é o Tiririca não?

OF6: Não. Esse capim da beira do rio.

A: Esse que o gado come. O quê que é feito com ele (...)

OF6: (...) É. Só o que tem na beira do Solimões demais isso aí.

A: Uhum.

OF6: Pode tirar a folha dele do mesmo jeito. Soca que Deus o livre é o rei que não tem
aonde tem esse ambé, não tem cubiu, soca aí sair muito assim tira o sumo e toma.
Pronto irmão. Não tem outro remédio também. Deus abençoe. Amém. E também né
tem várias remédio né?
A: pode ir contando. Eu tô:: tô disposto a ouvir.

OF6: Então isso é que que tem né? Que eu sei. Ambé, cubiu, os capim do beira do rio.

A: uhum.

OF6: E açaizinho aqui, açaí desse tamanho também. Bom a beça. Uma vez (...)

A: (...) o que que é?

OF6: É açaí. Filho de é o açaí.

A: ah, o açaí. o novo.

OF6: no rio que ele está desse tamanho assim?

A: Uhum.

OF6: Corta ele bem onde está o fim do duro, né?
A: Sim.

OF6: Que vai, vai crescendo vai, vai descascando aí. Corta bem no fim onde está o último a casca ou a folha, né? Aí vai descascando até chegar no fim da daí bate. Aquele também eu já fiquei bom. Eu tiro por mim porque eu faço o que é::.. eu sei que é. É bom.
Aí eu estava curando dois paciente. Em dois em dois dias iam lá comigo pegar remédio que eu ensinava né?

A: Todos os dois picado de cobra.

OF6: Não. Paciente de de doença mesmo.

A: Entendi.

OF6: É. Aí eu saí com a bolsa de sacola já tinha tirado todas que estavam, faltava um e aonde tirava era uma bolinha de cerrado assim. Era uma bolinha de cerrado. Parece que o desgraçado disse é aqui que tu gosta de tirar remédio, hoje tu vai pegar o teu. Eu já tinha tirado muito... todas folha. Quando eu fui meter a mão ele pulou rapaz. Não enxerguei que eu entrei ele pulou bem aqui. Foi tchac. Puxei a mão. Olha a grossurona.

A: era uma cobra

OF6: jararacona dessa grossura aqui. quase uma braça.

A: essa picou na sua mão?

OF6: isso, isso, que picou na minha mão aí eu o paciente estava todas duas já estava em pé na ribanceira assim a ribanceira. Aí eu olhar. Que foi seu Anisio? Rapaz ele traz um pau e vem matar essa cobra e já me mordeu. Pronto aí correram
melhor correr pra matar e quando correram lá pra casa já. Aí eu subi. Subi, olha rapaz já estava latejando aí (...)

A: (...) doeu muito

OF6: doía. Doeu, mas sabe que quando pica logo dói né?

A: Uhum.

OF6: Pra mim e pra outros também dói. Aí ele: olha a cobra já mordeu seu Anísio. Era os dois paciente. Tudo ia lá comigo eu ia ele ia pegar remédio que ensinava e só lá que tinha. Outros canto não tinha. E lá eles iam pegar remédio. Era até dia domingo. Aí correram aí cadê a cobra? olha vocês vão matar essa cobra tá bem nessa boladinha de cerrado. Rum. Nem meus filho, nem genro [00:27:53], nem os dois paciente vão matar essa cobra. Aí tinha um rapazinho que é meu neto: vai buscar dois traçado rapaz se eu quero isso eu
não era da... da... da esquerda eu peguei terçado só era uma boladinha onde eu tirava o remédio. Vai daí e eu vou daqui. É parece que ele ficou esperando de novo. Aí o menino veio de lá:: ela vinha danada eu só tchá. Torei a cabeça,

peguei um pau, levei olha vocês não queria matar olha eu já matei essa cobra que me mordeu. Aí que foram olha o tamanho dessa jararaca.

A: E normalmente o que que a gente deve fazer com a cobra quando a gente vê ela pelo ambiente?

OF6: Que:: ela mata::.. assim pode matar.

A: O ideal é matar?

OF6:Pode matar que ele::.. não vai deixar uma cobra viva né? eu nunca deixei essa:: desde quando ele corre de mim que eu não posso matar [00:28:51] mas eu podendo matar, não escapa um desgraçadíssimo.

A: E como que a gente as pessoas devem podem se proteger de picada de cobra?

OF6: É só com Deus mesmo, pede a Deus.

A: é.

OF6: Só Jesus que pode sabe? O passado que ele está passando ele está olhando. Tudo é dado por Deus. tudo é Deus que dá dor. Tudo Deus (...)

A: Tem alguma forma de oração pra fechar o corpo alguma coisa assim? o senhor conhece, trabalha com algum tipo de coisa assim?

OF6: Eu trabalho.

A: É.

OF6: eu trabalho pra... pra não pra tudo, não posso dizer que só quem sabe é Deus, né? Mas o que eu conhecer:: que os:: os espírito que incorpora em mim né? Eles se conhecem. Eu não posso conhecer. Né? Mas é um espírito que conhece que é a:: que por exemplo:
O senhor é o xamante, né?
A: Uhum.

OF6: Você ir lá incorpora o espírito anunciou aí o senhor fica como que fosse um morto, né? Esse é o espírito que coloca, que incorpora no senhor aquele que vai mostrar (...)

A: (...) eu só sou mediador.

OF6: Só mediador.

A: Do que ele vai propor tratamento.

OF6: tratamento. Aí que João falou que é doença o que é que cura né? Você não acredita? Sim senhor acreditar ou não? Eu vou dizer pro senhor.

A: Pode falar.

OF6: Por quê? Eu já fui pra tudo canto. Até Rio de Janeiro, trabalhei um mês de São Paulo trabalhando em curativo lá, no Rio de Janeiro e passei um mês também. Agora
passei pouco tempo em Brasília. É. E só. Mas com o poder de Deus irmão. Aqui em São Paulo, aqui taba::.. em São Paulo maioria dos dois doutor me conhece lá também
né? E agora tamos está dando valor porque já viram que o remédio de brincadeira né
então é só lá agora aqui ainda não falei com o doutor com nenhuma autoridade daqui
prefeito, vereador não, mas lá em São Paulo todos eles me conhece o doutor. Quando em
São Paulo do Sul, no Rio de Janeiro os doutor me acolheram e meu irmão comem mesmo que é assim que dizem assim o seu doutor. Ela me chamou de doutor índio, né? Eu sou índio mesmo não vou dizer que eu sou filho de americano, espanhol, de inglês
não. Eu sou Kukama do solimões e vou morrer e como kukama. (inint)[00:31:33] Aí olha os branco depois que os branco foi conhecer hum aí me acolheram bem. Olha irmão um pobre de mim como eu. Você faz um ovo com canudo. Eu digo pra tua (inint) [00:31:53] e tudo me conhece que eu não eu sou o pobre mais analfabeto que tem né? Mas como o doutor disse: analfabeto na carne mas no espírito o senhor não é? Pois doutor falaram pra mim né? Digo é verdade. O que o senhor sabe hoje doutor de São Paulo, do Rio de Janeiro. Você é analfabeto na é carne, mas no espírito que se une analfabeto o que o senhor sabe ninguém sabe. E o que eu sei o senhor não sabe. Né? Quando eu peguei o doutor juiz, promotor, devogado, delegado, prefeito. Dois doutor, duas enfermeira, mano quase morre de desgosto e eu vou me prender. Vão me prender. O pobre analfabeto que não
sabe nem o que eu vou com canudo e arranha. Eu morava na casa do doutor Gênesis. Né? Doutor, tu compra tu sabe como eu vou contar pra você levar e gravar se está gravando Diga pra tudo se percurar meu nome em São Paulo é aí da onde comecei trabalhar acreditar e que Deus mostrou isso aí São Paulo do Sul. Pode percurar::.. São Paulo do Rio de Janeiro percura o índio que chamava

Anísio Morais dos Santo Índio do alto Solimões que o senhor vai encontrar no que você já está no Instanet lá né? Pois doutor de lá me apoiaram muito.

A: Hum. Seu Anísio e o senhor... o senhor acha que a pessoa que é picada por cobra ela também deve ir pro hospital ou não?

OF6: Se tivesse remédio não. É. Se tiver remédio ou se quer acreditar no rezador como como eu bem dizer né?

A: Uhum.

OF6:Como eu. Mas eu não estou dizendo que quando é uma picada de cabra eu vou logo pro hospital não quer me lembra de mim, não lembra de mim, de remédio caseiro, né?

A: Mas antigamente eles procuravam.

Procurava irmão né.

A: quando o hospital não era tão acessível.

OF6: É não era fácil aí percurava, mas agora não. É duas coisa: eles não lhe percura.

A: Hum.

OF6: É pra mulher ter criança e picada de cobra. Esse daí esqueceram de mim que não sabe nem se existe.

VozFem: Ei pai, o senhor tem que se lembrar da... da surucucu que mordeu o tio Joaquim, ele tem a perna curta, um lado e a outra normal foi o senhor mesmo que fiz o remédio da surucucu.

A: Conta aí esse caso aí, como é o nome dele?

VozFem: Joaquim é irmão dele.

A: O senhor lembra desse caso, do seu irmão?

OF6: Lembro.

A: Conta aí pra mim o que que ele estava fazendo, como é que foi.

OF6: Ele foi caçar irmão.

A: Hum.

OF6: Naquele tempo tinha caça aí perto, né. Como agora(...)

A: (...) Lá em São Joaquim.

OF6: Lá em São Joaquim e pra trás.

A: Hum.

OF6: Ele foi caçar, ele com o curumim::.. era meu irmão até mesmo. Aí ele foi caçar pra lá. Quando ele ia passando e o cachorro ia na frente ele caçava com o cachorro. Dois cachorro, ele ia passando irmão parece que o cachorro passou não deu pra ele pegar e ele vem atrás:: surucucu pegou ele na perna dele. Hum o homem caiu aí os gritos. Aí grita e olha ele aperreado ~~no pé viado~~. Nem matou a cobra nem nada, né? Aí daí ele vem embora, vem embora (...)

VozFem (...) surucucu é mais venenosa que jararaca.

OF6: (...) E aí quando ele chegou na beira da... da mata virgem que chama né?

A: Hã

OF6: Que é a do começo da da... da.. roça, essa capoeira. Aí ele mandou curumim (...)

A: Chamar o senhor.

OF6:Chamar nós. Curumim era pequeno ele correu, vinha aos grito gritando, arrastando. De quatro pé. Não podia levantar mais. Isso tava inchado até aqui ó. Papai é meu tio, dois tio meu mais os primo dele, os seis irmão correram (inint) [00:35:59]

A: E quando chegou lá o que que ele sentia?

OF6: ele via aos gritos que ave maria. Tu sabe que quando do homem grita não é...
qualquer dorzinha que ele está sentindo não né?

A: E aí e aí o que que o senhor fez de tratamento pra ele?

OF6: Eu não estava

A: ah, não tava (...)

OF6: (...) tava pescando. eu tinha saído pra pescar. Só o papai, com meu irmão, mas o tio meu e foram pra lá e buscar ele. Quando ele chegou, aí eu cheguei lá já era mais tarde.

VozFem:(...) foram buscar ele lá onde ele tava pescando.

OF6: foram buscar até lá. Trouxeram ele na rede até em casa na vida em São Joaquim onde eu ainda estou morando. E aí eu cheguei lá ele já estava ele aos grito. Tava tudo inchando e botando sangue. Sangue pela boca, pelo nariz, pelos olhos, pelo ouvido. Por tudo que ela:: teve::.. saia sangue, dos dente tudinho. Não parava de jogar sangue.
Aí eu:: sabe tem muitos logo ficam assustado né?
A: Aham.

OF6: Aí eu estou indo aí quando chego ele já tinha levado ele pro hospital.
Já tinha levado ele pro hospital. E aí cheguei lá em casa mamãe estava chorando aí e papai estava com ele no hospital. E:: e agora? E lá o doutor também não queria
que ninguém fosse ver ele né? ele passou 10 dia irmão, [00:37:36] naquele tempo ninguém podia entrar [00:37:48] E mesmo também já, ainda num tava com essa como disse essa força de reza. Eu ainda estava novo também. Aí quando ele saiu meu irmão aquilo ficou desse tamanho assim. Oxe.

A: A perna ficou bem inchada(...)

OF6: (...) perna ficou bem inchada. Ficou paralisada. Aí fizemos tanto do remédio, remédio, injeção. Naquele tempo era benzetacil e ainda tava não esse outro como é::. Como é que era o nome daquele remédio? Penicilina.

A: Uhum.

OF6: era injeção de penicilina. Meu cunhado dá um dia, dá outro dia não.

A: Isso ele já estava em casa?

OF6: Já... já estava em casa. Meu irmão ele passou quase um ano pra ele poder andar. eu com aquele maior aquele pequeno sabedoria que Deus já está apontando né?

A: Uhum.

OF6: Eu fui fazendo ~~feitiçando~~ fricção com óleo de todos os bicho.

A: Que que óleos que o senhor passava?

OF6: Eu passava óleo de... de... de guariba, macaco, guariba.

A:Uhum.
OF6: Óleo de jacaré. Óleo de.. de arraia.

A: Uhum.

OF6: Óleo de:: comé de onça. Esses quatro óleo ia passando.

A: Tudo junto?

OF6: Tudo junto.

A: Aí fazia massagem em cima?

OF6: Fazia massagem pra afrouxar o osso dele aqui. Porque estava tudo preso.

A: Uhum.

OF6: Não podia mais andar só com um lado aqui outro lado ele não mexia [00:39:21] mas graças a Deus com o poder de Deus ficou bom. Ficou meio aleijado, não ficou aleijado... manquejava um pouquinho [00:39:32] mas um lado da perna ele ficou meio fino.

A: Entendi. Afinou um lado da perna.

OF6: Afinou o lado da perna.

A: Ele não.. não é mais vivo?

OF6: Tá vivo demais.

A: Tá vivo. Onde ele está?

OF6: Hã?

VozFem: ele tá no castanho. Ele mora no castanho.

OF6: Ele mora no castanho.

A: Ah no careiro castanho.

VozFem: É. Lá que ele mora.

A: Ele é seu irmão.

OF6:É meu irmão.

A: E ele é mais novo ou mais velho do que?

OF6: Ele é mais novo do que eu. Ele está com mais uns trinta e tantos ano que ele foi embora... ~~no campo~~.

A: nunca mais voltou

OF6: Nunca mais falei. Tem falado assim com pelo telefone no
celular

A: e lá em São Joaquim o senhor conhece outras pessoas que foram picadas
por cobra ou mesmo em São Paulo de Olivença como um todo que foram picado
por cobra e que tem::.. ficaram com sequela?

OF6: Eu estou dizendo pro senhor que quando chegar picada de cobra lá do mato já leva pro hospital eu não posso nem dizer (...)

A: (...) não mas esses mais antigos mesmo.

OF6: ah. Antigo que eu conheço somente esse meu compadre Fifi ~~Xixi~~ que é o Manel, né?
A: Que era lá da comunidade?

OF6: ele mora na comunidade. Depois disso, pronto. É, ah cobra mordeu o fulano já está no hospital. Tem um comadre meu que mora bem assim pertinho. Como essa casa é mais ou menos, né?

A: Do lado da sua casa?

OF6:Do lado da minha casa.

A: Também foi picado?

OF6: Foi picado. Levaram daí mesmo pro... pro hospital. Nem do... do caminho da.. da roça. Passou duas semana no hospital e inchou.

A: Uhum.
OF6: inchou que não podia mais andar. Passou um mês, dois meses pra Manaus, tá vendo? Eu bem aí, mano. Né? Eu não vou dizer, vou percurar, eu vou atrás do que um dia sabe, né? Por exemplo: o senhor posso, eu sempre posso tá doente ali, mas essa hora não vai saber logo na hora, né? E quando se eu saber que já...já é tempo que leva pro pro hospital, né? E assim eu fico aí ~~o Chico~~ também eu não vou lá, né? era 06:07 olha vamos ali que é é meu sobrinho né? Vamo alí com titio quem sabe se ele não sabe algum remédio ele reza né? Então aí parecia era coisa fácil, mas não vamos eu também não vou lá atrás né? Que às vezes eu nem sei quando vou saber já tá no hospital, né? Eu não posso ir lá.

A: seu Anísio... o senhor existe algum tipo de, alguma utilidade quando a gente mata a cobra? É:: algum alguma coisa que se possa fazer com o tratamento com a própria cobra ou não? A gente tem que descartar ela.

OF6: Eu ainda não experimentei, irmão.

A: Uhum.

OF6: Esse aí eu não posso dar eu prova, né?

A: Uhum.
OF6: Que eu ainda não experimentei, mas eu ouvi falar.

A: O que que você ouviu falar?

OF6: E quando ela morde a gente não se alembra né?

A: Uhum.

OF6: Corta um pedacinho de rabo desse tamanho. Pedacinho assim oh, ponta do rabo. Pode engolir que diz que também não dói mais.

A: O rabinho da cobra.

OF6: O rabo da cobra, mas eu nunca fiz aquilo. Às vezes a gente com tanto espanto que a gente tem e não quer saber de rabo é.

A: Se lembra só de corrida da cobra.

OF6: É.

A: É. e o senhor a considera importante de quando a gente for pra roça, pro mato, pescar, utilizar a bota, perneira, calça comprida pra se proteger?

OF6: Pra mim eu fiquei velho com caça comprida que nem bermuda nunca vesti da minha vida. Minha filha (inint) [00:43:06] eu lá quero bermuda que eu qualquer busca de minha perna minha perna (...)

VozFem (...) e tem mais uma papai o senhor tá esquecendo.

OF6: É.

VozFem: Quando a gente era pequeno assim tudo:: moçota, rapaz que tem meu irmão.

A: Uhum.
VozFem: Tem aqueles pau que é comprido tipo (inint) [00:43:24] que é tipo uma cobra.

A: Sim.

VozFem: Aquele pau ele cortava bem rés a terra. Aí ele pegava começava a dar em na gente.

A: O senhor lembra dessa dessa planta, dessa árvore que parece a pele duma cobra?

OF6: Sim, eu lembro (inint) [00:43:44]

A: que árvore é essa? E o que que a gente deve fazer?

OF6: Aquele (inint) [00:43:48] que bate a árvore?

A: Aham.

OF6: E tira o sumo, mas tem a batata dela que é grande assim. Arvore dessa altura já tem batatazinha. Você tira a batata, lava bem e rela (rala).

A: Hum.

OF6: Rela [00:44:03] aí bota um pouquinho de água e toma também. É bom pra isso. E aquela arvore [00:44:08]? Bate também e amarra aí.

A: Na perna.

OF6: Na perna.

A: Que é pra proteger?

OF6: É.

A: Então além da calça, da bota, da perneira, também a gente pode proteger (...)

OF6: (...) proteger e defende a gente.

A: defende a gente.

OF6: Eu nunca usei bermuda.

VozFem: ensinava a gente quando a gente ia pra roça.

A: Hum.

VozFem: A gente achava que ele é bem fininho (...)

A: eu conheço, eu conheço.

Vozfem: a gente cortava o aqui nas pernas que pra:: espantar a cobra, pra cobra não chegar perto da gente.

OF6: isso.

A: Também é uma forma de proteger pra afugentar a cobra.

OF6: pra afugentar a cobra.

VozFem1: Graças a Deus nunca foi picada né (inint) [00:44:42]? Por cobra a gente andava assim não a gente encontrava.

A: Seu Anísio é a pessoa que é picado de cobra ela pode comer qualquer coisa ou tem alguma restrição alimentar?

OF6: porem como:: logo no dia né? Não tem problema né? Pode, não pode não pode comer só bicho, peixe de esporão, né? Surubim, pirabutão, pirarara, esse bicho de esporão.

A: Esses daí são remosos?
OF6: São remoso ~~remoto~~.

A: O que que ele pode comer?

OF6: Pode comer, tambaqui, traíra, que chama pongó também. Tanto do nome né? E... Curimatã. Tudo isso você pode comer::.. sardinha, pacu mas família de esporão

A: E bicho de casco, pode comer?

OF6: De caça só [00:45:39]... a carne de anta.

A: Não pode.

OF6: Não pode comer (carne de anta) porque senão ele vai sentir. Naquele Deus o livre. [00:45:48] Carne de anta ela não presta pra nenhum paciente que está sofrendo alguma coisa não come. Por quê?

A: É remoso até pra quem está bom.

OF6: É só quando fica bom. Agora do mato só é veado.

A: Hum. Esse pode?

OF6: veado serve até pra mulher de parto comer. Mas outra coisa de bicho nem dá irmão porque senão bicho vai dar hemorragia na mulher que capaz de matar, irmão. Isso aí não presta mesmo. Carne de porco do mato, carne de é o porco de casa mesmo. Olha:: minha mulher quase não ia morrendo.

A: Hum.

OF6: Você sabe o que é branquinha, né?

A: Sim.
OF6: E chama branquinha e chorão e você escuta aí ela eu tinha trazido no outro dia tinha uns quatro assado ~~(calçado)~~ [00:46:41] em cima do forno, ela passou sal pra comer. Estava com um mês ela tinha...operou... do filho..,não sei do qual é deles...nao me alembro... ela foi comer meu irmão deu hemorragia quase que

mata. branquinha aqui né eu... eu não dou conselho pra ninguém mas eu aviso logo quiser sofrer pode comer branquinha quando tiver gestante ou depois que tem criança.

A:É muito remoso.

OF6: muito remozo.

A: Pra qualquer situação?

OF6: é, qualquer bicho. É.

A: Entendi.

OF6: Ah mas se eu for lhe contar nós vamos anoitecer, vou amanhecer contando aqui.

A: não, até a gente já tá encerrando ó, muito obrigado pela sua:: disposição, por senhor compartilhar todo o seu conhecimento. E aí eu tenho só uma última pergunta pro senhor. É o senhor:: pretende passar esse seu conhecimento pra algum neto, pra algum filho, pra que outras gerações também disponham desse todo conhecimento que o senhor tem?

OF6: Meu irmão ainda não achei um que tenha coragem.

A: Por que que é difícil encontrar?

OF6: Porque eu::.. eu não conheço, mas eu sei né? Por exemplo olha senhor gente olha
Se eu disser assim, olha, seu Anísio eu quero trabalhar assim porque o seu trabalho
eu vou fazer isso assim porque eu sou homem.

A: Uhum.

OF6: Tu tem coragem? E aí eu tenho, porque já experimentei meus filho tudinho nenhum mano. Eu digo porque eu criei estava todo grande, todo pai de família, pai de neto. Quando eu dou uma força pra ele e vamos caindo. Hein? Cadê o valentia, deles? Nenhuma. (...)

A: (...) o senhor ainda não encontrou uma pessoa forte o suficiente pra:: pra aguentar. (...)

OF6: (...) forte pra aguentar. Já digo pra muito mano. Olha aqui eu já trabalhei muito e hoje vou trabalhar. Vem dez paciente hoje aqui. Mais de dez hoje porque já já estão já estão sabendo que eu estou aqui né?

A: Uhum.

OF6: Eu acho que estava aqui já estava tudo mais ou menos aqueles (inint) [00:49:01] Eu vou contar a minha passagem porque se não sabe você vai se admirar que eu tenho um doutor que trabalha comigo ele faz cirurgia você acredita isso? Senhor acredita? Ele faz cirurgia da hérnia, pedra na vesícula, o que for.

VozFem: Saúde tudo espiritual.

OF6: Saúde espiritual. Olha eu vou mostrar com que eu trabalho pro senhor mais que eu tenho. E antigamente a minha filha me da essa bolsa.

A: O tabaco é um agente importante no seu trabalho?

OF6: É importante.

A: Por quê?

OF6: Porque é aí que tá a força da...da... da tua oração. Pra muitos né? Pra muito não, não é pra todos, não é pra todos. Maioria é pouquinho, mas resto eu trabalho bebida de forte na só só refrigerante é outra coisa olha o teu trabalho e percora.

[00:50:10]

Entrevista Cuidador 7

[00:00:03]

A: A gente vai fazer uma entrevista contigo, baseado num...num tratamento, como é que você é::... sobre acidente ofício, tá? Picada de cobra, mas inicialmente eu queria que tu falasse um pouco como que você gosta de ser chamado, reconhecido na comunidade como pajé, como xamã, como curador, me conta um pouco.

OF7: Eu?

A: É.

OF7: É...primeiro que o pajé (faz) é chupar. (Se referindo a principal forma de atuação dele como pajé) Quando o pessoal daqui tudo... tudo dia que eu chamar chamou, chamou vou ir pra lá na casa dele hoje de tarde, de manhã e de noite (...)

A: (...) Qualquer::.. qualquer horario

OF7: Qualquer hora aqui pra chamar eu. (...)

OF7A: (...) Que eles chamam de Pajé, né?

A: Eles te chamam de Pajé.

OF7: Uhum.

A:Você gosta de ser chamado de pajé?

OF7:Gosto.

A:Gosta. É uma coisa natural.

OF7: Uhum.

A: E como:: com que tu começou a ser solicitado, reconhecido pela comunidade como pajé? Com quantos anos você tinha tu lembrado? Como você começou?
OF7:hum, cinco ano.

A: tinha cinco anos de idade?

OF7: Cinco ano.

A: cinco anos de idade.

OF7:aham.

A: É? E como que foi?

OF7:Aí que:: começou:: foi pra Manaus primeira vez.

A: É. Criancinha.

OF7: aham, dezoito ano.

A: Ah com dezoito anos.

OF7: Dezoito ano fui pra lá. Aí levou um patrão que Manaus

A: aham

OF7: aí foi lá esse meu patrão um batuco.

A: É uma o quê?

OF7: Batuco.

A: Que que é o Batuco?

OF7: Batuco? É. Batuque.

A:Tem batuque de pajé? De tocar?

OF7:É.

OF7A: Levou ele como se fosse tipo num::.. uma (...)

A: (...)Uma seara de umbanda?

OF7: É um local de um encontro.

A: Encontro de Umbanda?

OF7A: Encontro de Pajé.

A: De Pajé? Ah e aí?

OF7: Aí que o meu patrão aqui ó. pa ele que entrega de pé que o sabia, o que sabe que o apega é de mim.

A: ele que reconheceu?

OF7: uhum

A: Que tu tinha esse dom.

OF7: Uhum

A: É? em Manaus isso?

OF7: Manaus. Aí que meu patrão pra levar pra casa dele né. Espera aí que eu fazer o negócio, né?

A: Os trabalhos.

OF7: Trabalho. Aí que eu saí com cinco mês lá com ele.

A: aí tu aprendeu com ele?

OF7: aham. Aprendeu. Todo que aprendeu por cinco... cinco... cinco::.. (...)

OF7A: (...)meses.

OF7: Cinco mes. Aí que:: tudo sabe, né? O que que reza (...)

A: já sabe usar o dom e as rezas? E tu usa além da reza? Você usa alguma coisa mais alguma coisa? Tabaco (...)

OF7: (...) tabaco

A: Que mais? O que é importante?

OF7: O:: álcool

A: álcool.

OF7: vela

A: vela

OF7: esse vela de esse grande (...)

A: (...) dessas grossa.

OF7: Uhum. Esse é pequeno também aqui.

A: Dessa comum também.

OF7: Tudo sabe já, com cinco mês. Aí que problema de FUNAI que procurar pessoal lá aí pega FUNAI aí vai embora. Se Funai que mandar vai embora.

A: Uhum.

OF7: Vem pra cá.

A: Ah pra ti voltar pra Belém pra Belém do Solimões.

OF7: uhum

A: Mas e quem te mandou pra lá foi a FUNAI também?

OF7: FUNAI.

A: É? Porque era FUNAI que tá proibido lá.

OFA: Ele tá dizendo::.. peguei:: ele tá dizendo que esse patrão levou ele pra... pra Manaus tipo assim pra ele ensinar como que fazia a pajelança.

A: entendi. Era isso Silvio?

OF7A: Aí::.. (...)

A: a FUNAI.

OF7A: Aí ele ficou cinco meses fazendo o trabalho lá em Manaus e a FUNAI soube e aí a FUNAI que resgatou ele, mandou ele de volta (...)

A: (...) mandou ele de volta. Entendi.

OF7A: entendeu? Não foi que ele foi aprender. Ele é daquela situação, tipo assim, com cinco anos de idade, aqueles que nascem com cinco anos(...)

A: (...) ah com 5 anos já foi detectado o dom.

OF7A: Isso.

A: com dezoito o teu patrão te levou pra Manaus pra ensinar lá pras outras pessoas.

OF7A: Isso.

A: Só que a FUNAI pediu (...)

OF7A: (...) Cinco meses resgatou ele pra aldeia. (...)

A: (...) Resgatou você de volta pra aldeia e aqui você poderia trabalhar tranquilamente. É isso?

OF7: isso.

A: Entendi e...como você tem uma religião?

OF7: É católico.

A: Católico. Oh Sildo e você faz trabalhos caseiros, medicamentos caseiros pra tratar picada de cobra?

OF7: Exato.

A: É? Explica pra mim como que tu trata quando uma pica a cobra?

OF7: Aí que (...)

A: (...) e o quê que tu utiliza?

OF7: esse minha mãe mordeu cobra lá no fora.

A: Mordeu a cobra (...)

OF7: (...) mordeu

A: lá fora.

OF7: mordeu cobra lá fora. Aí boca de sacaia lá, conhece sacaia? (Se referencia à Comunidade Assacaia)

A: Assacaia. Conheço.

OF7: E:: faz tempo isso.

A: uhum.

OF7:que mordeu bem aqui.

A: Que cobra que era?

OF7:Jararaca.

A: Jararaca mesmo.

OF7:Jararaca. quando não tem motor::.. não tem... tem nada pra remar lá, pra caçar, pescar aí não tem condição, não tem motor pra trazer aqui.

A: Uhum.

OF7: Aí procura remédio num sabe que é uma árvore?

A: uhum.

OF7: Como é que chama? Culé.

A: Culé.

OF7: Culé.

A: Lá na na outra que tem o leite.

OF7:Ainda tem leite. Esse leite branco.

A: na árvore de Culé.

OF7: uhum.

A: tu já trabalhava?

OF7: (inint) [00:07:07]

A: Que é Culé.

OF7: Culé. Ai que tirar (inint) [00:07:12](...)

A: (...) tipo seringa.

OF7: uhum. Ai meter esse::.. qualquer (...)

A: (...) vasilha (...)

OF7A: (...) lata.

OF7: lata.

A: e aí faz o quê?

OF7:Trazer aí::.. uma pra levar casa::.. a casinha né?

OF7A: uhum.

OF7: fazer um::.. (...)

OF7A: (...) mistura

OF7: desse tamanho com leite.

A: Com leite.

OF7:Aí misturado com água pouquinho, pouquinho.

A: Uhum.

OF7:Aí coloca sal. Aí no mais::. (...)

A: (...) sentindo dor (...)

OF7: (...) sentindo dói. Sentir dói. Passar o::.. (...)

OF7A: (...) no local.

OF7: (esse pura... esse pura) [00:07:52] de branco.

A: mas toma ou passa sobre o local?

OF7: não, depois.

A:Ah (...)

OF7: ele passa, passa. Só::.. aí depois toma pouquinho.

A: um pouquinho.

OF7: misturado com sal depois misturado com água, misturado com...com raiz de açaí

A: com raiz de açaí

OF7: aí que bater né.

A: machuca bem.

OF7: machuca bem. Aí coloca aí

OF7A: fervido ou não.

OF7: panelinha que eu:: que raiz de açaí.

A: Uhum.

OF7:Já está pronto e coloca pouquinho de leite (...)

A: (...) leite do Culé.

OF7: já, toma. Aí que passa. Cinco minuto pa chegar aqui.

A: uhum.

OF7: aí sai sangue.

A: da... da picada.

OF7: uhum. Sai sangue, sai sangue, sai, sai. Já::.. melhora.

A: Passa a dor?

OF7:Passa a dor.

A:E... e... e... na tua vida tu já fez isso pra várias pessoas?
OF7: Aí que, aí que eu que eu chupar de negócio aí

A: ahn

OF7: esse bichinho.

A: entendi.

OF7: Aí que chupar. Por esse que tá pajé né...

A: aham

OF7: aí mesmo que ::.. vinte ano tá com (...)

A: (...) e como é que é o processo? Primeiro faz remédio, passa, toma um pouquinho aí cê passa pro processo que é pra chupar.

OF7: Uhum, aí o chupar.

A: aí o chupar que tu fala é com o cigarro.

OF7:É tabaco.

A: Com tabaco, com cigarro de tabaco.

OF7:Tabaco.

A: Com papilinho.

OF7: Papilinho.

A: Ou é só no tabaco direto? Fumando com papilinho.

OF7: Fumando.

A: É.

OF7: aí que tira chupar se cobra de veneno, né? Pra tirar.

A: hum. Entendi.

OF7: Passa.

A: Aí passa.

OF7: passa.

A: e...e precisa fazer quantas vezes esse processo? Quantos que é o suficiente ou só uma vez já é suficiente?

OF7: é uma vez.

A: Uma vez.

OF7: Uma vez.

A: Faz bem feito e aí já passa.

OF7: passa. porque já passa e não trouxe aqui.

A: aí não precisou nem pro... pro... pro hospital?

OF7: Não precisa.

A:É. E quantos casos mais ou menos assim durante a tua vida tu já tratou dessa forma?

OF7: é... dezessete ano.

A: Há dezessete anos que tu faz, então todo ano tu trata com esse:: dessa forma.

E tu já foi picado por cobra? nunca foi.

OF7A: a cobra já te mordeu?

A:Nunca te mordeu?

OF7: Só mordeu o:::

OF7A: escorpião.

OF7: O escorpião

A: o escorpião te mordeu?

OF7: Escorpião e aranha só isso daí.

A: e tu faz algum trabalho pra te proteger da cobra? O pajé tem algum poder, alguma reza, algum ritual de proteção quando ele vai pro mato pescar, caçar? O que faz?

OF7: Aí que eu lá no mato sozinho.

A:Uhum.

OF7: É rezar.

A: Faz oração.

OF7: Faz oração. E ninguém (inint) [00:11:34] e vem pra trás, pra trair (...)

A: uhum.

OF7A: (inint) [00:11:38]

A: ah nem o... nem o espírito não pode te atacar.

OF7: atacar outros e outro pajé né? Aí que eu é de madrugada. Aí quis:: aí fala comigo o outro pajé, meu (inint) [00:12:07]. Aí quem fala com outro pajé.

A: É um pajé, um espirito de outro pajé.

OF7: aham. Outro espírito de pajé tá falando.

A: uhum.

OF7: Como é que está falando agora.

OF7A: pra proteger.

OF7: pra proteger, pra proteger alguma coisa.

A: Entendi. E quando uma pessoa é picada de cobra ela precisa ficar de resguardo, tem alguma coisa remoso? Como é que é esse tratamento? O que que o pajé orienta uma pessoa picada de cobra? Que ela não deve fazer ou quê que ela deve fazer? Como... como que é?

OF7A: Tipo comida, pode comer tudo, que não pode comer?

OF7:é::... só pra:: não beber água não.

A: não pode beber água.

OF7:Não

A: não bebe. O que acontece se beber água?

OF7: Porque o se mordeu cobra

A: uhum.

OF7: aí que entra esse (filhinho) [00:13:10] cobra, né? Entra e entra aqui. Tudo o corpo vai entrar. Sim.

A:Vários filhinhos?

OF7:É. Vários filhinhos de cobra. Eu:: pequenininho, pequenininho isso daí que tá dói.

A: Ah é isso que causa dor.

OF7:Não beber água não. Quando o pajé vem aí pa...pa fazer o negócio aí pra ele não subir pra filhinho.

A: uhum.

OF7: Mas eu::: pa rezar:: oração. Aí aqui tá parando.

OF7A: Quanto tempo depois que pode beber água?

OF7:Na::.. em dois dia.

OF7A: dois dias.

A: Fica dois dias sem beber água?

OF7:Se bebendo água pra esquentar pouquinho (...)

A: Água quente.

OF7:Água quente.

A: Água quente.

OF7: Água quente. Não é muito só (...)

A: pra passar um pouquinho a sede.

OF7: E esse outro se::.. gestan::... (...)

A: (...) gestante

OF7: gestante de mulher.

A: Aham.

OF7: E não isso daí que é perigoso.

A: Perigoso pra mulher ou perigoso pra quem está picado?

OF7:É esse daí que tá mordeu o copo.

A: É o que que acontece? Conta pra mim

OF7: esse que tá esse mulher grávida.

A: Aham.

OF7: Aí que num pode olhar que morder cobra. É daí que tá perigoso (...)

OF7A: (...) Acontece se você chegar perto?

OF7: Esse::... esse negócio aí que tá morde. Aí que tá esse mulher grávida não aí:: (...)

OF7A: (...) entra nela.

OF7:Entra nela. E dói, dói dói dói dói.

A: A mulher fica com dor.

OF7: Aham.

OF7A: O bichinho que está ali pode sair pra ela.

OF7: Isso daí que tá esse copo aqui (...)

A: as cobrinha passam pra mulher.

OF7: uhum.

A: É? E aí o que que acontece com a mulher? Ela sente dor ?

OF7: Aí que pajé sabe, né? tirar esse, tirar tá com::... um dia.

A: Precisa de um dia pra tirar tudo? (...)

OF7: (...) um dia pra tirar tudo.

A: É. E com a e a pessoa que foi picada acontece alguma coisa quando a mulher grávida olha ele?

OF7: Nossa. (esse aí não) [00:15:34]

A: Não o... pro... o perigo é pra mulher?

OF7: Perigo, perigo, tem que sair.

A: Tem que sair, não pode ver.

OF7: não pode ver.

A: entendi.

OF7: só isso daí. Problema de cobra.

A: E alguma coisa em relação à alimentação? Tem alguma (...)

OF7: alimentação::... qualquer peixe pra comer (...)

A: (...) pode comer? (...)

OF7A: (...) Qual que não pode?

OF7: Só água.

OF7A: Não, qual peixe que não pode comer?

A: Tem algum peixe que é remoso?

OF7: Quanto que é qualquer peixe.

OF7A: Pois é, mas tem algum que ela não pode comer? Tipo, pode comer pacu?

OF7: Não, pacu não. não.

OF7A: Qual que pode (...)

A: (...) pois é fala pra mim quais são os peixes que não pode comer? (...)

OF7A: (...)São que pode comer.

A: O que pode comer?

OF7: Pode começar de sardinha. O:: traíra.

A: Traíra.

OF7: Esse daí é bom.

OF7A: Curimatã.

OF7: Curimatã.

A: Esses três pode comer?

OF7: três.

A: E se ele (...)

OF7: Pescada.

A: Pescada também pode?

OF7:Pescada pode comer. De cobra, né?

A: Aham.

OF7: surubim nada.

A: Por que não pode comer surubim?

OF7: porque tem::..

A:tem o quê?

OF7: Tem coisa e:::

OF7A: esporão

A: tem (cor) [00:16:52] ou tem esporão?

OF7: E daí que tá perigoso.

A: É e aí se ele insistir e comer o que que acontece com ele?

OF7: Insistiu:: nada. Sentir é bom.

OF7A: Se ele comer.

A: Não, e se ele comer, o que que acontece? Se ele se ele comer surubim, ah, o Manuel foi picado de cobra ele não seguiu muito a orientação do pajé e ele comeu surubim, o que acontece?

OF7: Porque tem negócio aí de surubim.

A: É?

OF7A: esporão.

A: Aí o que vai acontecer com o pé dele?

OF7: Esse pé dói.

A: Vai voltar um fazer dor?

OF7: Volta. O:: (...)

A: (...) é como se esporão é::.. vai e reagir na inflamação? (...)

OF7: (...) é. É sardinha é bom. Curimatá...Pescada.. aqui não tem problema pra comer aqui::

A: pode falar.

OF7:Sulamba também isso daí perigoso.

A: Perigoso?

OF7:Isso, sulamba é muito:: é cobra grande, filho de cobra grande.

A: A sulamba é filho de cobra grande e aí é perigoso comer.

OF7: é perigoso pra comer. Tambaqui também.

A: Também não pode?

OF7: não pode. Por que o tambaqui vermelho que é o carne.

A: uhum.

OF7: aí que (...)

A: (...) É remoso?

OF7: Remoso.

A: a sulamba ela é um peixe mas ele veio originalmente da cobra grande.

OF7: É.

OF7A: Da cobra.

OF7: Cobra.

A: E e mesmo as pessoas no::.. dia a dia é bom ou não é bom o começo do ano?

OF7:Não, não três dia.

A: fica três dias sem comer.

OF7: Fica três dias, já passou o negócio aí pra comer. Aqui já.

A: Durante a tua vida, quantos quantas pessoas picadas de cobra assim? É cê já viu algum deles morrer? Conta pra mim algum caso.

OF7: algum dia morreu?

OF7A:Algum morreu picada de cobra? Tu conhece alguém?

A: Me conta aí.

OF7: Já.

A: Qual foi o caso?

OF7: porque se pessoal daqui faz tempo, né?

A: Daqui de Belém.

OF7: Daqui de Belém. Falta pra chegar esse coordenador né.

A: Uhum.

OF7: Morar aqui.

A: ainda não tinha nem DSEI nada aqui?

OF7: Nada.

A: Não tinha polo base.

OF7: Não tem polo base.

A: hã.

OF7: Só que lá no João parece tem uma:: (...)

A: (...) uma casinha.

OF7:Casinha. (inint) [00:19:47] mordeu cobra do pessoal daqui (...)

A: (...) e onde essa pessoa tava, o que ela estava fazendo, onde ela tava quando a cobra picou?

OF7: Aqui mermo. Aí que morder onde que levar? Não tem, não tem doutor, não tem enfermeiro, não tem nada. E chora, ele chora. Que hora que eu morder, nessa hora que morder tarde já morreu.

A: Morreu logo em seguida?

OF7: Morreu. Por isso que ninguém sabe que remédio:: (...)

A: (...) poderia fazer.

OF7: podia fazer.

A: Entendi. O::..

A: Oh Sildo, e... e quando vocês vão trabalhar na roça, na pesca, vocês usam algum tipo de proteção? Proteção física?

OF7:Aí só cuidado.

A: só cuidado.

OF7: cuidado na roça, capinar

OF7A: calça alguma coisa?

OF7: Cadê que bota não tem?

A: Não tem bota.

OF7: não tem bota.

A: nem calça comprida.

OF7: não tem.

A: nem peneira.

OF7: perneira, nada.

A: E se você recebesse, se o DSEI::.. se a SESAI desse pra vocês bota, perneira e calça comprida. Vocês usariam quando fossem casar, caçar, pescar? Usaria comunidade toda?

OF7: É. Hoje, hoje tem.

A: É.

OF7: Hoje tem. que é antigamente a gente tem agora que tá (...)

OF7A: algumas tem né?

OF7: Alguns tem, alguns tem

A: e eles gostam de usar pra se proteger? Tem uma boa aceitação.

OF7: Porque bota (...)

OF7A: (...) eles é uma bota e cuidado, bota e cuidado.

A: E quando a gente encontra uma cobra, quando você encontra uma cobra, o que o pajé orienta que deve ser feito?

OF7: esse pajé é bom pra orientá né?

A: Aham. População

OF7: população. Onde que morde cobra aí que chama, ele. Aí pajé foi pra lá. Aí fazer o negócio pra chupar.

A: Entendi.

OF7:Tirar esse bichinho, filhinho dele de cobra, tirar::... aí se não passa, aí que pra trazer aqui.
A: no pólo.

OF7: no pólo.

A: Pra tomar soro.

OF7: Pra tomar soro.

A: Entendi.

OF7: e daí que proteção.

A: Então se passar com o pajé e vim tomar o soro é bom?

OF7: É.

A:É mais seguro.

OF7:Mais seguro. Tudo agora que é todo pessoal proteção de polo porque é no Pajé se não proteção e não tirar essa coisa. Aí que o::... aí que o moleque grave esse daí que é perigoso mesmo, perigoso porque se morreu. Se olhando se esse::.. morder cobra momento que morreu de dor, sai sangue, tudo sai sangue porque é culpa dele. Você grave. Não sei que eu hoje esse tá grávida. Passar esse negócio aí.

A: Quando::... quando um uma pessoa é picado pela cobra, como que o pajé já entende? Por que essa pessoa foi picada? Ele fez alguma coisa pra ser picado.

OF7: é

A: é pode ser?

OF7A: pode ser um feitiço de alguém?

A: Pode ser uma coisa que foi feita pra cobra picar?

OF7: porque se não::.. se cobra morder e não e não é pajé pra mandar só pra morder cobra né? Aí que tá melhorando. Ficando bom. Senão outro pajé pa... pra coisa aí que fez o pajé fazer cobra, pra fazer (...)

A: (...) devolver?

OF7: devolver, buscar outra pessoa

A: que mandou fazer

OF7: mandou fazer um pajé. Aí tu cobra entrou no mato aí mordeu. Esse daí o outro pajé esse daí que::.. morreu. (inint) [00:25:27]

A: entendi. Alguém que mandou (...)

OF7: (...) aham

A: enviar. E o pajé? Tem um poder, um dom de interromper isso, de controlar a cobra pra não morder aquele que foi mandado picar?

OF7: É esse daí que eu sim::.. ele que sabe que chamar pajé outro.

A: uhum.

OF7: aí buscar. Se eu pegar aí que outro pajé que mandar se toca. Aí que tá tirando.

A: Uhum.

OF7:Negócio aí né? tirar, tirar, tirar e mais um, dois hora aqui cara

A: ele precisa de tempo pra quebrar o...o trabalho do outro pajé.

OF7:aham.

A: Entendi. E teria mais algum outro tratamento além do leite de culé que você que vocês fariam também?

OF7: já

A: é qual?

OF7: Esse:: Tu não sabe que chapéu de::.. chapéu de homem aranha que se chama

A: hum. O quê que é? uma planta?

OF7: É planta

A:do mato

OF7: do mato. É ali que tem.

A: Tipo mata-mata?

OF7: não.

A: Não? Esse tipo de castanha? (...)

OF7: (...) grande esse. Como é que grande folha.

A: Ah.

OF7: O tamanho desse altura.

A: kapeba? Não é kapeba.

OF7: esse chapéu de::

A: chapéu de?

OF7: de::.. chapéu de cobra.

A: chapéu de cobra é o nome de uma planta E aí o que a gente faz?

OF7: Bom::.. que não tem remédio quase que morreu lá no mato né?

A: Uhum

OF7: morder aí que eu sabia que tirar esse daí, duas folha pa tira, aí tem raiz (...)

A: (...) arranca tudo.

OF7: arranca tudo, aí corta, aí só raiz que tirar aí fervendo. Nessa hora aqui quando já estou pronto aí que tá bem vermelho esse... esse água.

A: uhum

OF7: Aí que toma só um copo né. aí toma. Cinco minutos já tá melhorando já. Aí que é bom o remédio pro cobra.

A: aí vai passando. E aqui quando o pajé faz o trabalho pra... pra.. pra picada de cobra, pra controlar o veneno, depois ele vai pra.. pra pro polo base, vai tomar soro em Tabatinga depois que volta pra aldeia, pra casa dele já tem algum::..algum tratamento que tu recomendas também?

OF7: É.

A: É. O qual?

OF7: Folha, folha de::: como é::... folha de ingá.

A: folha de ingá.

OF7: ingá.

A: Qual ingá? aquelas grandona ou das pequenas?

OF7: Da ingá grande.

A: Da ingá grande. faz o quê?

OF7: Bem... bem no ponto assim (...)

A: (...) O novinho.

OF7: novinho. Aí que tira. Tudo se tira, mas o tamanho desse. Aí que fervendo tá vendo aqui é remédio.

A: e aí o que faz? Toma?

OF7: toma

A: bebe o chá

OF7:toma, toma o chá

A: e o quê que esse chá faz?

OF7:É e daí que tá::: (melhorando)[00:29:44] se:: (...)

A: (...) tiver inchado.

OF7: Dói muito (inint) [00:29:52].

A: E algum filho teu foi picado de cobra? Só tua mulher?

OF7: Não.

A: Não.

OF7: Não.

A: A tua mãe na verdade foi picada (...)

OF7: (...) só a mãe.

A: Você fez alguma proteção pra sua família, pros teus filhos, pra ele se protegerem? Pra eles não serem picados é porque não aconteceu mesmo.

OF7: Quase que meu filho com caça lá no mato.

A: uhum.

OF7: Aí meu filho. Um dia de madrugada pra focar. No caminho ai procura ai paca.*(Cuniculus paca)* Aí proteger e bota.

A: Entendi. Tá.

OF7: Porque não tem bota já. Cobra desse tamanho. Jararaca mesmo.

A: Grande.

OF7: grande. Vê essa lanterna tá aqui no coisa. Cobra tá bem aqui no ponta do (...)

OF7A: (...) pé dele.

OF7: pé dele. Quase que fica aqui:::. a cobra tá aqui, a cobra ele levanta o::.. cabeça dele. Morder bota(...)

A: (...) aí volta.

OF7: De novo...

A: ah entendi. Segundo ataque.

OF7: segundo. Aí proteger enquanto vai embora.

A: A bota protegido bem.

OF7: É, protege bem porque não entra do dente dele.

A: Entendi.

OF7: De repente encher isso tira esse bota aí e joga.

A: Não presta mais.

OF7:Não presta.

A: Porque tem veneno.

OF7: Tem veneno em bota. Aí que olhando nada não tem.

A: Sildo você tem muito conhecimento de medicina indígena, de preparo e tudo pra usar. Existe alguém, alguém na tua família que vai receber esse conhecimento pra outra geração, pra continuar daqui um tempo, daqui::.. tem alguém na tua família que tu prepara pra isso ou não?

OF7: Não sei (...)

A: ou ninguém não tem interesse?

OF7: ninguém

A: ninguém quer.

OF7: Ninguém.

A: Ninguém quer ser pajé na tua família.

OF7: É, ninguém.

A: Por quê?

OF7: Porque o ninguém gosta.

A: É, mas as pessoas na comunidade num gosta de pajé?

OF7: Não.

A: Não?

OF7: No meu família

A: uhum

OF7: não gosta. Sou eu que gosto.

A: É?

OF7: Porque só eu.

A: Tu usa o teu saber de pajé pra ajudar e curar as pessoas.

OF7: Uhum.

A: E aí na comunidade o que as pessoas pensam do pajé?

OF7: Tem pessoal de fora né, tem muito pajé e:: (...)

A: (...) aqui em Belém? fora de Belém? tem muito pajé entre os Ticunas.

OF7: É, tem muito. Rapaz, velho, ixe tem muito.

A:É, tem pajé novo, pajé velho, toda idade.

OF7: esse daí que num::... num trabalho não. Que::: pa matar outro.

A: Pra fazer feitiço.

OF7: É pra fazer feitiço.

A: E deixa eu te fazer a última pergunta, o tu falou que entre os ticunas existem vários outros pajés por aí. É::.. os pajés entre eles... eles conversam, trocam, trocam de informação, de conhecimento. ou um pajé não conversa com o outro, conversa?

OF7: Conversa.

A:Conversa?

OF7: Conversa. Pra trocar, pra é só de madrugada, né? E noite.

A: Ah, não é fisicamente, presencialmente não.

OF7: Não.

A: É só por espírito.

OF7: É, é só espírito::.. falar outro pajé falar. Esse que saiu o espírito.

A: Uhum.

OF7: pra conversar em outro pajé.

A: com outro pajé.

OF7: em si mesmo que::::... o de cobra.

A: Entendi. Então é isso. Muito obrigado. Acho que você trouxe informações assim importantes bem pra gente que é da área da saúde. O que a gente que da área da saúde com o Manuel, equipe do polo todo que tá aqui, a gente:: (...)

OF7A: (...) (fala do soro) [00:35:12]. Importância aqui, então pra ele falar, ah quando perguntei dele se é bom ter o soro aqui.

A: Ah tá. E você acha importante ter o soro aqui no polo base do que ir pra Tabatinga?

OF7: Esse soro?

A: É o soro de de veneno de cobra pra curar não tem aqui no Polo Base, nunca teve, só tem lá em Tabatinga. A gente gostaria agora de trazer aqui pro Polo Base pra ficar mais perto (...)

OF7A: (...) tu acha importante.

A: Tu acha importante ter aqui no polo. Acha?

OF7: acho.

A: Por quê?

OF7: Porque o esse soro não tem e o pa leva lá, né?

A: aham.

OF7: Aí que eu::.. tão bom, pra levar lá no:::.. se tem soro.

A: Aqui (...)

OF7A: (...) Tá aqui (...)

A: (...) não precisa levar lá.

OF7: não precisa levar.

A: Entendi. Então é isso Sildo ó::... muito obrigado aí pela tua entrevista é::.. como eu falei anteriormente é... a gente da equipe de saúde como a equipe do Manuel a gente não é concorrente, a gente não é contra que o pajé faça os trabalhos nos picados de cobra, o que a gente quer que ele faça, mas também traga imediatamente pra receber o soro anti-veneno. Então uma medicina do pajé com a nossa medicina do branco ajuda as pessoas a não morrer, não ficar amputado, não ter que cortar do perna, E aí vai reduzir mais o impacto entre os Ticunas e qualquer população indígena. Tá bom? Muito obrigado. Dá uma água pra ele. Foi ótimo.
[00:37:13]

Entrevista Cuidador 8

[00:00:01]

A: Como:: como que... como que é as pessoas te reconhece? Como rezador, como o quê? O quê que cê faz na comunidade?

OF8: (inint) [00:00:16] modida cobra né

OF8A: não. É... tipo assim a Pedrina é rezadora, é pajé, é curadora, como é que eles falam (inint) [00:00:22]

OF8: Ah (...)

A: Como que tu é conhecida? Se eu chegar aqui procurando a Pedrina vão dizer oh é a:: como o quê que ela faz na comunidade? (...)

OF8A: (...) rezadora, curadora, pajé como é que é?

OF8: (inint) [00:00:34] (...)

A: (...) te conhecem (...)

OF8A: (...) como te chamam (...)

OF8: (...) cadê dona Pedrina sabe rezador (...)

OF8A: (...) rezadora (...)

A: (...) rezadora (...)

OF8: (...) rezadora (...)

OF8A (...) é. E que mais ? (...)

OF8: (...) e curadora.

OF8A (...) curadora. É.

OF8B (inint) [00:00:50] rezar (inint) [00:00:48] criança assim e fica (melhor) [00:00:52] criança pra rezar (...)

A: (...) então ela reza, ela cura

OF8: uhum.

OF8B: aí outra pessoa que pergunta onde fica (inint) [00:00:58] aí conhecida demais (...)

A: todo mundo conhece Pedrina na comunidade como rezadora (...)

OF8B: (...) aí pessoa que tem doença já procura ela (...)

A: (...) direto (...)

OF8B: (...) assim que (inint) [00:01:13] (...)

A: (...) entendi. Falam por aí que tu reza, que tu (...)

OF8: Pessoa no baixo, no Vendaval, Campo Alegre, tudo me conhece, no Feijoal, Umariaçu, no Tabatinga também .

A: toda região conhece a Pedrina como rezadora e curadora (...)

OF8: (...) agora tem muito né rezador (inint) [00:01:35] outro rezador não resolve (...)

A: (...) a Pedrina é a que mais resolve?

OF8: uhum

A: é a mais famosa da região? Que bom. E Pedrina quando que tu começou a rezar pela pessoa? Quanto tempo tu tinha de::...

OF8: tá com:: deze::: deze:: dezesseis ano.

A: quando tu tinha 16 anos de idade?

OF8: uhum.

A: onde tu morava.

OF8: lá no Urique I

A: Urique.

OF8: Urique.

A: uma outra comunidade aqui perto? E aí como que foi? Quem que te ensinou, como que aconteceu?

OF8: meu pai::.. meu pai, minha avó (inint) [00:02:22]

OF8B: ensinar a rezar (o pai dela) [00:02:28] (...)

OF8A: (...) pagavam ela. Pois é mas como que ela aprendeu? Quem foi que ensinou ela?

A: (inint) [00:02:31]

OF8: fazer o coisa.

OF8A: quem que te ensinou?

OF8: meu vô.

OF8A: ah, o avô dela (...)

A: (...) o avô (...)

OF8A: (...) teu avô (...)

OF8: (...) uhum (...)

OF8A: (...) que te ensinava?

OF8: uhum.

OF8A: na época da seringa (...)

A: (...) era peruano (...)

OF8: (...) uhum.

A: tua mãe... teu avô

OF8: uhum, meu pai.

A: teu pai.

OF8: minha mãe é ticuna mesmo.

OF8A: ticuna. É aqueles casos né.

A: e aí tu.. tu fo::.. aprendeu a rezar e curar (...)

OF8: (...) rezar, curar. Dois ano ensinava...

A: tu passou dois anos estudando com ele, acompanhando ele nas rezas (...)

OF8: (...) uhum.

A: e aí começou a fazer::

OF8: começou a fazer minha... tu conhece a Nathalia?

OF8A: uhum

OF8: ela não sabe não.

OF8A: só ensinou tu

OF8: uhum.

OF8A: era 4 irmão, só ela que aprendeu.

A: só tu que aprendeu com teu avó.

OF8: uhum

A: e desde lá tu começou.

OF8: aham.

A: criança, adulto, tudo.

OF8: tudo.

A: gente grande, velho, tudo. É.

OF8: e minha irmã que é Nathalia.

OF8A: hum

OF8: (inint) [00:03:58] já morreu (inint) [00:04:02]

A: tem outro irmão que também sabe?

OF8: uhum.

A: mas tá vivo ele?

OF8: tá

A: tá vivo. No urique

OF8: no Urique

A: hum::.. E Pedrina:: quando uma pessoa é picada de cobra o que que a gente deve fazer? O que tu faz pra curar aquela pessoa?

OF8: É chá.

A: chá de quê?

OF8:de copaíba.

A: copaíba. como tu faz? Descreve pra mim como tu faz.

OF8: Ferve...ferve um pouquinho...sete gotas pra ele... mangaratanaia também só um pouquinho (...)

A: (...) mangarataia (...)

OF8: ferve 7 gota de coisa (...)

OF8A (...) copaíba.

OF8: copaíba. Aí toma.

A: aí toma.

OF8: aí toma. Aí passa também só (...)

A: (...) passa copaíba em cima.

OF8: aí reza e pronto.

A: aí tu faz uma oração?

OF8: uhum.

A: como que vocês são? Católicos, da cruzada?

OF8:   cruzada.

A: cruzada.

OF8:   uhum.

A: e::: é:: tu já fez tratamento pra picada de cobra em muita gente aqui?

OF8: uhum.

A: é. Consegue:: onde que aconteceu? Onde que essas pessoas estavam quando foram picadas? Conta aí pra mim.

OF8: é(...)

OF8A: (...) a cobra morde eles aonde? Trabalho, pescando? (...)

OF8: (...) trabalho, capinando.

A: capinando geralmente, em que parte do corpo deles?

OF8: no aqui (...)

A: na mão e no pé (...)

OF8: (...) pé.

A: E aí eles te procuram para fazer::: (...)

OF8: (...) é.

A: Que::...que outros medicamentos:: que outros tratamentos também:: tu faz pra picada de cobra além desses que você descreveu?

OF8: tabaco também.

A: usa tabaco.

OF8: tabaco. Usa tabaco e sopra e aí não dói mais não

A: tem que soprar?

OF8: uhum. Quando ele dói muito né.

A: uhum.

OF8: sopra o tabaco aí para, também tem que tomar o remédio também.

A: tem que tomar o soro também.

OF8: soro também.

A: é. Normalmente tu atende lá.

OF8: uhum

A: e encaminha:: é importante tomar o soro daqui do hospital?

OF8: é.

A: é. E aí ajuda com a reza e mais o tabaco. É isso?

OF8: uhum.

A: O que que::: é:: quando a pessoa é picada por cobra o que que ela deve evitar? O que que ela não pode fazer pra não ficar ruim?

OF8: (inint) [00:06:28] peixe também não come.

A: não come peixe? Qualquer tipo de peixe?

OF8: só curimatã come.

A: qual:: qual peixe que ela pode comer?

OF8: curimatã, sardinha só.

OF8A: traíra não?

OF8: não.

A: só sardinha e curimatã.

OF8: (inint) [00:06:43]

A: e se ele insistir come outra comida?

OF8: fazem mal.

A: é? O que que acontece com o (inint) [00:06:52]

OF8A: (...) se comer surubim o que que acontece?

OF8: faz é mal.

A: é.

OF8: sai ferida.

A: sai ferida.

OF8: uhum.

A: agrava? (...)

OF8: (...) (inint) [00:07:00]

A: fica inchado.

OF8:: uhum.

A: Vocês trabalham na roça, na pesca?

OF8:: uhum.

A: é::vo:: (...)

OF8A: (inint) [00:07:12] roça

A: roça. E... vocês o que que vocês fazem pra se proteger? Pra não ser picado pela cobra já que vocês vão pro lago, mato e tudo.

OF8A: o que que ela usa? Calçado, bota?

A: não. Só isso mesmo (...)

OF8A (...) sapato

OF8: fazer remédio também no mato (...)

A: (...)faz o que? (...)

OF8A: (...) pra cobra não morder.

A: (...) conta pra mim (...)

OF8: (...) (inint) [00:07:39] menino pequeno né.

A: ahn.

OF8: batia no pé

A: com o que?

OF8: com remédio do mato num tem

A: com uma planta?

OF8:: uhum. (...)

OF8A: (...) tu sabe o nome da planta?

OF8: não. (nós não conhecemos) [00:07:54]

A: conta pra mim tem uma criança que ainda é nova, que é jovem, pequenininho ainda e tu quer prevenir pra vida toda. Aí faz o quê? Vai no mato (...)

OF8: (...) mato. Passa (...)

A: (...) pega a planta.

OF8: (inint) [00:08:10]

OF8A: tu reza pra num não acontecer nenhum espírito com ele? (...)

OF8: (...) uhum. É.

A: quando vai batendo na perna. Na perna que bate?

OF8: é.

A: e vai rezando (...)

OF8: não come cobra, não come cobra, não come cobra (...)

A: (...) ah essa que é o comando?

OF8: uhum

OF8B: tem uma... tem uma... mato né... assim lá no mato um... crescer e crescer como arvore dele parece jararaca né. esse daí é bom quando pessoa que picada de cobra né (...)

A: (...) hã

OF8B: isso tem que tirar isso daí no mato...levar né...aí passa como remédio pra rezador fazer pra depois pica cobra, a pessoa fazer isso daí (inint) 00:09:01]

A: esse mato aqui?

OF8B isso, isso daí.

A: é esse aqui?

OF8: uhum. Esse daí

A: é, então vocês vão mato pegam (...)

OF8A: (...) isso, isso daí.

OF8: (...) bate no (inint) [00:09:21] pega ele

A: pega ele e bate.

OF8: criança e nós também

A: da criança?

OF8: uhum.

A: e aí a cobra não pica mais.

OF8: não

OF8A: (...) a proteção dela quando ela vai pra roça é essa. Ela faz o serviço antes de ir pra roça.

A: toda vez que for pra roça tem que fazer?

OF8: não.

A: quando criança era só uma vez? (...)

OF8: (...) quando criança só uma vez.

A: entendi. E aí cê faz uma oração?

OF8: faz uma oração.

A: quando cê tá batendo o quê que você fala?

OF8A: morde cobra

A: morde cobra o que?

OF8A: come cobra

OF8B: não morde cobra, assim que bater né, esse... esse... (...)

A: (...) uhum. Vai batendo e cê vai falando?

OF8B: uhum

OF8: não morde cobra, não morde cobra, não morde cobra (...)

A: (...) no corpo todinho?

OF8: no corpo todinho

OF8B: (inint) [00:10:15]

OF8A: (...) chega perto

OF8B: chega perto nele não, quando olha pra ver jararaca ele corre

A: Se uma pessoa que foi feito (...)

OF8B (...) (inint) [00:10:35]

A: quando:: quando a pedrinha faz esse (...)

OF8A: (...) ritual

A: esse ritual em mim e eu vou pra um lugar que tenha cobra, a cobra não vem pra perto, ela vai se afugentar?

OF8: uhum.

A: vai correr pra longe?

OF8B: isso.

A: aí eu vou tá protegido pela (inint) [00:10:49] (...)

OF8B: (...) (inint) [00:10:51]

A: e aí tu faz nos teus filhos, teu marido, pra todos?

OF8: todos.

A: é. Mas aí é tu que tem que fazer:: o comando ou qualquer pessoa?

OF8: qualquer pessoa.

A: qualquer pessoa que tiver a planta pode fazer?

OF8B: isso

A: e por que que essa planta tem esse poder? Como que explica. De ajudar a gente de não ser picado::: não ser mordido?

OF8: (inint) [00:11:18]

A: como?

OF8: minha vó tava ensinando.

OF8A: mas o quê que ela é essa planta? O quê que ela tem? A planta o quê que ela é?

OF8: tem coisa também

A: é uma planta santa?

OF8: uhum.

A: é. Então por isso que ela é diferente as outras ?

OF8: é.

A: outras plantas não serve, tem que ser essa porque ela tem um poder religioso?

OF8: religioso.

A: é. Na... a senhora já viu alguém que foi picado por cobra morrer?

OF8: já.

A: é. Me conta como aconteceu.

OF8: ele dói pra ele...

A: foi homem, mulher, criança?

OF8: criança

A: criança?

OF8: uhum.

A: onde que a cobra picou ele?

OF8: ele, no chão (...)

A: (...) brincando (...)

OF8: (...) brincando.

A: perto de casa assim?

OF8: uhum.

A: aí mordeu onde mesmo?

OF8: aqui mesmo.

A: é. E aí o quê que aconteceu? O quê que já ele sentiu?

OF8: ele dói pra ele, chora, chora. Ele 6 hora mordeu e saiu (inint) [00:12:29]

A: (...) seis horas (...)

OF8: ele sabe se fazer remédio:: minha sobrinha.

A: teu sobrinho.

OF8: (a mulher morreu) [00:12:35]

A: quantos anos ele tinha?

OF8: 7

A: 7 anos.

OF8: uhum.

A: ele foi picado ao redor de casa.

OF8: uhum.

A: e não foi feito nenhum remédio?

OF8: não

A: ele:: não trouxeram pro hospital?

OF8: não

A: não? Se vcs recebessem é:: perneira, calça comprida grossa, bota, vocês usariam quando vocês fossem trabalhar na roça?

OF8: uhum.

A: usariam? Gostariam de usar? Não teriam problema não.

OF8: não

A: e é mais protegido

OF8: é

A: Quando as pessoas morrem, é:: quando a gen... quando a gente aqui na comunidade, vê uma cobra, uma jararaca que é o que mais tem na região, o quê que a gente deve fazer com ela?

OF8: É mata.

A: Mata?

OF8: uhum.

A: É?... E como que:: é:: o rezador vê a jararaca? O que ele... o quê que ela representa?

OF8A: Uma coisa ruim, espírito e tal (...)

A: (...) O quê que é? Como... como que...como uma coisa boa, ruim (...)

OF8:: (...) hum.

A: Traiçoeiro... é?

OF8:: uhum (...)

A: É:: e a... e uma pessoa tem poder de:: controlar uma cobra tipo mandar a cobra fazer alguma coisa assim? Morder um... (...)

OF8: (...) Sim (...)

A: (...) é? É possível?

OF8: É pajé fazer mal pra gente aí num tem coisa...de repente morre.., 5 minutos já morreu.

A: Um pajé pode (...)

OF8: (...) (inint) [00:14:17] (...)

A: (...) determinar que uma cobra morde ele, se eu tô com raiva dele?

OF8: uhum... daí de repente morrer (inint) [00:14:23]... fazer mal pajé pra:: gente, né?

A: uhum

OF8: Fazer coisa pra gente, pa é:: cobra (init) [00:14:32] pra gente morrer...

A: É possível?

OF8: uhum

A: Mesmo eu não tando perto, tipo ... uma pessoa que tá lá na outra comunidade (...)

OF8: (...) É (...)

A: (...) Eu posso ir lá com o pajé e pedir pra ele fazer alguma coisa assim?

OF8:: uhum

A: É possível?

OF8A: Por que que não pode chegar gestante, Pedrina? Perto da cobra de quem morde?

Voz Masc: A... (init) [00:14:59]

OF8: (inint) [00:15:00]

OF8A: é, o quê que acontece? (...)

A: (...) o quê que acontece?

OF8: Porque tá glávida (...)

OF8A: (...) Hum... e aí? (...)

OF8:: (...) De repente morreu.

OF8A: A bebê?

OF8: Bê... não a:: (...)

OF8A: (...) A mãe? (...)

OF8: (...) A mãe.

OF8A: É?

OF8: Quase morre...

OF8A: Hum

OF8: Tu:: tu:: mulher... tu tá:: tu mulher tá glávida, né?

OF8A: Hum

OF8: (inint) [00:15:15] (...)

OF8A: (...) Picada (...)

OF8: (...) (inint) [00:15:17] morrer:: (...)

OF8A: (...) Mordida? (...)

OF8: (...) Mordia o cobra...

OF8A: uhum.

OF8: Tu não pode olhar (inint) [00:15:22]... fazer mal.

OF8A: Hum::

OF8: mas (...)

OF8B: (...) E:: e:: pessoa grávida, né? Que vive perto de:: (...)

OF8A: (...) O marido também não pode olhar, não?

OF8: Não, não, não pode.

OF8A: Hum:: Num é só a mulher não, né? (...)

OF8B: (...) Tem que guardar, né? (...)

A: (...) Tem que se isolar (...)

OF8B: (...) Não chega nada, ninguém só o que (init) [00:15:36] picada de cobra que
tava (...)

OF8: (...) Quando tu olha... rum (...)

OF8B: (...) Guardar, né? (...)

OF8A: (...) Dor (...)

A: E o que acontece se olhar, Pedrina?

OF8: Morreu.

A: Morre?

OF8: (inint) [00:15:45] Morreu (...)

A: (...) Ah, Aconteceu já isso? (...)

OF8: (...) Saiu sangue no boca, quando tem aqui no ferida, aí sai... fazer mal quando é
pessoa grávida olhar.

OF8B: (inint) [00:15:56]

OF8: uhum.

OF8A: Mas o quê que é? Espírito que tem?

OF8: É espi... Não

A: O quê que é?

OF8: fazer mal mesmo.

OF8A: É?

OF8: uhum.

OF8: (inint) [00:16:06]...

OF8A: Hum::

OF8: Tudo pessoa.

OF8A: Tendi.

A: Algum parente teu j... já foi picado? Quantos filhos tu tem? 6?

OF8: (...) Seis (...)

OF8A: (...) Sete (...)

A: (...) Sete?

OF8B: (...) Sete (...)

OF8: (inint) [00:16:23]... morreu três, quatro (...)

OF8B: (inint) [00:16:27]

A: Esse ritual de proteção cê faz pra todos teus filhos?

OF8: uhum

A: É? Pra eles nunca serem picados?

OF8: uhum

A: Mais o teu marido?

OF8: (Não) [00:16:37].

A: E:: e:: eles nunca foram picados?::: Quando:: um:: um:: um picado de cobra, no caso, tipo:: várias situações pode agravar. Quando come comida que não é

pra comer... quando v... vê, ouve mulher grávida, é... ta... também tem problema. Quando... quando agrava, fica bem grave, pra morrer, quê que tem que ser feito?

OF8: gra... mulher grávida?

A: É, Ô:: a pessoa que tá picada de cobra.

OF8B: (inint) [00:17:18]

OF8: É... remédio, também é comida.

A: A comida?

OF8: uhum (...)

A: (...)Tem que ser... tem que seguir o resguardo.

OF8: É.

A: Tem alguma coisa que eu tô esquecendo? Tu já sabe mais do que eu, e eu que tenho que perguntar...Então é isso. Então esses são os remédios (...)

OF8A: (...) Ah, se ele... como que:: e tu já perguntou como é que ele entende, né? Por que foi picado... se é algum espírito e tal.

A: Existe algum remédio que eu posso fazer com mesmo, com o mesmo:: com a cobra mesmo? Com o pedaço da cobra? Com alguma coisa, não?

OF8: (inint) [00:18:09] pedaço de cobra, é comer...

A: Não?... Nem com a casca da cobra? Nem com a... não? Com a pele (inint) [00:18:16] (...)

OF8A: (...) Coração?

OF8: É

A: Ah, é? Faz?

OF8: Faz.

A: O quê que faz? Conta pra mim?

OF8: Não dói pra ela... não dói pra gente...

A: Pra quem foi picado?

OF8: Só pi... pequeno comer coração (...)

OF8A: (...) coração

A: come coração?

OF8: o rabo também pequeno

A: come um pedaço do coração (...)

OF8: (...) uhum (...)

A: (...) e um pedaço do rabo da cobra. Aí vai (...)

OF8: assim... assim como essa

OF8A: (inint) [00:18:41] cobra não.

A: isso, isso aí é um (...)

OF8A: (...) couro (...)

A: (...) é um couro de cobra? Que tu fez uma pulseira? É? E como que cê fez? Quem que fez? Quem matou essa cobra?

OF8: nós.

A: na tua casa mataram a cobra? E aí fizeram uma pulseira? E::.. o que que a pulseira tem?

OF8: tem oração.

A: tem uma oração (...)

OF8A: (...) protege (...)

A: (...) na pulseira.

OF8: (inint) [00:19:15]

OF8A: hum. Isso é como se fosse um escudo pra ela (...)

A: (...) um... um amuleto?

OF8A: é

A: é um amuleto de proteção que você utiliza, que a pedrina fica blindada? Ninguém consegue fazer mal. (...)

OF8A: (...) ela conhece quando a gente é ruim, quando chega perto dela (...)

A: (...) ah, ela conhece. Tu já sente.

OF8: tem muita:: pessoa né

OF8A: uhum.

A: tem muita gente.

OF8: uhum.

A: e aí o quê que cê faz?

OF8: (inint) [00:19:45]

OF8A: os cara vem querendo matar ela e (...)

A: ela conversa, faz oração e a pessoa (...)

OF8: (...) muda de coração

A: como... muda de:::

OF8A: (...) ideia (...)

A: (...) de comportamento, de ideia. É? Cê cobra pra fazer esses trabalhos, não?

OF8: não. Cobra também.

OF8A: ela cobra
A: ela cobra. E tem:: depende da pessoa ou tem:: cê não cobra?

OF8: também.

A: todo mundo?

OF8: todo mundo.

A: é.

OF8: depende de qualquer sabe (...)

OF8A: qual...qual serviço né?

OF8: uhum.

OF8A: ela depende (...)

OF8: (...) que não sabe.

OF8A: se ela descobre aí paga né (inint) [00:20:44] se ela não descobre:: é assim aí não paga não. É?

A: aí cê tá::.. deixa eu te perguntar uma coisa existem pessoas que procuram você pra várias situações diferentes

OF8: uhum.

A: por picada de cobra ou porque perdeu alguma coisa. Então as orações variam, tem uma oração só pra aquilo ou é a mesma oração que cê faz?

OF8: tem

OF8A: é diferente
OF8: diferente

A: pra cada situação são orações diferentes?

OF8: diferentes.

A: por isso que tu passou dois anos aprendendo a estudar, a decorar, a saber o quê que tem que fazer, qual oração tem que fazer pra cada coisa que curar. É?

OF8: é.

A: então é isso. Muito obrigado.

[00:21:40]

Áudio B

[00:00:10]

A: ô Pedrina tudo que tu sabe, que tu aprendeu pra fazer remédio, oração tu vai passar pra alguém?

OF8: Não.

A: Não? Por quê?

OF8: Não pode.

A: Não pode? Não, não estou dizendo pra mim e pro Manuel não. Pra tu não pode escolher alguém na tua casa, na tua filha? (...)

OF8A: (...) Sim, algum filho, neto, ensinar.

OF8: ensina meu filho, minha filha aquele (gordinho) [00:00:36]
OF8A: hã. Ensinar ela (...)

OF8: (...) ele sabe (...)

A: (...) ela já sabe, ela tem uma filha?

OF8: A Liliane

A: A Liliane você já ensina ela?

OF8: uhum

A: e ela já começou a atender também?

OF8: Já.

A: Já.

OF8: É tudo minha filha sabe (inint) [00:00:54] fazer remédio esse daqui doença.

OF8A: (inint) [00:01:00]

OF8: é.

A: eles já fazem os filhos dela.

OF8: eles sabem

A: Que aí é pra ficar pra outra geração que vão nascendo. Ele eles aprenderem a ensinarem e curarem também?

OF8: é.

A: É::... Não pode ser pra qualquer pessoa. Tem que ser tem que ter o dom pra saber aquelas orações?

OF8: É. Tem um caderno.

A: Ah isso que eu ia te perguntar. Eessas orações estão registrada, anotada?

OF8: Tá registrada.

A: É. E a::... mas tu também sabe na cabeça.

OF8: Sim.

A: É? Aí quando elas também estão registrada e tão em ticuna.

OF8: É.

A: É? Entendi. Está bom. Obrigado.

[00:01:51]
