# Supplementary material for: Building an explanatory model for snakebite envenoming care in the Brazilian Amazon from the indigenous caregivers’ perspective
Source: PLoS Negl Trop Dis. 2023 Mar 10;17(3):e0011172. doi: 10.1371/journal.pntd.0011172 (PMC10047533; doi:10.1371/journal.pntd.0011172)
Supplement: Attachment — Submitted filename: renamed_916c4.docx [file pntd.0011172.s003.docx]

REBUTTAL LETTER – CAREGIVERS’ MANUSCRIPT

Dear editor,

Thank you for considering our manuscript for review and for allowing us to make the necessary corrections. Below we respond point by point to the referees' recommendations.

Reviewer #1: -Are the objectives of the study clearly articulated with a clear testable hypothesis stated? Yes
-Is the study design appropriate to address the stated objectives? Yes
-Is the population clearly described and appropriate for the hypothesis being tested? Yes
-Is the sample size sufficient to ensure adequate power to address the hypothesis being tested? Since this is a qualitative study, sample size is adequate.
-Were correct statistical analysis used to support conclusions? No relevant
-Are there concerns about ethical or regulatory requirements being met? None
RESPONSE: Thanks for the comments.

Reviewer #2: There was a clear study aim and specific objectives; the qualitative design is appropriate; the study population was clearly defined. Given one of the stated study limitations (difficulty achieving data saturation), the sample size is acceptable; the analysis and interpretation of the study results were appropriate.  Ethical handling of the study was appropriate.
RESPONSE: Thanks for the comments.

--------------------

Reviewer #1: -Does the analysis presented match the analysis plan? Yes
-Are the results clearly and completely presented? Yes
-Are the figures (Tables, Images) of sufficient quality for clarity? Yes
RESPONSE: Thanks for the comments.

Reviewer #2: The analysis presented matched the analysis plan; the results clearly and completely presented; The pictorial presentation results in the figures were beautiful.
RESPONSE: Thanks for the comments.

--------------------

Reviewer #1: -Are the conclusions supported by the data presented? Yes
-Are the limitations of analysis clearly described? Yes
-Do the authors discuss how these data can be helpful to advance our understanding of the topic under study? There is a comment on discussion. See the general comment.
-Is public health relevance addressed? Yes, with in the relevant geographical area
RESPONSE: Thanks for the comments.

Reviewer #2: The conclusion was appropriate for the results obtained.

RESPONSE: Thanks for the comments.

--------------------

Reviewer #1: (No Response)

Reviewer #2: I will recommend that you ACCEPT for publication in the present form.
RESPONSE: Thanks for the comments.

--------------------

Use  this section to provide overall comments, discuss strengths/weaknesses of the study, novelty, significance, general execution and scholarship. You may also include additional comments for the author, including concerns about dual publication, research ethics, or publication ethics. If requesting major revision, please articulate the new experiments that are needed.

Reviewer #1: This paper describes the qualitative interview-based study on developing an explanatory model of indigenous healthcare communities for Snakebite envenoming patients from the perspective of indigenous caregivers in selected areas in Brazilian Amazon. The study has been conducted in a well-designed qualitative study method and the presentation of results are appropriate.

RESPONSE: Thanks for the comments.

Major comments
More details of the study area: Include a map of the stud area indicating the locations of the caregivers were?

RESPONSE: A map of the study area was included in this new version of the manuscript.

What is the basis of selecting these 8 caregivers and excluding others? Was it due to availability and accessibility or any other reason? Please include the basis of selecting the caregivers.

RESPONSE: Thanks for the comment. The sample of 8 caregivers was of convenience, and all were indicated by the health professionals who work in the units of these villages.

In what basis these questions were formulated? Describe the rationale of formulating these questions?

Under data analysis: Lines 225 to 229: “Interview data was analyzed to understand indigenous caregivers’ explanations in an analytical framework with three themes: i) Etiology; ii) Course of sickness (with three subthemes: onset of symptoms, pathophysiology, and severity and prognosis); and v) Treatment”
Describe how the data gathered from listed 20 questions was used to analyse under the above three themes?
Which questions were related to theme 1, 2 and 3?

RESPONSE: The script was built by a multidisciplinary team of researchers, including one with expertise in indigenous health, being indigenous himself, and with the participation of health professionals working in indigenous areas. The script is just a way of organizing the interview sequence, but many times it is not necessary to ask question by question, since a single question can lead the participant to speak broadly, answering more than what was explicitly requested. Thus, it is not always possible to specify the connection between a question and a specific theme. In a simplified way, the table below can answer the reviewer's request.

| **Question** | **Theme** |
| --- | --- |
| 1. In your village snakebites occur? | Etiology |
| 2. How often do they occur? | Etiology |
| 3. Have you ever seen snakebites occur? | Etiology |
| 4. Why do snakebites occur in your village? | Etiology |
| 5. Do snakebites have any meaning in your village? | Etiology |
| 6. When you find a snake what should be done? | Etiology |
| 7. When a snakebite occurs, what does the indigenous person feel? | Course of sickness |
| 8. What can happen to the indigenous person who was bitten by a snake? | Course of sickness |
| 9. What situations can aggravate snakebites? | Course of sickness |
| 10. What should be done immediately after snakebite? | Treatment |
| 11. What indigenous treatments are used for snakebites? | Treatment |
| 12. When the indigenous person is bitten, does he/she have to go to the hospital? To do what? | Treatment |
| 13. In which circumstances does the indigenous person need to go to the hospital? | Treatment |
| 14. How long after the snakebite does indigenous person normally go to the hospital? | Treatment |
| 15. What were the three most important snakebite cases you treated? | Course of sickness |
| 16. What does the indigenous person do to avoid being bitten by a snake? | Etiology |
| 17. How does the indigenous person protect the body so as not to be bitten? | Etiology |
| 18. If there were boots and chaps in the village would an indigenous person use them to protect from snakes? | Etiology |
| 19. Is there any protective equipment for the snakebites that the indigenous person would like to use but they do not have in the village? | Etiology |
| 20. To end the interview, can you give a summary of what you can and cannot do regarding snakebites? | Etiology, Course of sickness, and Treatment |

Since this manuscript is going to read and refer by the scientists and researchers, highly recommend to include few paragraphs under the discussion to discuss the appropriateness and or inappropriateness of caregiver’s perspectives into western science and medical practices. Eg, use of tourniquet is a dangerous and unaccepted practice in western science and medicine. Likewise, there are many inappropriate and unaccepted practices and believes were explored in this paper. It would be really interesting to discuss these under the discussion. It is understandable and not possible to discuss every finding here. But at least the significant and important findings need to de discuss.
RESPONSE: This is an interesting and complex question. In this work, we avoided as much as possible judging the procedures indicated by indigenous caregivers, classifying them as effective, non-effective or deleterious. We believe that it is not up to researchers, at the risk of a colonialist attitude, to make judgments about ancient practices, especially those of a mythical-religious nature. Therefore, the discussion of the manuscript approaches this theme in a topic of 'Symbolic efficacy', explaining this issue from an anthropological point of view. Except for proven deleterious procedures, such as the use of tourniquets, the other practices lack methodological resources to confirm or refute any benefit to the patient.

We discuss this point in the section of Limitations.

Other comments
Table 2: Details in the column ‘Presentation”: long descriptions of caregivers are not necessary. Recommend to reduce the word count and present in a point form avoiding sentences.
RESPONSE: We appreciate the recommendation. We respectfully ask the reviewer to allow the maintenance of this detailed description of each research participant, since in this column is the summary of the subject's life, his training for this social role and how he fits and sees himself in society. We consider that this information is very useful for the practices that they carry out in the current time.

239: A total of eight indigenous caregivers were conducted. An important word of “interviews” is missing in this sentence. This has to be edited as “A total of eight interviews were conducted with indigenous caregivers.”
RESPONSE: Sentence was changed accordingly.

270: SBES: The last “S” should in lower case format. SBEs
RESPONSE: Change was made accordingly.

263: Agents, should be change as species or snake
Throughout the manuscript: Word Envenoming and Envenomation have been used in different places. Suggest to use one terminology throughout the manuscript.
RESPONSE: Changes were made accordingly.

Reviewer #2: This is a well-written qualitative study, which tenaciously followed the 32-item COREQ checklist.

RESPONSE: Thanks for the comment.
